# Enzymolysis Modes Trigger Diversity in Inhibitor‐α‐Amylase Aggregating Behaviors and Activity Inhibition: A New Insight Into Enzyme Inhibition

**DOI:** 10.1002/advs.202404127

**Published:** 2024-09-05

**Authors:** Junwei Cao, Jifan Zhang, Ruibo Cao, Bin Zhang, Ming Miao, Xuebo Liu, Lijun Sun

**Affiliations:** ^1^ College of Food Science and Engineering Northwest A&F University Yangling Shaanxi 712100 China; ^2^ State Key Laboratory of Food Science and Resources Jiangnan University 1800 Lihu Avenue Wuxi Jiangsu 214122 China; ^3^ School of Food Science and Engineering South China University of Technology Guangzhou 510640 China

**Keywords:** α‐amylase inhibition, substrate enzymolysis mode, aggregate formation, binding interactions, inhibitors

## Abstract

Inhibitors of α‐amylase have been developed to regulate postprandial blood glucose fluctuation. The enzyme inhibition arises from direct or indirect inhibitor‐enzyme interactions, depending on inhibitor structures. However, an ignored factor, substrate, may also influence or even decide the enzyme inhibition. In this work, it is innovatively found that the difference in substrate enzymolysis modes, i.e., structural composition and concentration of α‐1,4‐glucosidic bonds, triggers the diversity in inhibitor‐enzyme aggregating behaviors and α‐amylase inhibition. For competitive inhibition, there exists an equilibrium between α‐amylase‐substrate catalytic affinity and inhibitor‐α‐amylase binding affinity; therefore, a higher enzymolysis affinity and concentration of α‐1,4‐glucosidic structures interferes the balance, unfavoring inhibitor‐enzyme aggregate formation and thus weakening α‐amylase inhibition. For uncompetitive inhibition, the presence of macromolecular starch is necessary instead of micromolecular GalG2CNP, which not only binds with active site but with an assistant flexible loop (involving Gly^304^‐Gly^309^) near the site. Hence, the refined enzyme structure due to the molecular flexibility more likely favors the inhibitor binding with the non‐active loop, forming an inhibitor‐enzyme‐starch ternary aggregate. Conclusively, this study provides a novel insight into the evaluation of α‐amylase inhibition regarding the participating role of substrate in inhibitor‐enzyme aggregating interactions, emphasizing the selection of appropriate substrates in the development and screening of α‐amylase inhibitors.

## Graphical abstract

For competitive inhibition, enzymolysis affinity or substrate concentration interferes balance between α‐amylase‐substrate catalytic affinity and inhibitor‐α‐amylase aggregating affinity. For uncompetitive inhibition, macromolecular substrate rather than micromolecular one causes adjustment of enzyme conformation, promoting inhibitor‐enzyme‐substrate aggregate formation, with substrate binding with active site and inhibitor binding with flexible loop.

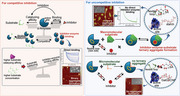

## 1 Introduction

As one of the glycoside hydrolases 13 family, α‐amylase functions in the digestion of starch to maltose, maltotriose and maltooligosaccharides by hydrolyzing the α‐configuration glycosidic bonds,^[^ ^1^ ^]^ which are further hydrolyzed to glucose by brush boarder glucosidases.^[^ ^2^ ^]^ Therefore, the increasing rate of blood glucose levels following a starch‐containing meal is highly related to α‐amylase activity. Notably, long‐term postprandial hyperglycemia presents a tough challenge for managing glucose metabolism, especially for preventing or treating some related diseases, like diabetes.^[^ ^3^ ^]^ Medically, some medicines, like acarbose (ACA) and voglibose are prescribed for type II diabetes patients to regulate the activity of key starch‐hydrolyzing enzymes, which, in the meanwhile, causes some side effects.^[^ ^3^, ^4^ ^]^ Therefore, lots of efforts have been done to develop the medicine supplements or substitutes.^[^ ^5^ ^]^

Native botanic secondary metabolites have been shown with the inhibition effects on starch‐hydrolyzing enzymes, in which a number of dietary polyphenols have been evaluated regarding their inhibitory activity against α‐amylase, such as tea polyphenols, tannins, phenolic acids, etc.^[^ ^6^, ^7^ ^]^ Among them, three mechanisms including competitive, uncompetitive and mixed‐type ones are mainly responsible for the enzyme inhibition, which differentiates the binding or aggregating behaviors between polyphenols and α‐amylase.^[^ ^8^, ^9^ ^]^ More importantly, the essential functional groups or moieties that contribute to polyphenol‐enzyme binding interactions have been explored, such as caffeoyl and galloyl moieties.^[^ ^10^, ^11^ ^]^

**Figure 1:**
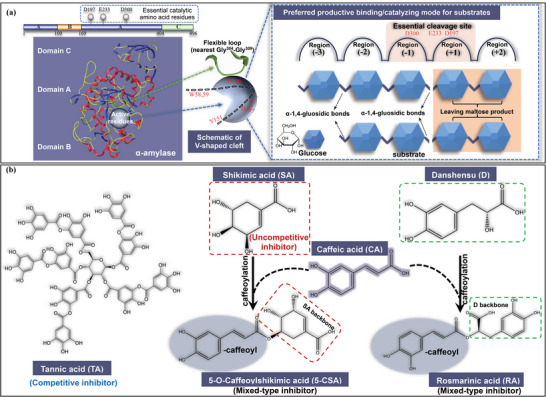
a) Domain schematic of full‐length α‐amylase with domain A in blue, domain B in orange, and domain C in green. The biggest domain A involves the active site. The “V‐shaped” active cleft of α‐amylase has five subsites, named as region −3, −2, −1, +1, and +2, which are responsible for the binding and catalyzing behaviors of α‐amylase for substrates. In particular, the essential cleavage site is distributed at the regions −1 and +1 that are located in the core area (deepest field). There are some key catalytic residues existing at this site, like D197, E233, and D300; therefore, the essential cleavage site is responsible for the preferred productive binding and catalyzing mode of α‐amylase (i.e., cleaving α−1,4‐glucosidic bonds at the non‐reducing ends and thus predominantly producing maltose). Additionally, the binding and catalyzing of α‐amylase for macromolecular natural substrates may also trigger the participation of other active regions and even flexible loops near the active cleft (e.g., the nearest loop Gly^304^‐Gly^309^). b) Molecular structures of the selected polyphenols and the related constitutional units including tannic acid (TA), shikimic acid (SA), caffeic acid (CA), 5‐*O*‐caffeoylshikimic acid (5‐CSA), danshensu D) and rosmarinic acid (RA). Among them, TA was proved as a typical competitive inhibitor and SA was an uncompetitive inhibitor. Both 5‐CSA and RA were the caffeoylated molecules with the backbone of SA and D, showing the mixed‐type inhibition in the present study.

As for the enzymolysis behavior, the functional structural region of α‐amylase is determined by its amino acid sequences. It is well known that there are three structural domains in the α‐amylase chain composed of 496 amino acids.^[^ ^12^ ^]^ The biggest domain A involves the active site that contains essential catalytic amino acid residues (**Figure** **1**a). The chloride ions in domain A and calcium ions in domain B are important for activating and maintaining the enzyme activity. Besides, the function role of domain C in the enzymatic process has not been fully identified. Notably, there are five subsites in the active pocket of α‐amylase, named region −3, −2, −1, +1, and +2, which are respectively composed of amino acid residues spreading over different positions.^[^ ^13^ ^]^ In particular, the essential cleavage site is distributed in the region −1 and +1 that are located in the core area (deepest field) of the “V‐shaped” active cleft of α‐amylase (Figure 1a). Some key catalytic residues are situated at this essential cleavage site, like Asp^197^, Glu^233^, and Asp^300^; therefore, this site is responsible for the preferred productive binding and catalyzing mode of α‐amylase for substrates, specifically cleaving α−1,4‐glucosidic bonds at the non‐reducing ends and thus predominantly producing maltose (Figure 1a). Notably, natural substrates of α‐amylase usually belong to macromolecules, like amylose, and amylopectin, both of which contain plenty of hydrolysable α−1,4‐glucosidic bonds. Therefore, other active regions where some representative fluorescent residues (like Trp^58^, Trp^59^, Tyr^151^, *etc*. at the cleft entrance) distribute, and even flexible loops that are adjacent to the active cleft (especially the nearest loop Gly^304^‐Gly^309^, Figure 1a) may also take part in the substrate hydrolysis by assisting the binding and catalyzing actions of the enzyme, producing reducing sugars.^[^ ^14^ ^]^

Recently, to increase research efficiency regarding α‐amylase activity, some artificial substrates have been designed, like 2‐chloro‐4‐nitrophenyl‐4‐*O‐β*‐D‐galactopyranosylmaltoside (GalG2CNP).^[^ ^15^ ^]^ Especially, there is only one hydrolysable glycosidic bond in GalG2CNP at the site between oligosaccharide and CNP, which is specifically designed for the essential cleavage site of α‐amylase. Then, due to the much less molecular weight (659.98 g mol^−1^) and hydrolysable site of GalG2CNP than native starch, both the enzymolysis behavior and product of the artificial GalG2CNP are relatively simple.

As the inhibition behaviors of inhibitors are directed against substrate hydrolyzing, the impacts of compositional form and amount of α−1,4‐glucosidic bonds in substrates on α‐amylase inhibition need to be shed light on. For this aim, in this work, the natural normal maize starch (NMS), the average molecular mass (M_w_) (Figure S1, Supporting Information), and chain length distribution (CLD) (Figure S2, Supporting Information) of which were respectively determined using SEC‐MALLS‐RI and GPC analysis, and artificial GalG2CNP were selected as substrates with different enzymolysis behaviors. Polyphenols that were determined with different inhibition characters including tannic acid (TA), shikimic acid (SA), caffeic acid (CA), 5‐*O*‐caffeoylshikimic acid (5‐CSA), danshensu (D) and rosmarinic acid (RA) were selected as inhibitors (Figure 1b). Then, the effects of substrate enzymolysis modes (structural composition and concentration of α−1,4‐glucosidic bonds) on α‐amylase inhibition of polyphenols were studied. Further, the direct and indirect aggregating interactions between polyphenols and α‐amylase were characterized in different inhibiting systems to elucidate how the change in the inhibitor‐enzyme aggregation caused the difference in the enzyme inhibition. Hence, through this study, the important role of appropriate selection of substrate digestion conditions in developing and screening of α‐amylase inhibitors is accordingly illustrated.

## 2 Results

### 2.1 Hydrolyzing Properties of Two Kinds of Substrates

**Figure 2:**
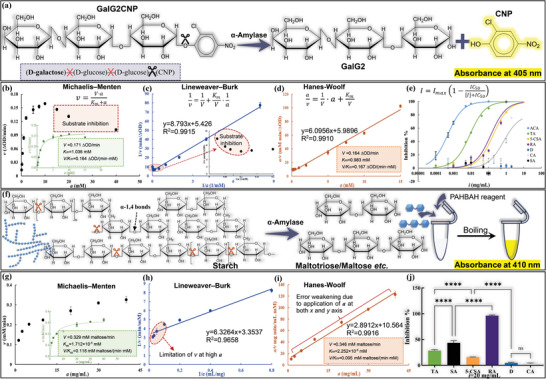
a) Specific enzymolysis of GalG2CNP only at the bond between GalG2 and CNP due to the _D_‐galactose modification of GalG2CNP. The absorbance of the generated CNP was monitored directly at 405 nm. b) The Michaelis‐Menten plot for analyzing the relationship between initial reaction velocity (*v*) and substrate concentration (*a*) regarding GalG2CNP hydrolyzing, with the “substrate inhibition” phenomenon at high substrate concentrations observed. The hydrolyzing constants including the maximum initial velocity (*V*, ∆_OD_/min), Michaelis constant (*K*_m_, mm), and *V*/*K*_m_ (∆_OD_/(min·mm)) were obtained through non‐linear regression of the Michaelis–Menten plot as shown in the inset plot. c) The Lineweaver–Burk (1/*v* against 1/*a*) plot for GalG2CNP hydrolyzing. The inset showed the dramatic ascending curve near the *y*‐axis due to the “substrate inhibition” phenomenon. d) The Hanes‐Woolf (*a/v* against *a*) plot for GalG2CNP hydrolyzing. The constants were determined faithfully as both the *x* (*a*) and *y* (*a*/*v*) axis involved *a*. e) Inhibitory activity of the selected polyphenols in the GalG2CNP hydrolyzing system, in which the IC_50_ values were fitted according to the inhibition percentages at different concentrations of these compounds. f) Simplified scheme of starch digestion. The amounts of leaving products were determined using the PAHBAH reagent approach, the absorbance of which was determined at 410 nm. g) The Michaelis–Menten plot for analyzing the relationship between *v* and *a* regarding NMS hydrolyzing, from which the hydrolyzing constants, including *V* (mm maltose/min), *K*_m_ (mm) and *V*/*K*_m_ (mm maltose/(min·mm)) were obtained. h) The Lineweaver–Burk plot for NMS hydrolyzing, in which the inaccuracy due to the restrictive *v* at the high starch concentrations was observed. (i) The Hanes‐Woolf plot for NMS hydrolyzing. The constants were determined faithfully as both *x* (*a*) and *y* (*a*/*v*) axis involved *a*. j) The inhibition ratios (%) of polyphenols (20 mg mL^−1^) against α‐amylase in NMS hydrolyzing system were obtained and statistically compared by one‐way ANOVA (**** *p* < 0.0001 and ^ns^ *p* > 0.05).

The digestion constants of GalG2CNP and normal maize starch (NMS) by α‐amylase were determined using the respective substrate depletion approach (**Figure** **2**a,f). For both substrate digestion, the non‐linear regression plot of initial reaction velocity (*v*) against substrate concentration (*a*) basically obeyed the Michaelis–Menten equation mode (Figure 2b,g), from which the maximal initial reaction velocity (*V*) and Michaelis constant (*K*_m_) were obtained. It was interestingly found that a high GalG2CNP concentration, however, went against its hydrolysis (Figure 2b), which was known as “substrate inhibition” phenomenon.^[^ ^16^ ^]^ This was caused by the fact that lots of GalG2CNP molecules competitively occupied the active cleft of α‐amylase, which affected the hydrolyzing reaction of the substrate molecules that had bound with the enzyme. Then, to further confirm the availability and accuracy of the obtained *V* and *K*_m_, two transformative models from Michaelis–Menten one, including Lineweaver–Burk and Hanes‐Woolf equations were applied. Due to the substrate inhibition phenomenon of GalG2CNP hydrolyzing and the restrictive *v* at a high starch concentration, there existed inaccuracy or error in Lineweaver–Burk model analysis for both substrate digestion, especially at the high *a* region (Figure 2c,h), which further caused some extents of inaccuracy in determining the corresponding catalytic constants (Figure S3, Supporting Information). On the contrary, the Hanes‐Woolf equation successfully decreased the error of Lineweaver–Burk one (Figure 2d,i), as both *x* (*a*) and *y* (*a*/*v*) variables of the equation contained *a*.^[^ ^17^ ^]^ This resulted in a good linear regression of the Hanes‐Woolf equation, with *R* ^2^ of both substrate digestion above 0.99.

Then, the values of *V* and *K*_m_ were extracted from the equation slope and intercept, both of which were similar to that obtained from the Michaelis–Menten equation for the respective substrate digestion (Figure 2b,d,g,i). Notably, the *K*_m_ value of NMS hydrolyzing was much lower than that of GalG2CNP hydrolyzing, suggesting that α‐amylase had a higher catalytic efficiency to NMS.

### 2.2 Inhibitory Activity of the Selected Polyphenols

Both substrate digestion by α‐amylase in the presence of polyphenols were conducted to characterize the enzyme inhibition. For this aim, the appropriate substrate, enzyme and inhibitor concentrations were delicately designed to obtain the detectable *v* and the accessible inhibition ratio. Generally, it was easier for the polyphenols to develop the inhibitory activity against GalG2CNP digestion by α‐amylase, as the polyphenol concentration required to reach the expected inhibition ratio was much lower in this hydrolyzing system (Figure 2e,j). Then, by fitting analysis of the correlation between the inhibition ratios and the phenolic concentrations, the IC_50_ values were obtained (Table S1, Supporting Information), which described the inhibitory activity of the polyphenols following the order of TA > RA ≈ 5‐CSA > CA ≫ SA & D. It should be noted that CA was not completely soluble at its concentration above 0.2 mg mL^−1^ (calculated as the final one); therefore, the practical IC_50_ value of CA was considered lower than the calculated one (i.e., the polyphenol actually had a stronger inhibitory activity than the determined one). Hence, the inhibitory activity of 5‐CSA and RA were derived from the caffeoyl moiety in two polyphenol molecules, as both moieties of SA and D were indicated with no inhibition effect (Figure 2e). As for NMS hydrolyzing system, it required much higher phenolic concentration to achieve the inhibition effect; therefore, it was difficult to obtain the IC_50_ values of the polyphenols (but the values would be much higher than that obtained in the GalG2CNP hydrolyzing system). Because of this, the enzyme inhibition ratio at one fixed polyphenol concentration (20 mg mL^−1^) was applied to describe the inhibitory activity, which followed the order of RA > SA > TA > 5‐CSA ≫ CA & D (Figure 2j), different from that in GalG2CNP hydrolyzing system. Especially, the most change in the inhibitory activity was found for SA, i.e., no and available enzyme inhibition in the respective hydrolyzing systems. It should be mentioned that the potential mechanism in the enzyme inhibition by non‐specific colloidal aggregating behaviors of polyphenols themselves was excluded in this work by the finding that addition of one surfactant (Triton X‐100) did not weaken the inhibition effects of the polyphenols (Figure S4, Supporting Information).^[^ ^18^ ^]^ Then, as α‐amylase inhibition in one digestion system results from the interactions among the individual participating molecules (including enzyme‐substrate, inhibitor‐enzyme, and/or inhibitor‐enzyme‐substrate interactions), the change in the enzyme inhibition observed in this study may be originally caused by different α‐amylase‐substrate interacting or catalytic affinity behaviors that further affected other molecular interactions in the digestion systems.

### 2.3 Inhibition Kinetics of the Selected Polyphenols

**Figure 3:**
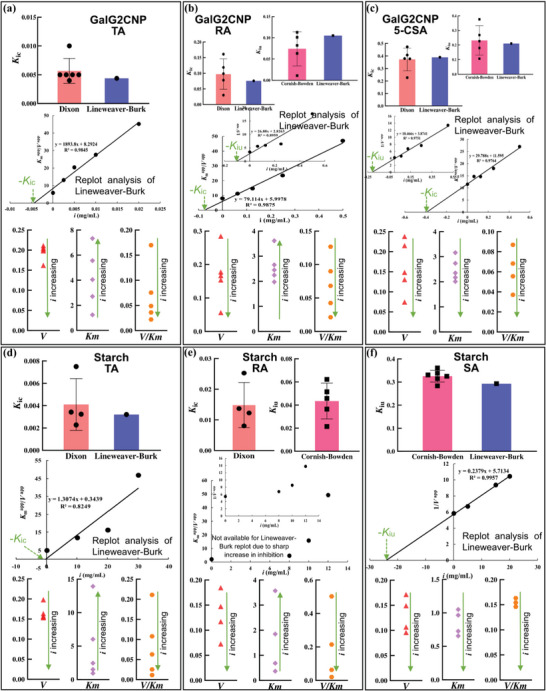
Comparison of *K*_ic_ (and/or *K*_iu_) values obtained from Dixon (and/or Cornish‐Bowden) plot and from Lineweaver–Burk plot for TA a), RA b) and 5‐CSA c) in GalG2CNP hydrolyzing system. Also, the changing tendency of the apparent *V*, *K*_m,_ and *V*/*K*_m_ in this hydrolyzing system was displayed. Then, the similar comparison and changing tendency for the above constants were applied for TA d), RA e) and SA f) in NMS hydrolyzing system.

To further characterize the inhibition behaviors of the polyphenols in different hydrolyzing systems, three typical mathematical models, including Dixon, Cornish‐Bowden and Lineweaver–Burk equations were applied to analyze the inhibition kinetics that describe the correlations between *v*, *a*, and *i* (inhibitor concentration) (**Figure** **3**; Figure S5, Supporting Information). It was found that for both substrate hydrolyzing systems, the respective Dixon plots of TA intersected at one point, while the Cornish‐Bowden ones paralleled with each other (Figure S5a,d, Supporting Information), indicating that TA acted as a competitive inhibitor of α‐amylase regardless of the substrate molecular structures. Besides, the types of Lineweaver–Burk plots (typically intersecting at *y* axis) and the changing trends of *K*_m_ (growing with TA concentration increasing) and *V* (basically remaining stable) also supported the competitive inhibition behavior of TA (Figure 3a,d; Figure S5a,d, Supporting Information). In this inhibition mode, TA could bind with the active site of α‐amylase, forming a competitive relationship with both substrates that shared the binding site with TA.^[^ ^19^ ^]^ On the contrary, for the inhibition kinetics of SA in NMS hydrolyzing system, the Dixon plots paralleled with each other, while the Cornish‐Bowden ones intersected at one point (Figure S5f, Supporting Information), indicating that SA was a typical uncompetitive inhibitor against starch digestion. Similarly, the paralleled Lineweaver–Burk plots, both the decreased *V* and *K*_m_, but the remained *V*/*K*_m_ further verified its uncompetitive inhibition (Figure 3f). This suggested that SA bound with α‐amylase‐NMS complex at the enzyme non‐active site, potentially forming an inhibitor‐enzyme‐substrate ternary complex and thus delaying the release of products, which is the typical character of an uncompetitive inhibition behavior.^[^ ^20^, ^21^ ^]^

Besides, for the inhibition kinetics of RA in two substrate hydrolyzing systems, both Dixon and Cornish‐Bowden plots intersected at one point and the Lineweaver–Burk plots intersected at the second quadrant (between *x* and *y* axis) (Figure S5b,e, Supporting Information). These plot profiles suggested that RA was a mixed‐type inhibitor, involving both competitive and uncompetitive inhibition characters.^[^ ^8^ ^]^ Therefore, the value of *V* decreased (the uncompetitive part) and that of *K*_m_ increased (the competitive part) with RA concentration increasing in both hydrolyzing systems (Figure 3b,e). The similar mixed‐type inhibition was also found for 5‐CSA against GalG2CNP hydrolyzing (the data for NMS hydrolyzing were not available due to the limited solubility of the polyphenol) (Figure 3c; Figure S5c, Supporting Information). It was interestingly found that there were irregular linear fitting (*R* ^2^ ≈0.6) in the Dixon and Cornish‐Bowden plots of RA at the high inhibitor concentrations (>8 mg mL^−1^) in the NMS hydrolyzing system (Figure S5e, Supporting Information). This may result from the easily happened acid dissociation of RA at high concentrations as this phenolic acid had the lowest p*K*_a_ value in all inhibitors, which sharply increased the enzyme inhibition due to the acidity. In other words, the low pH of RA solution (Table S2, Supporting Information) partially contributed to the enzyme inhibition effect.^[^ ^22^ ^]^ By contrast, as the acid dissociation extents of the other polyphenols were lower than RA at the same concentration as suggested by the higher determined pH and reported p*K*a (Table S2, Supporting Information), the contribution of changes in pH in the reaction system was indicted to contribute much less to α‐amylase inhibition of these inhibitors (Figure S6, Supporting Information). Also, the suitable fitting characters of the linear kinetics equations of the polyphenols apparently excluded the influence of pH value. Therefore, it was concluded that the inhibition effects of the polyphenols (especially for TA and SA) predominantly resulted from their direct or indirect interactions with the enzyme, instead of the acid dissociation of the inhibitors. Interestingly, the Lineweaver–Burk plot could well‐avoid the analysis error of RA (Figure S5e, Supporting Information) as it individually dealt with the correlation between a series of 1/*a* and the corresponding 1/*v* for each *i*,^[^ ^23^ ^]^ which excluded the interference of the pH change. These results indicated the necessity and efficiency of combination of multiple analysis models for α‐amylase inhibition kinetics. Furthermore, two important constants, including competitive (*K*_ic_) and uncompetitive (*K*_iu_) inhibition constants were obtained from the corresponding intersection points of the applied kinetics equations. It was found that the values of *K*_ic_ (or *K*_iu_) obtained from the Dixon plots (or the Cornish–Bowden ones) were similar to that obtained from the Lineweaver–Burk ones for each kinetics analysis of the polyphenols (Figure 3), indicating the effectiveness and accuracy of the calculated inhibition constants.

**Figure 4:**
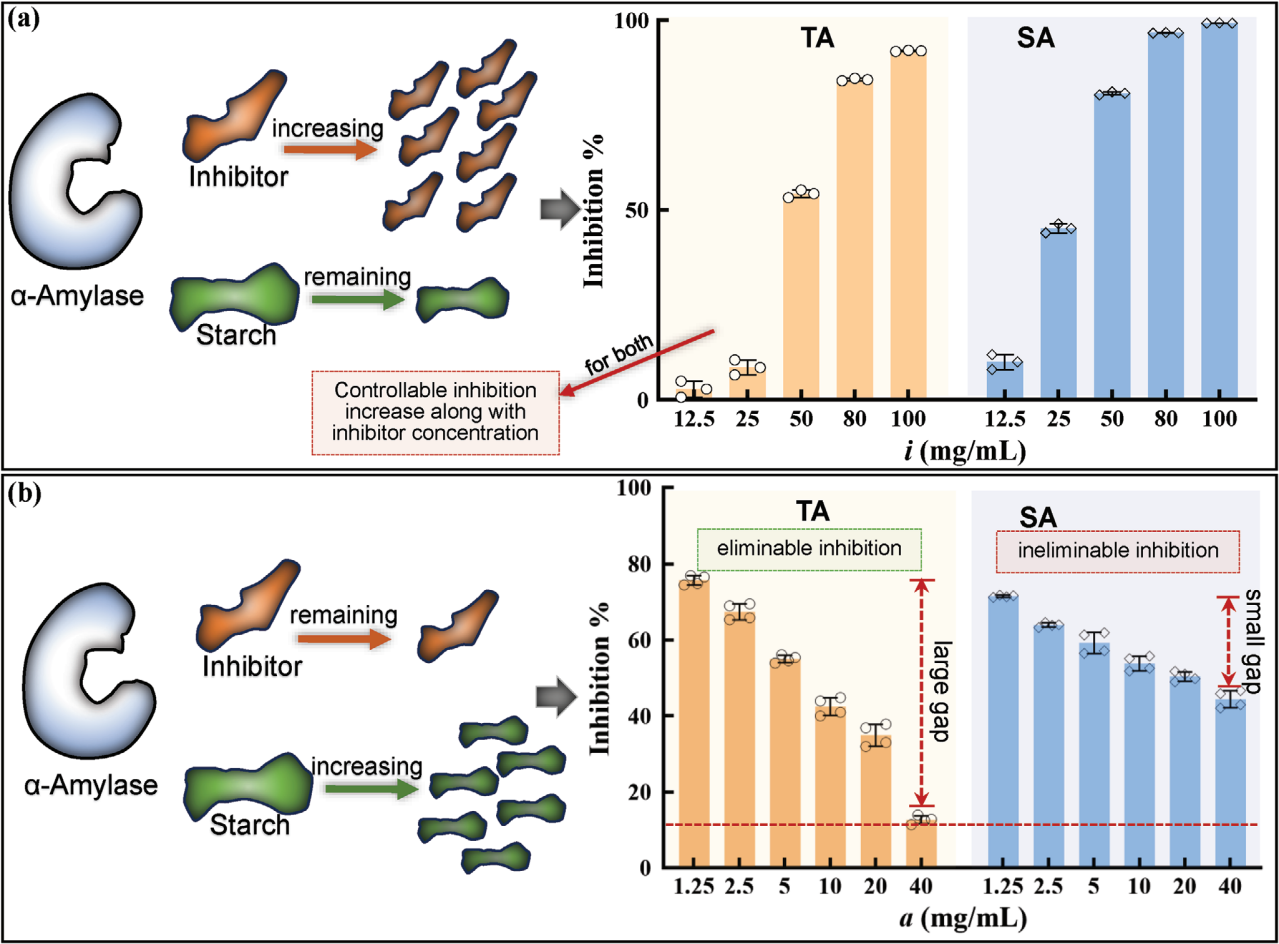
The changes in α‐amylase inhibition of TA and SA in the case that the inhibitor concentrations increased with starch concentration fixed a), and that starch concentration increased with the inhibitor concentrations fixed b), respectively.

According to the above inhibition kinetics, we selected two polyphenols, including TA and SA, as two representatives with the competitive and uncompetitive inhibition properties. Then, both the effects of changes in substrate and inhibitor concentrations on the enzyme inhibition were studied (**Figure** **4**). The results showed that the inhibition ratios of both polyphenols increased in a controllable mode with the polyphenol concentrations increasing (Figure 4a), suggesting the regularity of the reversible inhibition. Besides, increasing starch concentration was found to effectively impair the inhibition effect of TA rather than that of SA (Figure 4b), indicating the difference in the changes of binding behaviors among the substrate, enzyme and inhibitor in different inhibition‐type digestion systems.

### 2.4 Binding Interactions Between Polyphenols and α‐Amylase

#### 2.4.1 Fluorescence Quenching

**Figure 5:**
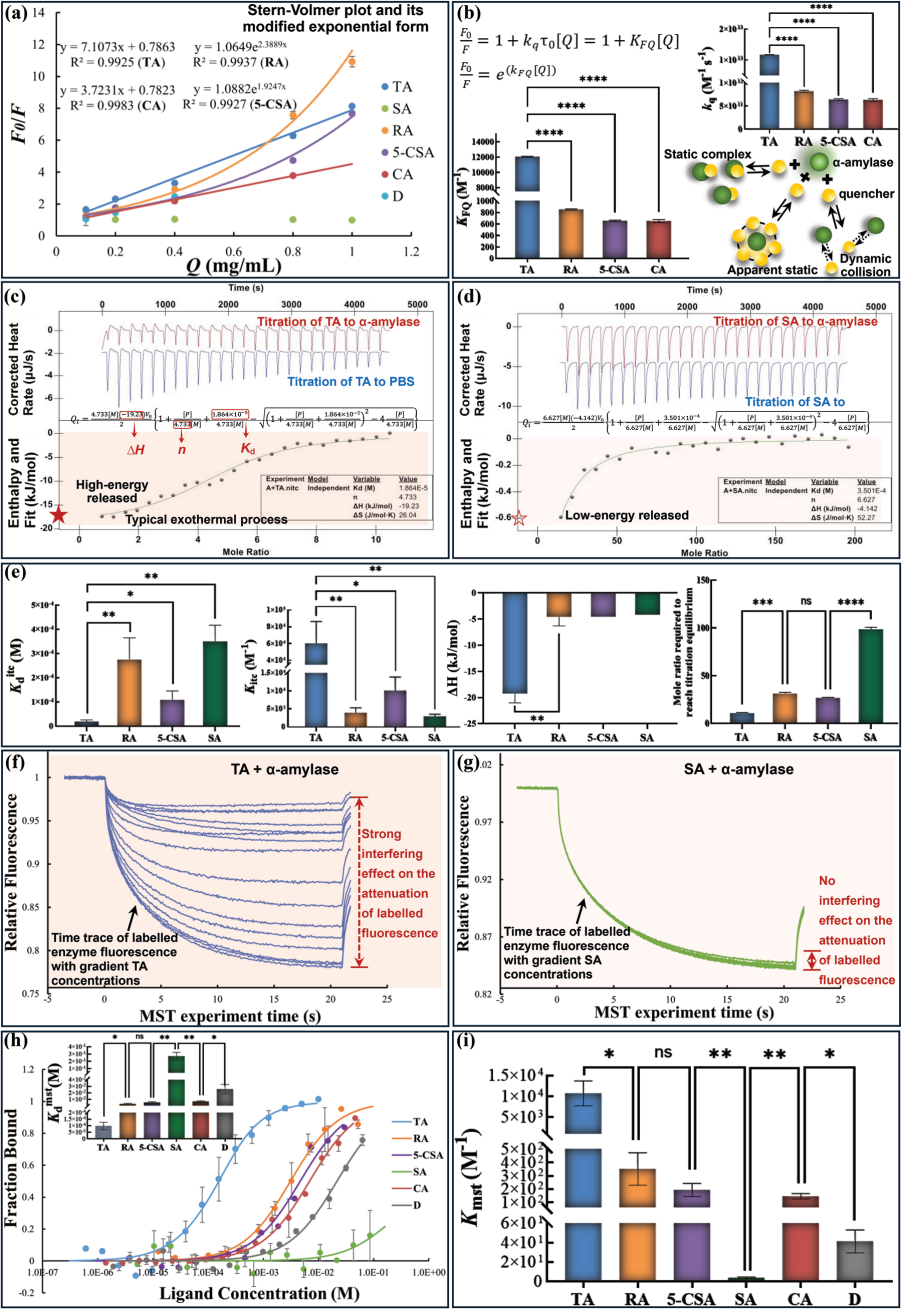
Characterization of the binding interactions between polyphenols and α‐amylase. a) The Stern‐Volmer or its exponential‐type plots were applied to analyze the quenching effects of polyphenols, from which the fluorescent quenching constant *K*_FQ_ (M^−1^) and the bimolecular quenching constant *k*_q_ (M^−1^ s^−1^) were calculated. b) The quenching constants of RA, 5‐CSA, and CA were statistically compared with that of TA using a t‐test, respectively (****p < 0.0001). The static quenching mechanism of polyphenols could be determined according to the obtained *k*_q_. The profiles of the thermodynamic information on ITC titration of TA c) and SA d) to α‐amylase, respectively. Both the titration procedures of polyphenols into α‐amylase (red curve) and that into PBS (blue curve) were performed to obtain the enthalpy changes caused by the polyphenol‐enzyme binding process by subtracting the dilution heat of polyphenols from the total titration heat. Then, the correlations between the enthalpy changes and molar ratios of polyphenols to α‐amylase were fitted using the independent (single‐site) binding model, from which the dissociation constant *K*_d_^itc^ (M), the association constant *K*_itc_ (1/*K*_d_^itc^, M^−1^), enthalpy value ∆*H* (kJ mol^−1^), and the mole ratio (ligand‐to‐enzyme) required to reach titration equilibrium were obtained. The related constants between two polyphenols were compared using a t‐test. The significant difference was labeled (****p < 0.0001, ***p < 0.001, **p < 0.01, *p < 0.05 and ^ns^ *p* > 0.05) e). The MST analysis curves for the binding affinity of TA f) and SA g) to α‐amylase, respectively, in which the polyphenols were shown with different interfering effects on the attenuation of labeled α‐amylase fluorescence at 16 gradient concentrations. From the time trace of MST curves, the dissociation constant *K*_d_^mst^ (M) values were obtained by fitting the fraction bound against the ligand concentration based on “*K*_d_ model” h). Then, the association constant, *K*_mst_ describes the binding affinity of the ligand to the enzyme, was calculated as 1/*K*_d_^mst^ (M^−1^) and statistically compared (**p < 0.01, *p < 0.05 and ^ns^ *p* > 0.05) i).

The presence of aromatic amino acid residues (indole or benzene rings) in α‐amylase, including Trp^58^, Trp^59^, and Tyr^62^, makes the enzyme emit a fluorescence spectrum under the excitation of ultraviolet light with certain wavelengths.^[^ ^24^ ^]^ When there exist exogenous aromatic substances in the enzyme solution like polyphenols, the exogenous aromatic rings would stack with the enzyme aromatic rings through *π‐π* conjugating forces in a parallel or vertical mode, which is able to “cover” the fluorescent properties of the enzyme aromatic residues.^[^ ^10^ ^]^ Accordingly, the phenomenon of “fluorescence quenching” occurs. Therefore, fluorescence quenching is an effective approach to characterize the binding interactions between polyphenols and α‐amylase (**Figure** **5**; Figures S7 and S8, Supporting Information), especially at the fluorescent residue region. Due to the lack of aromatic rings in SA (Figure 1b), the inhibitor was shown with no quenching effect on α‐amylase fluorescence (Figure S7d, Supporting Information). Besides, because the fluorescence spectrum of phenolic compound D itself overlapped with that of α‐amylase at λ_em_ ranging 300–350 nm (Figure S8f, Supporting Information), the quenching property of D could not be characterized as expected. Beyond the two compounds, other four polyphenols were shown with the quenching effects in a quencher concentration‐dependent manner, after subtracting the fluorescence spectrum of each polyphenol from that of its corresponding mixture with the enzyme (Figure S7a,b,c,e, Supporting Information). Then, the quenching data were further analyzed using the Stern‐Volmer or its exponential equations, from which the fluorescence quenching constant (*K*_FQ_) and the bimolecular quenching coefficient (*k*_q_) were calculated (Figure 5a,b). As shown, TA had the highest quenching efficiency with the largest *K*_FQ_ and *k*_q_ in all polyphenols (Table S3, Supporting Information), which was caused by the fact that there exist the most aromatic rings (10 ones) in its molecular structure (Figure 1b). To further describe the quenching behaviors of the polyphenols, the linear modified Stern‐Volmer (double log plotting) was applied to analyze the fluorescence data. It was found that the four polyphenols (TA, RA, 5‐CSA, and CA) that exhibited a relatively strong quenching effect well‐fitted this linear equation (Figure S9, Supporting Information). Then, the binding sites (*n*) for the quenching action were calculated ≈1 (Table S3, Supporting Information), which conformed with previous studies for most phenolic quenchers.^[^ ^25^ ^]^ Besides, it was found that the *K*_a_ values of TA and CA calculated by the linear modified equation were very close to their *K*_FQ_ values (Table S3, Supporting Information). However, there was a significant difference in the related results for RA and 5‐CSA. This may result from the existence of an apparent static quenching mechanism for RA and 5‐CSA with both quenching effect curves deviating upward to the *y* axis (Figure 5a).^[^ ^26^ ^]^ Meanwhile, the double‐log transformation of the Stern‐Volmer equation linearized the non‐linear quenching data of RA and 5‐CSA. Even though, both the constant values were basically presented with the similar order (Table S3, Supporting Information).

Additionally, the value of *k*_q_ normally distributes at ≈10^10^ M^−1^ s^−1^ for a typical dynamic quenching model that causes the quenching effect through the random molecular collisions between the quenchers and fluorescent molecules (Figure 5b).^[^ ^27^ ^]^ However, the *k*_q_ values of TA, RA, and 5‐CSA were much higher than this value (Table S3, Supporting Information), suggesting that the polyphenols quenched α‐amylase fluorescence in a static (or an apparent static) model. Interestingly, the hardly changed *τ*_0_ of α‐amylase in the absence and presence of these polyphenols through time‐resolved fluorescence spectroscopy study also supported the static quenching mechanism that tended to form a quencher‐enzyme complex (Figure S10, Supporting Information). This conformed with the competitive inhibition characters of the three inhibitors that bound with the enzyme active site (the typically competitive inhibition or the partially competitive character in the mixed‐type inhibition). Notably, although CA was also shown as a static quencher, as suggested by the relatively high *k*_q_ and remained *τ*_0_ (Table S3, Supporting Information), the interaction site for CA may mainly involve the areas of the enzyme aromatic residues, instead of the essential catalytic residues, which resulted in the little inhibition effect of the polyphenol.

#### 2.4.2 Thermodynamics Binding

In order to obtain the direct binding affinity of the polyphenols to α‐amylase, two thermodynamics analyses were applied (Figure 5; Figures S11 and S12, Supporting Information), including isothermal titration calorimetry (ITC) and microscale thermophoresis (MST), both of which work based on the thermodynamic characteristics of polyphenol‐amylase binding interactions.^[^ ^28^, ^29^ ^]^ It was found that the binding interactions between the polyphenols and α‐amylase were an exothermal process as the collected enthalpy changes during the ITC titration procedures were negative. As shown, both the high‐energy released in ITC (Figure 5c) and the strong interfering effect on the attenuation of labeled fluorescence in MST (Figure 5f) for TA‐enzyme binding analysis indicated the intensive molecular interactions between the two molecules. Unlike this, the low‐energy released and the hardly observed effect on the decrease in the labeled enzyme fluorescence suggested the feeble SA‐enzyme interactions (Figure 5d,g). Then, from the respective enthalpy curve of ITC and fluorophore thermophoresis curve of MST, two essential interaction constants were obtained, including *K*_d_^itc^ and *K*_d_^mst^ (Figure 5e,h). As defined, both constants indicate the dissociation degree of the ligand‐receptor complex;^[^ ^10^, ^29^ ^]^ therefore, the reciprocals of them (1/*K*_d_^itc^ and1/*K*_d_^mst^) indicate the association degree (binding affinity) of the ligand and the receptor, which are marked as *K*_itc_ and *K*_mst_ (Figure 5e,i), respectively. It was found that TA was calculated with the highest *K*_itc_ and *K*_mst_ in all phenolic compounds (Table S3, Supporting Information), indicating that the polyphenol had the strongest direct binding affinity to α‐amylase. Therefore, it required the least TA molecules to reach the binding saturation with α‐amylase. In contrast, the lowest *K*_itc_ and *K*_mst_ values of SA demonstrated that SA did not tend to bind with the enzyme directly. Both the binding affinity of TA and SA corresponded to their respective inhibition characters (competitive and uncompetitive). Additionally, as for RA, 5‐CSA, CA, and D, the binding affinity of four polyphenols to α‐amylase conformed with their inhibition effects. Therefore, it is concluded that the direct affinity of a phenolic inhibitor to α‐amylase is essential to its competitive inhibition character (involving the completely competitive character and the partially one of a mixed‐type inhibitor). However, it is another story for the direct binding affinity and the uncompetitive inhibition.

Notably, as the approaches that characterize the interactions between a ligand and a receptor in different ways, there existed both consistency and inconsistency regarding the binding parameters obtained from fluorescence quenching, ITC, and MST analyses. Specifically, the binding constant *K*_a_ (1.09 × 10^4^ M^−1^) of TA obtained from fluorescence quenching was close to *K*_mst_ (1.07 × 10^4^ M^−1^) and *K*_itc_ (5.36 × 10^4^ M^−1^) obtained from MST and ITC (Table S3, Supporting Information), and all three values were much higher than that of other polyphenols in the respective constant groups, which further confirmed the necessity in the association character of the competitive inhibitor with α‐amylase. On the other hand, the *n* value in fluorescence quenching (normally ≈1) that is described as the binding site was found different from that (*n*, ranging from 3 to 10 in this work) in ITC that is defined as stoichiometry (i.e., molecular ratio of ligand to receptor for direct binding). This resulted from the fact that the *n* in fluorescence quenching was mainly calculated based on the conjugation interactions between the aromatic rings of polyphenols and α‐amylase, while the *n* in ITC was fitted according to the comprehensive interactions between two molecules, involving hydrogen bondings, *π*‐conjugations, *etc*. Similarly, both the *n* and *K*_a_ for the quenching effect of CA were able to be obtained due to the existence of the above π‐conjugation, but the enthalpy curve of this polyphenol could not be fitted in ITC, because the heat caused by the direct CA‐enzyme binding interactions was too weak to be collected. Therefore, it is necessary and reasonable to comprehensively characterize the detail information of binding interactions between polyphenols and α‐amylase by combination of the approaches of fluorescence quenching, ITC, and MST, which describe the molecular interactions in different energy and perspectives.

#### 2.4.3 Aggregate Formation

**Figure 6:**
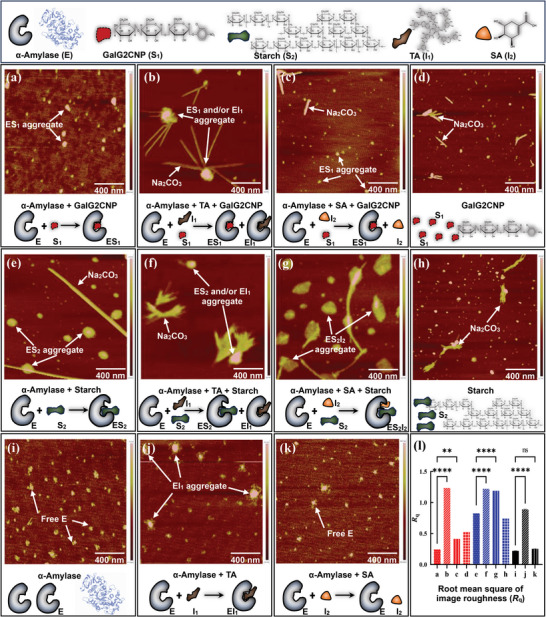
The AFM profiles of aggregating morphology of the interacting molecules in different inhibiting systems, including the respective aggregating observations of the mixtures of α‐amylase/GalG2CNP a), α‐amylase/TA/GalG2CNP b), α‐amylase/SA /GalG2CNP c), GalG2CNP d), α‐amylase/Starch e), α‐amylase/TA/Starch f), α‐amylase/SA/Starch g), Starch h), free α‐amylase i), α‐amylase/TA j), and α‐amylase/SA k). The corresponding mechanisms for the respective aggregating behaviors were illustrated, and the representative substances of the aggregate were labeled in each profile. Notably, the larger and brighter aggregate areas indicated the higher extent of the aggregating action. Then, the root mean square of morphological image roughness (*R*_q_) values were compared regarding the enzyme/polyphenol groups and enzyme/polyphenol/substrate groups, respectively (****p < 0.0001, ** *p* < 0.01 and ^ns^ *p* > 0.05) l).

To further characterize the binding behaviors among polyphenols, α‐amylase, and substrates, atomic force microscope (AFM) was applied to observe the microcosmic aggregate formed in different interacting systems (**Figure** **6**). The aggregate formation and its changes were described as the aggregation morphology observation and the root mean square of morphological image roughness (*R*_q_). It was found that compared to the α‐amylase sample that distributed uniformly on the mica plate (Figure 6i), there appeared obvious aggregate for TA/α‐amylase mixture (Figure 6j), suggesting the direct binding behavior of TA with the enzyme. Also, the observed aggregate still existed in the presence of two substrates (Figure 6b,f). This further accorded with the binding affinity of the polyphenol to α‐amylase that was obtained from the analysis results of fluorescence quenching, ITC, and MST. Conversely, as there was no significant difference between the molecular morphology and *R*_q_ value of SA/α‐amylase mixture and that of α‐amylase itself (Figure 6i,k), SA was indicated not to directly bind with the enzyme. Instead, this inhibitor was able to form the SA‐amylase‐starch ternary aggregate (Figure 6g), which exactly illustrated its uncompetitive inhibiting character in the NMS hydrolyzing system. Interestingly, this kind of inhibitor‐enzyme‐substrate aggregating behavior did not appear in the SA/α‐amylase/GalG2CNP mixture (Figure 6c), corresponding to the hardly observed enzyme inhibition of SA in the artificial substrate digestion system (Figure 2e).

**Figure 7:**
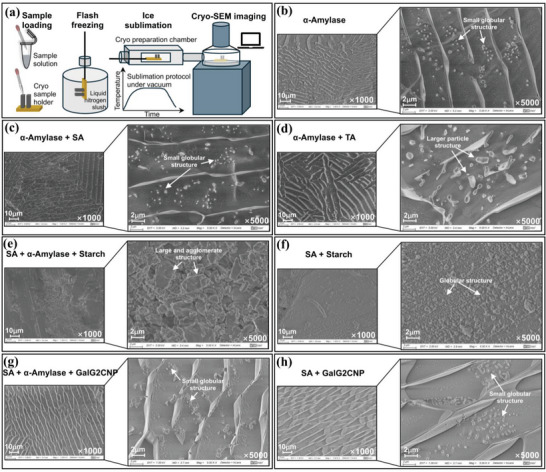
The Cryo‐SEM profiles of the morphology of particles formed in different binding systems. a) Schematic representation regarding the experimental protocol of Cryo‐SEM, including sample loading, flash freezing, ice sublimation, and Cryo‐SEM imaging. The respective morphological observations of the samples of α‐amylase itselfb), α‐amylase/SA c), α‐amylase/TA d), α‐amylase/SA/Starch e), SA/Starch f), α‐amylase/SA/GalG2CNP g) and SA/GalG2CNP h), in which the featured aggregates were labeled.

Then, to further verify the above aggregating behaviors observed in AFM, Cryo‐Scanning Electron Microscopy (Cryo‐SEM) was applied to capture the more precise profiles of the featured aggregates (**Figure** **7**), which allows the real‐time observation of the aggregating behaviors in the interaction systems by quick‐freezing of liquid nitrogen and also excludes the interferences of the ice particles on the aggregate structures by sublimation process of small ice crystals in a short time (Figure 7a). Specifically, the similar particle distribution observed in α‐amylase itself (Figure 7b) and in SA/enzyme mixture (Figure 7c) confirmed the indirect binding interactions between SA and α‐amylase, while the larger particles in TA/enzyme mixture supported the direct binding of TA to α‐amylase (Figure 7d). As for the ternary aggregating behaviors, the particle size (large and agglomerate structure) in SA/enzyme/starch mixture (Figure 7e) was much larger than that (small globular structures) in α‐amylase itself, SA/enzyme mixture, and SA/starch mixture (Figure 7f), confirming the formation of SA‐enzyme‐starch ternary complex. By contrast, the similar particle morphological characters (all small globular structures) in SA/enzyme/GalG2CNP mixture (Figure 7g), α‐amylase itself, SA/enzyme mixture, and SA/GalG2CNP mixture (Figure 7h), supported the fact that no ternary complex formed in the reaction system in the presence of the micromolecular substrate.

Both the AFM and Cryo‐SEM results indicate the role of substrate type in deciding the inhibitor‐enzyme‐substrate binding interactions as well as the corresponding uncompetitive inhibition. Conclusively, there appeared different molecular aggregation in different inhibiting systems, which was highly dependent not only on the inhibition characters but also on the substrate structures.

#### 2.4.4 Molecular Docking

**Figure 8:**
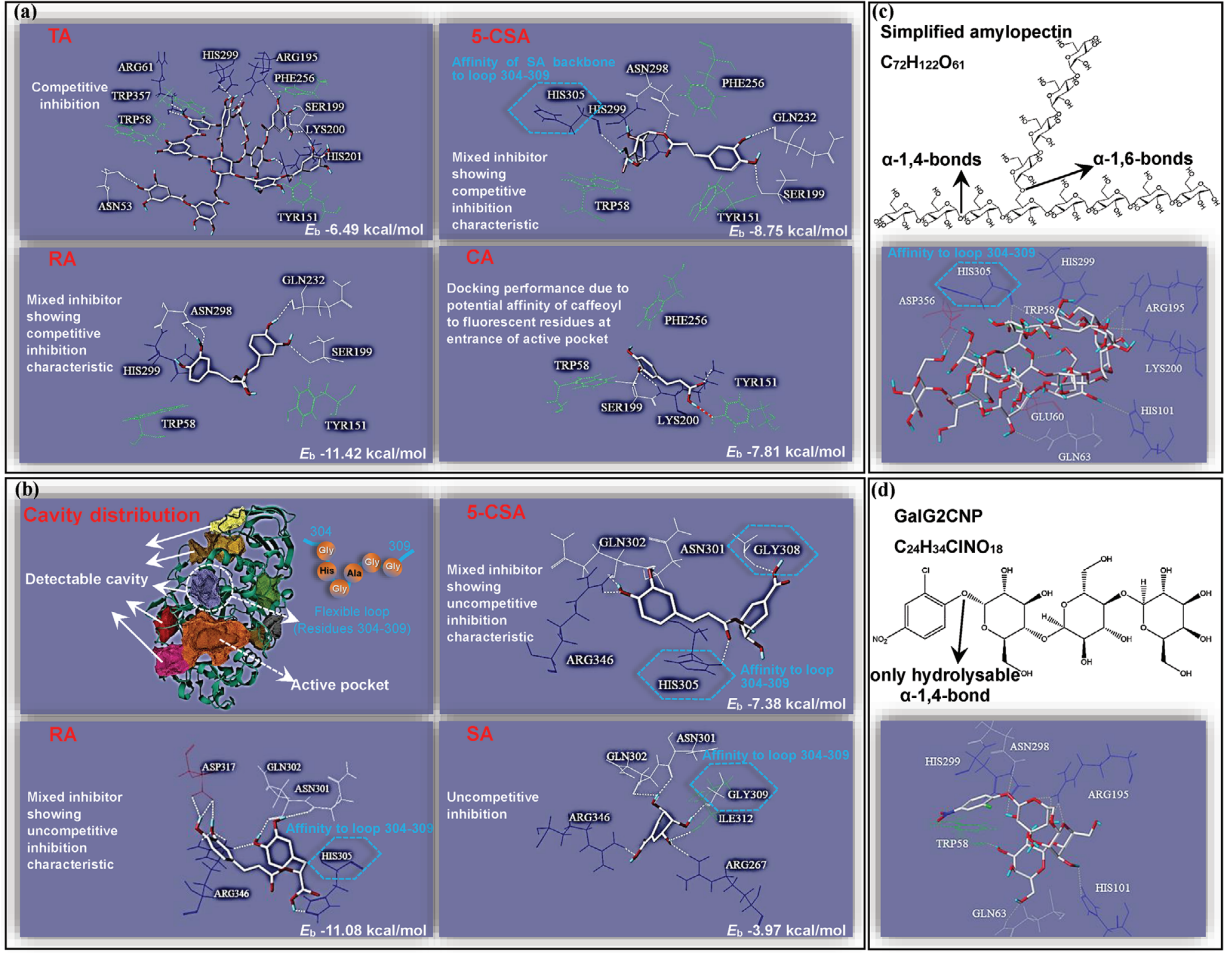
The molecular docking analyses simulating the interactions between α‐amylase and the polyphenols selected. Specifically, the docking procedure of TA was performed first, with the active pocket of α‐amylase selected as the preferred site due to the typical competitive inhibition of TA a). Then, this site was utilized for the docking of RA and 5‐CSA (partially competitive inhibition characteristic). Also, the docking of CA to the same site was conducted, since the caffeoyl moiety had a binding affinity with the fluorescent residues at the entrance of the active pocket. The non‐covalent forces were identified including the hydrogen bonds (white dash lines) and *π‐π* conjugation formed between polyphenols and aromatic residues (green color). b) For the polyphenols with uncompetitive inhibition characteristic, the detectable binding cavity in α‐amylase was predicted using CavityPlus. The cavity containing a flexible loop (Gly^304^‐His^305^‐Gly^306^‐Ala^307^‐Gly^308^‐Gly^309^) that was adjacent to the enzyme active site, was noted, as the residue His^305^ in this loop naturally appeared in the docking conformation with the SA backbone of 5‐CSA when the docking procedure of 5‐CSA with the active site of α‐amylase was performed. Subsequently, the cavity involving this flexible loop was used to dock with RA, 5‐CSA, and SA. Besides, the docking results of simplified amylopectin c) and GalG2CNP d) with α‐amylase were obtained, in which simplified amylopectin instead of GalG2CNP could bind not only with the active site but also with the Gly^304^‐Gly^309^ loop (His^305^) of α‐amylase.

The approach of molecular docking has been used widely to simulate the enzyme‐inhibitor and enzyme‐substrate molecular interactions regarding the binding site and non‐covalent forces.^[^ ^30^, ^31^ ^]^ First, the specific active cleft of α‐amylase that contained Glu^233^, Asp^197^, and Asp^300^ was selected as the docking site with TA due to the essential role of the residue area in the enzymolysis behavior and the competitive inhibition character of the polyphenol (**Figure** **8**a; Figure S13, Supporting Information). Then, the site was always selected as the docking one for other polyphenols to maintain the consistency of the condition parameters. By this way, it was found that TA was able to dock with the enzyme active site in an expected mode through hydrogen bondings with the amino acid residues at the target region, like Arg^195^, Ser^199^, Lys^200^, His^201^ (all near Asp^197^) and His^299^ (near Asp^300^) (Figure 8a). Besides, the aromatic rings of TA formed *π*‐stacking with the fluorescent residues of α‐amylase, like Trp^58^, Trp^357^, and Tyr^151^ (Figure 8a), which may cause the quenching effect on the enzyme fluorescence. Then, the available docking (or binding) of RA and 5‐CSA with the selected active site (Figure 8a) corresponded to the partially competitive inhibition character of both mixed‐type inhibitors. Additionally, there were some shared docking residues for caffeoyl moiety (in 5‐CSA and RA) and CA (that was composed of caffeoyl and hydrogen atom), including Ser^199^, Tyr^151^, and Trp^58^ (Figure 8a), indicating that caffeoyl moiety indeed had an affinity to α‐amylase active site. This further supported the fact that the partially competitive inhibition characters of RA and 5‐CSA were attributed to caffeoyl moiety, instead of the parts of SA and D. Notably, in addition to the specific catalytic cleft, there are some other cavities distributing in α‐amylase spatial structure at a non‐active site (Figure 8b), in which the flexible loop of Gly^304^‐His^305^‐Gly^306^‐Ala^307^‐Gly^308^‐Gly^309^ plays an important role in hydrolyzing of glycosidic chains regrading assisting the binding of α‐amylase with the long‐chain substrate.^[^ ^32^ ^]^ Interestingly, the SA backbone in 5‐CSA was naturally shown with the docking affinity to the Gly^304^‐Gly^309^ loop at His^305^ (Figure 8a), which was considered to contribute to the partially uncompetitive inhibition character of the polyphenol. Therefore, the enzyme site involving the flexible loop of Gly^304^‐Gly^309^ was selected as the docking one for further docking analysis for 5‐CSA, RA and SA. It was found that the three polyphenols were shown with the available affinity to the loop residues and its adjacent ones (Figure 8b), indicating the potential participation of the flexible loop in the uncompetitive inhibition characteristics. Additionally, to illustrate the important role of specific site of α‐amylase in binding with substrates, two simplified amylose and amylopectin molecules and GalG2CNP were designed for docking analysis with the enzyme (Figure 8c,d; Figure S13b, Supporting Information). Interestingly, both designed starch molecules could bind not only with the active site (such as His^299^, Asn^298^, Lys^200^ and His^201^) but also with the Gly^304^‐Gly^309^ loop (His^305^) of α‐amylase (Figure 8c; Figure S13b, Supporting Information), while GalG2CNP could only bind with the enzyme active site (such as His^299^, Asn^298^ and Arg^195^) (Figure 8d). Therefore, the shared and/or differentiated site of α‐amylase for binding with the substrates and polyphenols contributed to the variety in the enzyme inhibition characters and the corresponding changes.

## 3 Discussion

Inhibiting α‐amylase activity is an effective approach for regulating starch digestion and thus controlling postprandial blood sugar level. The enzyme inhibition by an inhibitor results from the binding interactions between them, which has been considered to be primarily affected by the essential physicochemical properties of both the inhibitor and enzyme.^[^ ^33^ ^]^ However, the impacts of physiochemical properties of substrates on α‐amylase inhibition remain unknown. In this work, we innovatively found that the difference in hydrolysis modes of substrates triggered the difference in the binding behaviors between α‐amylase and inhibitors as well as the corresponding inhibitory activity.

α−1,4‐Glucosidic bond is the specific hydrolyzing site of α‐amylase, which exists in the natural and artificial substrates in different linking forms.^[^ ^34^ ^]^ In the artificial substrate (GalG2CNP), the hydrolysable α−1,4‐glucosidic bond only accounts for one‐third of all glucosidic bonds (Figure 2a), while in the natural macromolecular starchy substrate (normal maize starch, NMS), α−1,4‐glucosidic bonds are the predominant linkages (>92%) (Figure 2f).^[^ ^35^ ^]^ Therefore, there are more selectivity and multiplicity of the hydrolysable α−1,4‐glucosidic bonds in NMS for catalytic action of α‐amylase. By this way, α‐amylase was shown with a much higher catalytic affinity to NMS than to GalG2CNP as indicated by the significantly lower Michaelis constant (*K*_m_) of NMS by five orders of magnitude. In other words, the hydrolysis process of NMS by α‐amylase was more difficultly disturbed by inhibitors than that of GalG2CNP. Therefore, a higher phenolic concentration was required to develop the enzyme inhibition effect in the starch digestion system (Figure 2e,j). From this view, when developing natural or artificial products as α‐amylase inhibitors, the enzyme inhibition determination of some effective reference compounds (like acarbose and white kidney bean extract, even though the IC_50_ values of which have been determined in many previous studies) are suggested to be simultaneously involved in the same digestion system to reasonably evaluate the inhibitory activity of the products.

As for a typical competitive inhibitor, the competitive relationship between the inhibitor and the substrate regarding binding with the enzyme active pocket causes the inhibition phenomenon.^[^ ^17^ ^]^ Therefore, both the concentrations of inhibitor and substrate are considered to play an important role in the performance of enzyme inhibition. The specific competitive inhibition character of TA in this study supported this conclusion. In detail, the well‐fitted IC_50_ curve of TA (Figure 2e) suggested a flexible and controllable inhibitor concentration‐dependent inhibition mode. Besides, the relatively large gap for α‐amylase inhibition ratios of TA at different starch concentrations (Figure 4b) indicated that the enzyme inhibition by this polyphenol would be gradually eliminated in the case that the starchy substrate concentration is infinitely increased. Hence, considering the diversity in α‐amylase inhibition of TA in GalG2CNP and NMS hydrolyzing systems, it is concluded that the IC_50_ index of a competitive inhibitor is not an inherent property. Instead, it is influenced by both the molecular type and concentration of the substrate. Precisely because of this, although the competitive‐type inhibitors of carbohydrate‐hydrolyzing enzymes are usually prescribed (like acarbose, voglibose, etc.) and applied for regulation of starch digestion, the refined and excessive starchy foods should still be avoided to reduce the competitive binding action of substrates and to develop the inhibitory activity of the medicines as much as possible. Interestingly, although TA was shown with diverse inhibitory activity at different substrate types and concentrations, the inhibitor was determined with a comparable competitive inhibition constant (*K*_ic_) in two different hydrolyzing systems (Figure 3a,d). This comparable *K*_ic_ phenomenon was also found for RA despite that it was determined as a mixed‐type inhibitor (Figure 3b,e). As defined, *K*_ic_ and its reciprocal (1/*K*_ic_) represent the dissociation and association coefficients between an enzyme and an inhibitor at the active site of the enzyme, respectively, which are determined and fitted at a series of both inhibitor and substrate concentrations.^[^ ^8^, ^36^ ^]^ Therefore, the determination and calculation of *K*_ic_ already involve the different binding effects among the substrate, enzyme and inhibitor. Because of this, *K*_ic_ (or 1/*K*_ic_) is considered as an intrinsic constant for the competitive inhibition behavior, which remains stable in different digestion systems.

Notably, there occurred totally different inhibition behaviors of SA in the digestion systems of GalG2CNP (inhibition hardly observed) and starch (uncompetitive inhibition type) (Figure 2e; Figure S5f, Supporting Information). This indicated that SA was more likely to bind with α‐amylase‐starch complex, delaying starch hydrolysis and reducing sugar release. Furthermore, in the SA/α‐amylase/starch hydrolyzing system, the increase in the substrate concentration could not effectively eliminate the inhibition effect (Figure 4b), suggesting that the amount of uncompetitive aggregate (SA‐amylase‐starch) was not easily reduced. Also, this phenomenon further confirmed that the main site of α‐amylase for SA binding (non‐active site) was different from that for starch binding and enzymolysis (active site).

**Figure 9:**
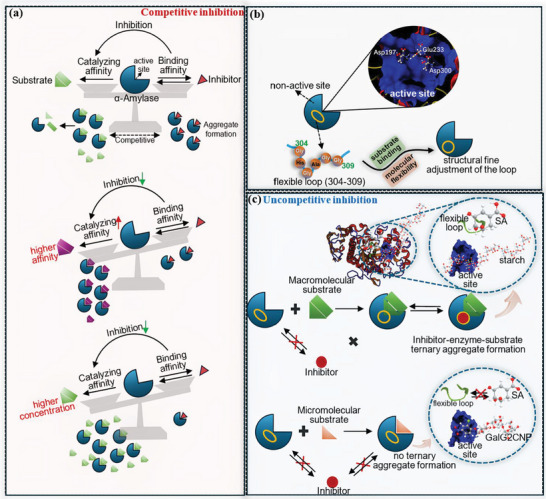
The diagram describing the mechanisms regarding the diversity in inhibitor‐α‐amylase aggregating behaviors and enzyme inhibition triggered by substrate enzymolysis modes. In detail, an equilibrium between α‐amylase‐substrate catalyzing affinity and polyphenol‐α‐amylase binding affinity was proposed for the competitive inhibition a). Thus, it was easily disturbed by a higher enzymolysis affinity and concentration of substrate, considering the competition between substrates and polyphenols for the active site of α‐amylase. Notably, the flexible loop (Gly^304^‐Gly^309^) near the active site would undergo the structural fine adjustment in response to the binding of substrate b). Because of this, the binding of macromolecular substrate with α‐amylase triggered the deformation of this loop, providing the binding site for the uncompetitive inhibitor and further promoting the formation of enzyme‐substrate‐inhibitor ternary complex, which was different from the complex formation in micromolecular substrate hydrolyzing system c).

From the characterization of inhibitor‐enzyme molecular interactions (fluorescence quenching, ITC, MST, and AFM), TA was indicated to directly bind with the independent site of α‐amylase in a gradual saturation mode, finally forming the TA‐amylase binary aggregate. The binding behavior of TA supported its competitive inhibition character. Therefore, taking the molecular interactions in the inhibitor/enzyme/substrate hydrolyzing system into account, there existed an equilibrium relationship between the TA‐amylase binding affinity and the α‐amylase‐substrate catalytic affinity, in which TA and the substrate (starch or GalG2CNP) competitively interacted with the essential catalytic amino acid residues (**Figure** **9**a). By this way, the change in the molecular affinity at the either side of the equilibrium would disturb the interaction balance and thus affect the enzyme inhibition effect. Specifically, α‐amylase was shown with a much higher enzymolysis or catalytic affinity to NMS than GalG2CNP; therefore, the hydrolyzing‐inhibition balance tended to incline to NMS hydrolyzing, causing a relatively weakened TA‐amylase binding affinity and thus a weaker enzyme inhibition in the starch digestion system (Figure 9a). Besides, due to the competitive relation between TA and substrate molecules, the change in the amounts of substances at either side of the hydrolyzing‐inhibition equilibrium would also disturb the balance and cause the change in the enzyme inhibition (Figure 9a). By this way, infinitely increasing the concentrations of starch and TA would respectively cause α‐amylase inhibition approaches disappearance and complete level in theory, in accordance with the inhibition observation in this work.

Interestingly, it was shown that SA was not able to directly bind with α‐amylase. Instead, the inhibitor tended to bind with α‐amylase‐starch complex, forming the SA‐amylase‐starch ternary aggregate. This is well‐conformed with its uncompetitive inhibition against starch digestion. It should be noted that the SA‐amylase‐GalG2CNP aggregate hardly formed, corresponding to the fact that SA did not show the inhibitory activity in GalG2CNP hydrolyzing system. In the enzymolysis process of substrates, the key amino acid residues at the active site of α‐amylase, especially Glu^233^, Asp^197^, and Asp^300^, play an important role in the catalytic action of the enzyme.^[^ ^14^ ^]^ Meanwhile, due to the high molecular weight of substrates and the flexible feature of enzymes (similar to the flexibility of protein molecules), there generally exist flexible loops at the enzyme non‐active site to assist the binding and release of macromolecular substrates.^[^ ^37^, ^38^ ^]^ Particularly, the flexible loop distributing at the amino acid residues of 304–309 sequence (Gly^304^‐Gly^309^ as the nearest loop of the enzyme active site) in α‐amylase has also been reported to develop the important booster action (assisting role) in the hydrolyzing process of starchy substrates.^[^ ^32^, ^39^ ^]^ Interestingly, both the simultaneous binding affinity of the simulated amylose and amylopectin (composed of 12 glucose units) to the flexible loop (especially to His^305^) in the docking result (Figure 8c; Figure S13b, Supporting Information) supported the previous finding. More importantly, due to the structural flexibility of Gly^304^‐Gly^309^ loop arising from the molecular flexibility of glycine, the binding of starch with α‐amylase could slightly adjust the microstructure or distribution of the loop (Figure 9b),^[^ ^40^ ^]^ which formed a changed loop spatial conformation that was more suitable for binding with SA. By this way, a SA‐amylase‐starch ternary aggregate formed with SA binding at the flexible loop and with starch binding at the active site of the enzyme (Figure 9c). However, as GalG2CNP is artificially designed and synthesized according to the catalytic specificity of α‐amylase to α−1, 4‐glucosidic bonds, the molecular structure of the substrate is simple and its molecular weight is low;^[^ ^41^ ^]^ therefore, the flexible loop of α‐amylase was not observed to participate in binding (docking) with GalG2CNP (Figure 8d). Hence, the micro‐conformation of the flexible loop remained untouched, which did not further favor the binding of SA with the enzyme loop (Figure 9c). Because of this, there appeared no SA‐amylase‐GalG2CNP aggregate, resulting in no enzyme inhibition of SA in the artificial substrate hydrolyzing system.

In this work, the variable factors for an α‐amylase inhibitor in a hydrolyzing system include the structural composition and concentration of α−1, 4‐glucosidic bond(s). More precisely, as for α−1, 4‐glucosidic bond structural composition, the bonds distribute in the primary chain of amylose and side linear chain of amylopectin in the starchy substrate, and the bond also exists in the analogous α−1, 4‐glucosidic‐CNP linkage in GalG2CNP. Therefore, the multiplicity and diversity of the enzymolysis site in two kinds of substrates are different. Besides, the change in starch concentration actually causes the change in the concentration of α−1, 4‐glucosidic bond. By this way, the structural composition and concentration of α−1, 4‐glucosidic bond in this work are considered as the hydrolysis modes of substrates. From the above results and mechanisms, it is concluded that the diversity in substrate hydrolysis modes trigger the different polyphenol‐amylase binding aggregation and inhibition effects. Specifically, for a competitive inhibitor that takes part in a competition with substrates regarding binding with α‐amylase active site, the substrate hydrolysis modes predominantly affect the balance between the catalytic affinity of α‐amylase to substrates and the binding affinity of the inhibitor to the enzyme (Figure 9a). In this case, the polyphenol‐enzyme aggregating effect is correspondingly weakened when the affinity and/or number of hydrolysable α−1, 4‐glucosidic bonds for α‐amylase action are increased; for an uncompetitive inhibitor that does not directly bind with α‐amylase, the substrate hydrolysis modes, especially when α−1, 4‐glucosidic bonds distribute in macromolecular starch, prompt the flexible loop in α‐amylase respond to the binding behavior of the enzyme to the macromolecular substrate, causing the structural fine adjustment of the loop and forming an inhibitor‐enzyme‐substrate ternary aggregate (Figure 9c). In this case, the ternary aggregating effect is not easily weakened when the substrate (or α−1, 4‐glucosidic bond) concentration increases, because the main sites on α‐amylase for binding with the inhibitor and for catalyzing the substrate do not share the same ones.

Furthermore, the diversity in α‐amylase inhibition of a polyphenol compound in different substrate hydrolyzing modes may provide some supporting significance for the screening process of phenolic α‐amylase inhibitors. In detail, the determined inhibitory activity of a polyphenol against α‐amylase in one kind of substrate hydrolyzing mode may not fully reflect its inhibition effect (taking the inhibition of SA as an example). The inhibition verification using multiple hydrolyzing systems is recommended. Then, as the analysis conditions (involving substrate types and concentrations) vary in individual research works,^[^ ^42^, ^43^ ^]^ the inhibitory activity of polyphenols are incomparable under different digestion conditions. Instead, both the same substrate hydrolyzing modes and the unified positive control are suggested in terms of α‐amylase inhibition comparison. Additionally, the aim for development of α‐amylase inhibitors is to regulate or delay the digestion velocity of starchy foods. Therefore, simulation or application of analogous dietary starch concentration or ratio is highly recommended when evaluating α‐amylase inhibition of a potential phenolic inhibitor. By doing this, it can avoid the inhibition variation caused by the skewing of the balance between enzyme‐substrate catalyzing affinity and enzyme‐polyphenol binding affinity due to the difference in starch concentration in the tube experiments and dietary starch digestion in vivo.

## 4 Conclusion

In this work, the impacts of substrate physicochemical properties on α‐amylase inhibition of native inhibitors were studied in GalG2CNP and NMS digestion systems with specific enzymolysis modes. The results supported that the different molecular binding aggregate in hydrolysis systems and inhibitory effects were related with substrate structural composition, concentration, as well as the inhibition types. In detail, the balance between enzymatic hydrolyzing and inhibitory behavior in a competitive inhibition mechanism was easily disturbed by α‐amylase‐substrate catalytic affinity and substrate/inhibitor concentrations, which resulted in the change in the intensity of inhibitory activity. However, the intrinsic competitive inhibition property (like the type and kinetics constant) remained unaltered due to the spontaneous affinity of this kind of inhibitor to the enzyme active site. Furthermore, the substrate structural composition was shown with a more obvious impact for an uncompetitive inhibition mechanism, as suggested by the aggregate behaviors among substrate, enzyme and inhibitor. Unlike the micromolecular artificial GalG2CNP, the binding of macromolecular starch with α‐amylase triggered the deformation of enzyme flexible loop and further promoted the participation of uncompetitive inhibitor in binding with substrate‐enzyme complex. In conclusion, the aggregating behavior of an inhibitor with α‐amylase in the absence of substrates only depends on the inhibitor molecular structure, while in substrate digestion systems, the inhibitor‐enzyme aggregating interactions also highly rely on substrate enzymolysis mode, including structural composition and concentration of substrate α−1,4‐glucosidic bonds. Therefore, the physicochemical properties of substrates should be considered as an essential impact factor when evaluating the inhibitory potentials of a natural or designed inhibitor against carbohydrate‐hydrolyzing enzymes.

## 5 Experimental Section

### Materials

Porcine pancreas α‐amylase (A6255, PMSF treated, Type I‐A), phosphate‐buffered saline (PBS) tablet, TA, and PAHBAH reagent were purchased from Sigma–Aldrich Co. (St Louis, USA). SA, D, and RA were purchased from Lemeitian Biological Technology Co., Ltd (Sichuan, China). 5‐CSA was got from Jingcuitiancheng Biotech. Co., Ltd (Sichuan, China). CA were obtained from Yuanye Bio‐Technology Co., Ltd (Shanghai, China). GalG2CNP was purchased from Tianyuan Biotech. Co. (Shanghai, China). Other chemical agents were analytic grade.

### Hydrolyzing Properties of Two Kinds of Substrates

The hydrolyzing constants of two substrates by α‐amylase were determined according to the respective digestion approaches.^[^ ^11^, ^35^ ^]^ For GalG2CNP digestion, the substrate GalG2CNP stock solution (50 mm) and α‐amylase (6 U mL^−1^) solution were prepared freshly in PBS. First, 20 µL of α‐amylase and 20 µL of 10% DMSO (in PBS) were incubated for 10 min in a 96‐well plate. Then, the reaction was initiated by adding GalG2CNP solutions, presenting a series of final substrate concentrations of 0.125–40 mM. The total reaction volume was 200 µL. The change in the absorbance values (∆_OD_) of the reaction solution was monitored at 405 nm for 12 min at the interval of 4 min. The initial reaction velocity (*v*) was obtained by displaying the plot of ∆_OD_ against time (∆_OD_/min). As for NMS digestion, the concentration of the stock gelatinized starch solution was 40 mg mL^−1^. Then, an equal volume (50 µL) of α‐amylase (20 U mL^−1^) and 10% DMSO (in PBS) were preincubated for 10 min. After that, 4 mL of gelatinization starch solution (1.25–40 mg mL^−1^) was added to the preincubated mixture to trigger the reaction at 37 °C. At 4 min intervals of the digestion process in 12 min, 300 µL of the reaction solution was respectively withdrawn and added into 300 µL of 0.3 M Na_2_CO_3_ solution to stop the reaction. The amounts of reducing sugars (maltose equivalents) produced in the reaction samples were determined using the PAHBAH reagent approach as established previously according to a standard curve of maltose solutions.^[^ ^44^ ^]^ The absorbance value of each sample was recorded at 410 nm, and *v* of starch digestion was calculated from the correlation of maltose equivalent production against digestion time (mM maltose/min). Then, the obtained *v* values were analyzed graphically using Michaelis–Menten (1), Lineweaver–Burk (2), and Hanes‐Woolf (3) equations as follow:^[^ ^17^, ^23^ ^]^

(1) $$v\;=\frac{V\times a}{K_{m}+a}$$

(2) $$\frac{1}{v}=\frac{1}{V}+\frac{K_{m}}{V}\times \frac{1}{a}$$

(3) $$\frac{a}{v}=\frac{1}{V}\;\times a+\frac{K_{m}}{V}$$

where, *V* was the maximum *v*; *a* was the substrate concentration; *K*_m_ was the Michalis constant.

### Inhibitory Activity of the Selected Polyphenols

The inhibitory activity of polyphenols was assessed by replacing 10% DMSO (in PBS) with the same volume of polyphenols in two digestion systems as described above. In detail, for GalG2CNP digestion system, the polyphenols with gradient concentrations were prepared in 10% DMSO. α‐Amylase and polyphenols were pre‐incubated, followed by the addition of 1 mM GalG2CNP. The total reaction volume was 200 µL. The change in absorbance was recorded during the digestion process to obtain *v*. For NMS digestion system, the concentrations of gelatinized starch and polyphenol solutions were 10 and 20 mg mL^−1^, respectively. The value of *v* was obtained as the slope of the plot of maltose equivalent production against digestion time. Then, the inhibition (%) of each polyphenol was calculated using equation (4) for both digestion systems. Subsequently, for GalG2CNP digestion system, the IC_50_ values were fitted according to the inhibition percentages of polyphenols at different concentrations using equation (5) as follows:^[^ ^11^ ^]^

(4) $$I\left(\%\right)=\left(1-\frac{v}{v_{0}}\right)\times 100$$

(5) $$I=I_{\max}\;\left(1-\frac{IC_{50}}{[I]+IC_{50}}\right)$$

where, *v*_0_ and *v* were the initial reaction velocity without and with inhibitor; *I*_max_ was the maximum inhibition; [*I*] was the inhibitor concentration.

### Inhibition Kinetic of the Selected Polyphenols

Taking the detectable inhibition into account, the inhibition kinetics of TA, RA, and 5‐CSA were conducted when GalG2CNP was used as the substrate at four concentrations (3, 2, 1, and 0.5 mM). Meanwhile, the kinetics results of TA, RA, and SA were obtained in NMS digestion system (15, 10, 5, and 2.5 mg mL^−1^). Then, after calculating the *v* values at different polyphenol (*i*) and substrate (*a*) concentrations, the correlations between the parameters were analyzed using Dixon (6), Cornish‐Bowden (7) and Lineweaver–Burk (2) equations to obtain the inhibition types and the related inhibition constants, including the competitive inhibition constant (*K*_ic_), the uncompetitive inhibition constant (*K*_iu_), and the apparent values of *V* and *K*_m_ as follows:^[^ ^8^, ^36^ ^]^

(6) $$v=\frac{V\times a}{K_{m}\left(1+\frac{i}{K_{\mathrm{ic}}}\right)+a}\;$$

(7) $$\frac{v}{a}=\frac{V}{K_{m}\left(1+\frac{i}{K_{\mathrm{ic}}}\right)+a\left(1+\frac{i}{K_{\mathrm{iu}}}\right)}\;\;$$

### Characterization of Binding Interactions Between Polyphenols and α‐Amylase—Fluorescence Quenching

The interactions between α‐amylase and the polyphenols were first characterized by the approach of fluorescent quenching using an RF‐6000 fluorophotometer (Shimadzu, Japan). Specifically, an α‐amylase solution was prepared in PBS with the enzyme concentration of 9 U mL^−1^, and the polyphenol solutions were prepared in 10% DMSO with gradient concentrations ranging 0.1–1.0 mg mL^−1^. Then, 400 µL of α‐amylase was mixed with 20 µL of polyphenols or 10% DMSO. Simultaneously, the samples mixing 400 µL of PBS with 20 µL of polyphenols were also prepared. The above mixtures were incubated for 10 min. Subsequently, each mixture was withdrawn into a quartz cuvette and scanned with the following parameters: excitation wavelength at 282 nm, emission wavelength ranging from 300 to 500 nm, and both slit widths at 10 nm. The fluorescence quenching constant (*K*_FQ_) and bimolecular quenching constant (*k*_q_) were obtained by applying the Stern‐Volmer equation (8) or its modified form (9) as follows:^[^ ^27^ ^]^

(8) $$\frac{F_{0}}{F}=\;1+k_{q}\tau_{0}\;\left[Q\right]=\;1+K_{\mathrm{FQ}}[Q]$$

(9) $$\frac{F_{0}}{F}=e^{(K_{\mathrm{FQ}}[Q])}\;$$

where, *F*_0_ and *F* were the maximal fluorescence intensity without and with the polyphenols, respectively, and [*Q*] was the quencher concentrations. As for the lifetime of the fluorophore for α‐amylase (*τ*_0_), it was obtained by performing time‐resolved fluorescence spectroscopy using a fluorimeter (Fluorolog‐QM, HORIBA).^[^ ^45^ ^]^ The fluorescence decay traces of α‐amylase in the presence of polyphenols were also obtained at *λ*_ex_ = 305 and *λ*_em_ = 390 nm.

Additionally, to better understand the binding behaviors based on the quenching effects, the fluorescent data were further analyzed by one additional modified Stern‐Volmer equation as follows:^[^ ^26^ ^]^

(10) $$\log\;\left(\frac{F_{0}-F}{F}\right)=\;\log K_{a}+n\log[Q]$$

where, *K*_a_ was the apparent binding constant and *n* was the number of binding sites.

### Characterization of Binding Interactions Between Polyphenols and α‐Amylase—Isothermal Titration Calorimetry (ITC)

The micro‐calorimetric titration of polyphenols to α‐amylase was performed using a Nano ITC instrument (TA, US) to obtain the thermodynamic information of binding interactions between two molecules.^[^ ^10^ ^]^ In detail, both α‐amylase and phenolic solutions were prepared in PBS with the respective concentrations of 2 and 4 mg mL^−1^. Then, the thermodynamic data regarding the titration of 50 µL of polyphenols in the syringe into 300 µL of α‐amylase in the cell were collected at 25 °C. A total of 25 titration injections (2 µL of each injection) were conducted, with the time interval between each injection lasting 180 s. Meanwhile, the control procedures for the titration of polyphenols into PBS were also performed. After that, the corrected enthalpy as a function against ligand‐to‐enzyme molar ratios was fitted using the independent binding model equation (11) as follows, from which the dissociation constant (*K*_d_^itc^), the association constant (*K*_itc_, 1/*K*_d_^itc^), enthalpy value (Δ*H*_itc_) and molar ratios of protein‐ligand interaction (*n*) were obtained.^[^ ^28^ ^]^

(11) $$\begin{array}{cc} Q_{i} & =\frac{n\left[M\right]\Delta H_{\mathrm{itc}}V_{0}}{2}\\ & \;\times \left\{1+\frac{\left[P\right]}{n\left[M\right]}+\frac{K_{d}^{\mathrm{itc}}}{n\left[M\right]}-\sqrt{{\left(1+\frac{\left[P\right]}{n\left[M\right]}+\frac{K_{d}^{\mathrm{itc}}}{n\left[M\right]}\right)}^{2}-4\frac{\left[P\right]}{n\left[M\right]}}\;\right\} \end{array}$$

where, *Q*_i_ was the heat signal collected in the titration; [*M*] and *V*_0_ were α‐amylase concentration and volume in the sample cell, respectively; [*P*] was the polyphenol concentration in syringe.

### Characterization of Binding Interactions Between Polyphenols and α‐Amylase—Microscale Thermophoresis (MST)

The molecular binding behaviors of polyphenols with α‐amylase were further characterized by microscale thermophoresis (MST, Monolith NT.115, Nano Temper Technologies, Munich, Germany) assay. The detail fluorescent labeling procedure for α‐amylase (333 nM) was performed according to the instructions of His‐Tag Labeling Kit RED‐tris‐NTA 2nd Generation (MO‐L018). Polyphenol solutions with 16 gradient concentrations were prepared in PBS. Then, 2 µL of fluorescent labeled α‐amylase was mixed with 2 µL of each polyphenol solution, followed by incubation for 10 min. Subsequently, all the mixtures were transferred to standard capillaries (MO‐K022, NanoTemper Technologies) and placed onto a capillary tray by the concentration gradient order. The measurement was conducted by an infrared light on‐off circulation, with 40% excitation power, medium MST‐power, and Nano‐red excitation type. The polyphenol‐enzyme binding fitted curves were obtained based on “*K*_d_ model” using equation (12) in MO. Affinity Analysis software to extract the dissociation constant (*K*_d_^mst^) as well as its reciprocal (*K*_mst_, 1/*K*_d_^mst^).^[^ ^29^ ^]^

(12) $$\begin{array}{c} \frac{\left[BL\right]}{\left[B_{0}\right]}=\frac{\left(\left[L_{0}\right]+\left[B_{0}\right]+K_{d}^{\mathrm{mst}}\right)-\sqrt{\left({\left(\left[L_{0}\right]+\left[B_{0}\right]+K_{d}^{\mathrm{mst}}\right)}^{2}-4\left[L_{0}\right]\left[B_{0}\right]\right)}}{2\left[B_{0}\right]} \end{array}$$

where, [*BL*] was the concentration of bound complex; [*B*_0_] and [*L*_0_] were the concentrations of total enzyme and ligand, respectively.

### Characterization of Binding Interactions Between Polyphenols and α‐Amylase—Atomic Force Microscope (AFM)

The aggregate morphology of interacting molecules in different inhibiting systems were obtained using Multimode 8 atomic force microscope (Bruker Co., Santa Barbara, USA).^[^ ^46^ ^]^ For AFM analysis, α‐amylase, polyphenol (TA and SA), and substrate (NMS and GalG2CNP) solutions were prepared in PBS with the respective concentrations of 8, 10, and 20 µg mL^−1^. Then, the samples of α‐amylase, α‐amylase/polyphenol mixtures, α‐amylase/substrate mixtures, and α‐amylase/polyphenol/substrate mixtures were respectively prepared with the equal volume of each substance. Notably, for the samples of α‐amylase/substrate mixtures and α‐amylase/polyphenol/substrate mixtures, GalG2CNP and NMS solution were added to the enzyme and to the preincubated mixtures of enzyme‐polyphenol, respectively. Subsequently, Na_2_CO_3_ (0.3 mM) was added immediately to stop the reaction. Then, 10 µL of each sample was loaded onto to mica materials and dried overnight. The sample profiles were recorded using AFM in a tapping mode with 0.997 Hz scanning frequency. The image roughness (*R*_q_) of samples were analyzed by NanoScope Analysis V1.10.

### Characterization of Binding Interactions Between Polyphenols and α‐Amylase—Cryo‐Scanning Electron Microscope (Cryo‐SEM)

To further characterize the featured complex formation in different systems, Cryo‐Scanning Electron Microscopy (Cryo‐SEM) was applied for the direct observation of structures in hydrated samples under cryogenic and vacuum conditions.^[^ ^47^ ^]^ Specifically, α‐amylase, polyphenols (TA and SA), and substrate (NMS and GalG2CNP) solutions were prepared in PBS with the concentrations of 22, 80, and 400 µg mL^−1^, respectively. The samples similar to AFM analysis were prepared freshly before experiment. Then, each sample was loaded onto cryo sample holder and frozen by liquid nitrogen slush. After that, the samples were transferred into a cryo preparation chamber. A smooth surface of each sample was created for Cryo‐SEM imaging with a diamond knife. Subsequently, the samples were treated with an ice sublimation protocol (5 min) under vacuum. Then, the samples were imaged and recorded using the InLens detector (Sigma 300, ZEISS, Germany) at 3 kV.

### Characterization of Binding Interactions Between Polyphenols and α‐Amylase—Molecular Docking

The binding interactions between α‐amylase and polyphenols were predicted using Sybyl 2.0 software.^[^ ^11^ ^]^ The crystal structure of α‐amylase (PDB ID: 4X0N) was downloaded from Protein Data Bank and subsequently analyzed to search for potential pockets using CavityPlus (http://pkumdl.cn:8000/cavityplus/).^[^ ^48^ ^]^ Additionally, the structures of all polyphenols were sketched using Sybyl 2.0 software. Then, the specific active pocket of α‐amylase including Asp^197^, Glu^233^, and Asp^300^ was preferred as the docking site with TA due to the strong competitive inhibition and the complex molecular structure of this polyphenol. Subsequently, the present docking site was utilized to obtain the binding information of RA, 5‐CSA, and CA, as well as the interactions between α‐amylase and substrates including simplified amylose, simplified amylopectin, and GalG2CNP. For inhibitors exhibiting the uncompetitive inhibition characteristic (RA, 5‐CSA, and SA), a potential pocket containing the flexible loop (Gly^304^‐His^305^‐Gly^306^‐Ala^307^‐Gly^308^‐Gly^309^) near the active pocket was selected as the target site, considering the important role of this loop in assisting the interactions between α‐amylase and the long‐chain substrate. After that, the binding energy (*E*_b_) was obtained using equation (13) as follows:^[^ ^49^ ^]^

(13) $$E_{b}=RT\log_{e}\left(10^{-pk_{d}}\right)$$

where, *p*k_d_ was the best docking score; *RT* was 0.59 kcal mol^−1^.

### Statistical Analysis

The enzymatic data were fitted using GraphPad Prism to extract the kinetic constants. The statistically difference was analyzed using t‐test and one‐way ANOVA followed by Tukey's test, which was labeled as ****(*p* < 0.0001), ***(*p* < 0.001), **(*p* < 0.01), *(*p* < 0.05) and ^ns^ (*p* > 0.05).

## Conflict of Interest

The authors declare no conflict of interest.

## Supporting information

Supporting Information

## Data Availability

The data that support the findings of this study are available from the corresponding author upon reasonable request.
