# Supplementary material for: Enzymolysis Modes Trigger Diversity in Inhibitor‐α‐Amylase Aggregating Behaviors and Activity Inhibition: A New Insight Into Enzyme Inhibition
Source: Adv Sci (Weinh). 2024 Sep 5;11(41):2404127. doi: 10.1002/advs.202404127 (PMC11538681; doi:10.1002/advs.202404127)
Supplement: Supplementary file 1 — Supporting Information [file ADVS-11-2404127-s001.docx]

**Enzymolysis Modes Trigger Diversity in Inhibitor-α-amylase Aggregating Behaviors and Activity Inhibition: A New Insight into Enzyme Inhibition**

Junwei Cao ^a^, Jifan Zhang ^a^, Ruibo Cao ^a^, Bin Zhang ^c^, Ming Miao ^b, *^, Xuebo Liu ^a^, Lijun Sun ^a, *^

^a^ College of Food Science and Engineering, Northwest A&F University, China

^b^ State Key Laboratory of Food Science and Resources, Jiangnan University, 1800 Lihu Avenue, Wuxi, Jiangsu, 214122, China

^c^ School of Food Science and Engineering, South China University of Technology, Guangzhou, 510640, China

^*^Corresponding author.

Lijun Sun, College of Food Science and Engineering, Northwest A&F University, Yangling, Shaanxi 712100, P.R. China. Email: lijunsun@nwafu.edu.cn

Ming Miao, State Key Laboratory of Food Science and Resources, 1800 Lihu Avenue, Wuxi, Jiangsu 214122, P. R. China. **Email:** miaoming@jiangnan.edu.cn


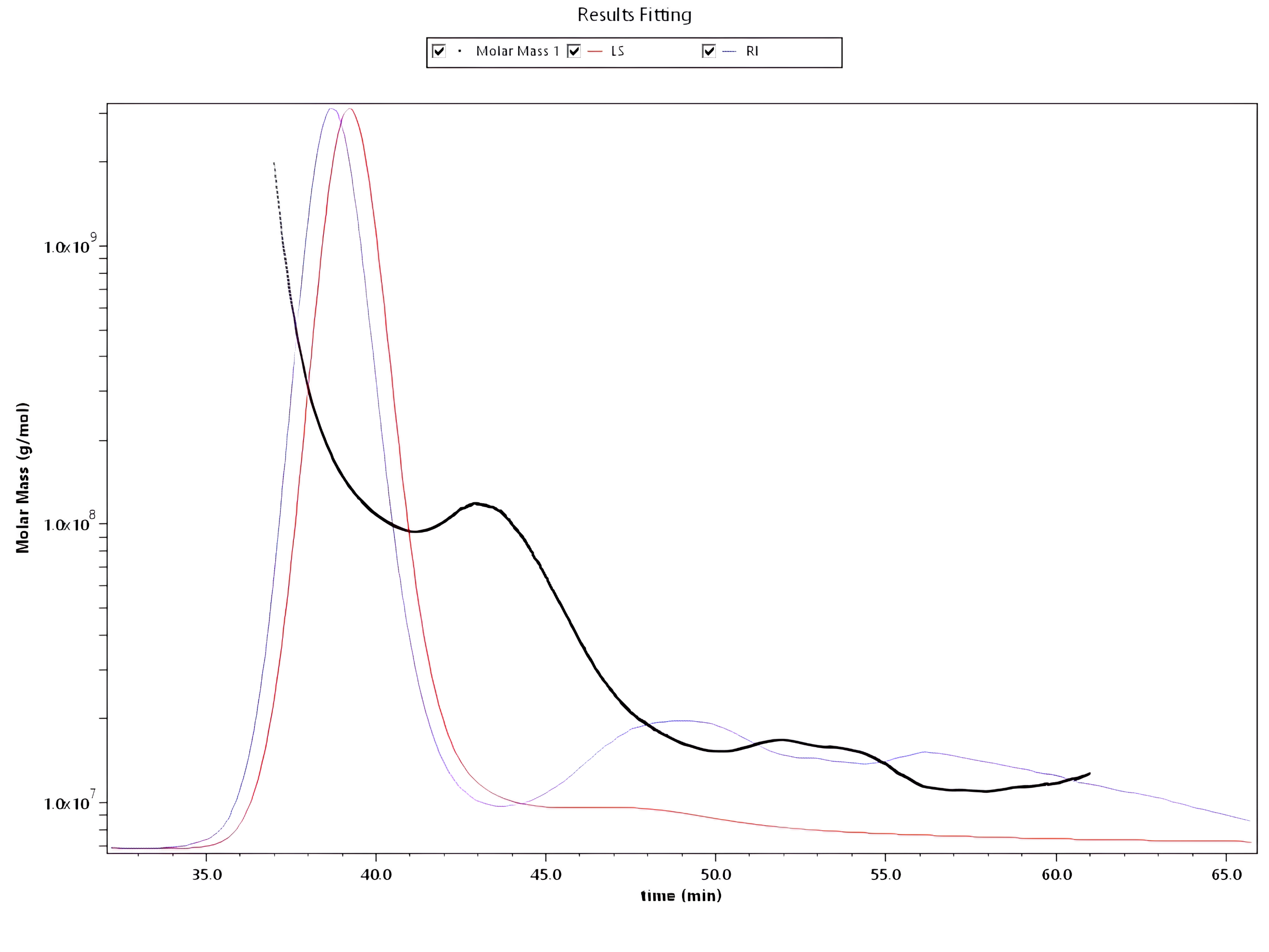


M_w_=1.63×10^8^ g/mol

**Time (min)**

**Molar mass (g/mol)**

**Figure S1**. The average molecular mass (M_w_) of NMS determined using SEC-MALLS-RI. The red line was the multi-angle laser light scattering signal, reflecting the sample size; the blue line represented the differential signal, indicating the sample concentration; and the black line denoted the molecular weight fitted from the combination of the above two signals.

**Figure S2.** Characterization of the chain length distribution of NMS using gel permeation chromatography (GPC). Peaks 1 and 2 represented the distribution of short and long amylopectin chain (DP < 100), and peak 3 provided the information on the distribution of amylose (DP > 100).

Peak 1 (61.13%)

Peak 2 (13.93%)

Peak 3 (24.94%)

**Figure S3.** Comparison of the kinetic constants in GalG2CNP (**a**) and starch (**b**) digestion systems including the maximal initial reaction velocity (*V*) and Michaelis constant (*K*_m_) values calculated from the Michaelis-Menten, Lineweaver-Burk and Hanes-Woolf equations.

**(a)**

**GalG2CNP**

**(b)**

**Starch**

**Figure S4.** The inhibition effects of polyphenols (TA and SA) in the absence and presence of Triton X-100, which were characterized by determining the initial velocity of starch digestion.

With 0.1% Triton X-100

Without 0.1% Triton X-100

Inhibitor

concentration

Substrate

concentration

**Cornish-Bowden (SA)**

**Lineweaver-Burk (SA)**

**Slope=*K*_m_/*V***

**(-*K*_iu_, *K*_m_/*V*)**

Substrate

concentration


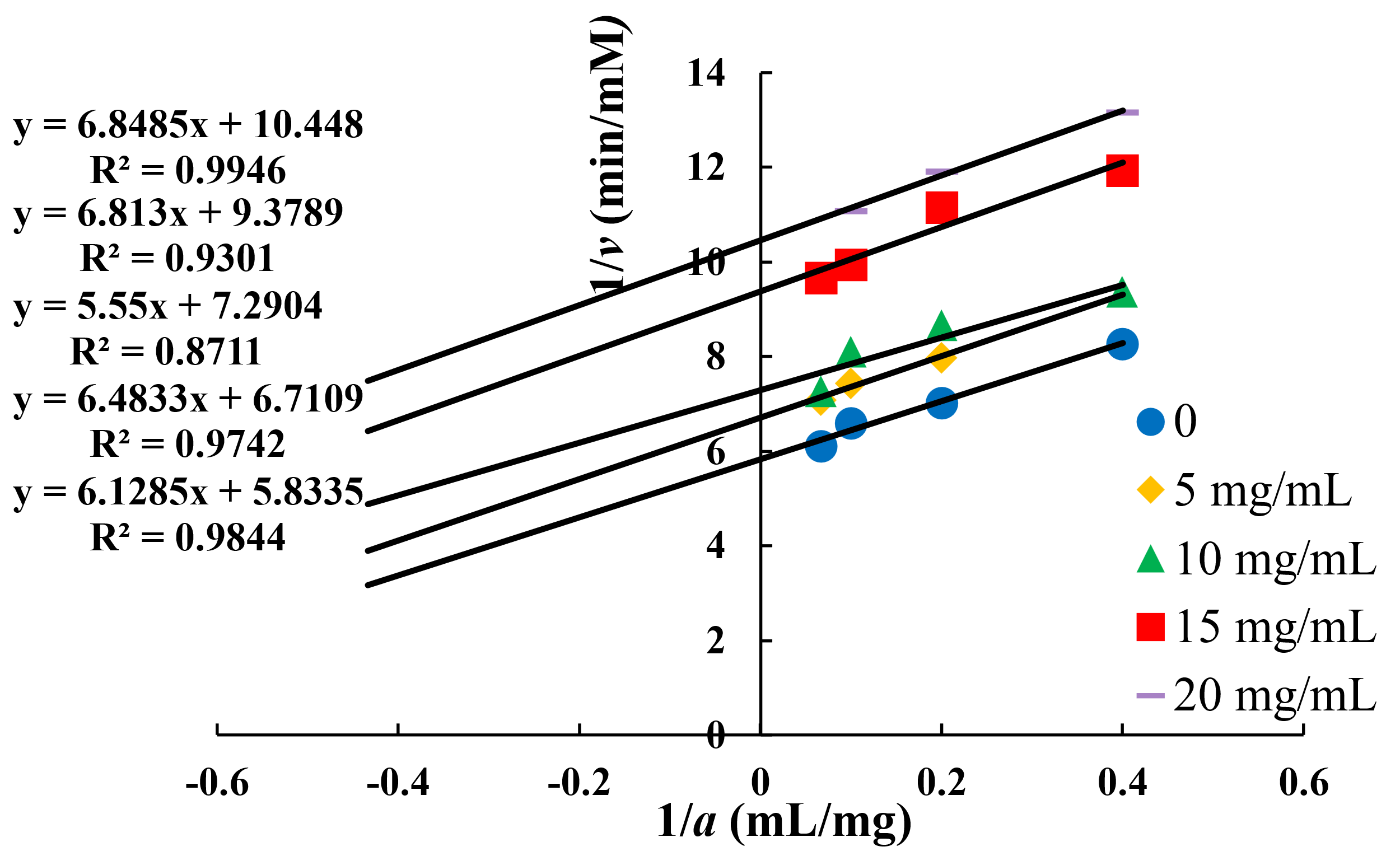

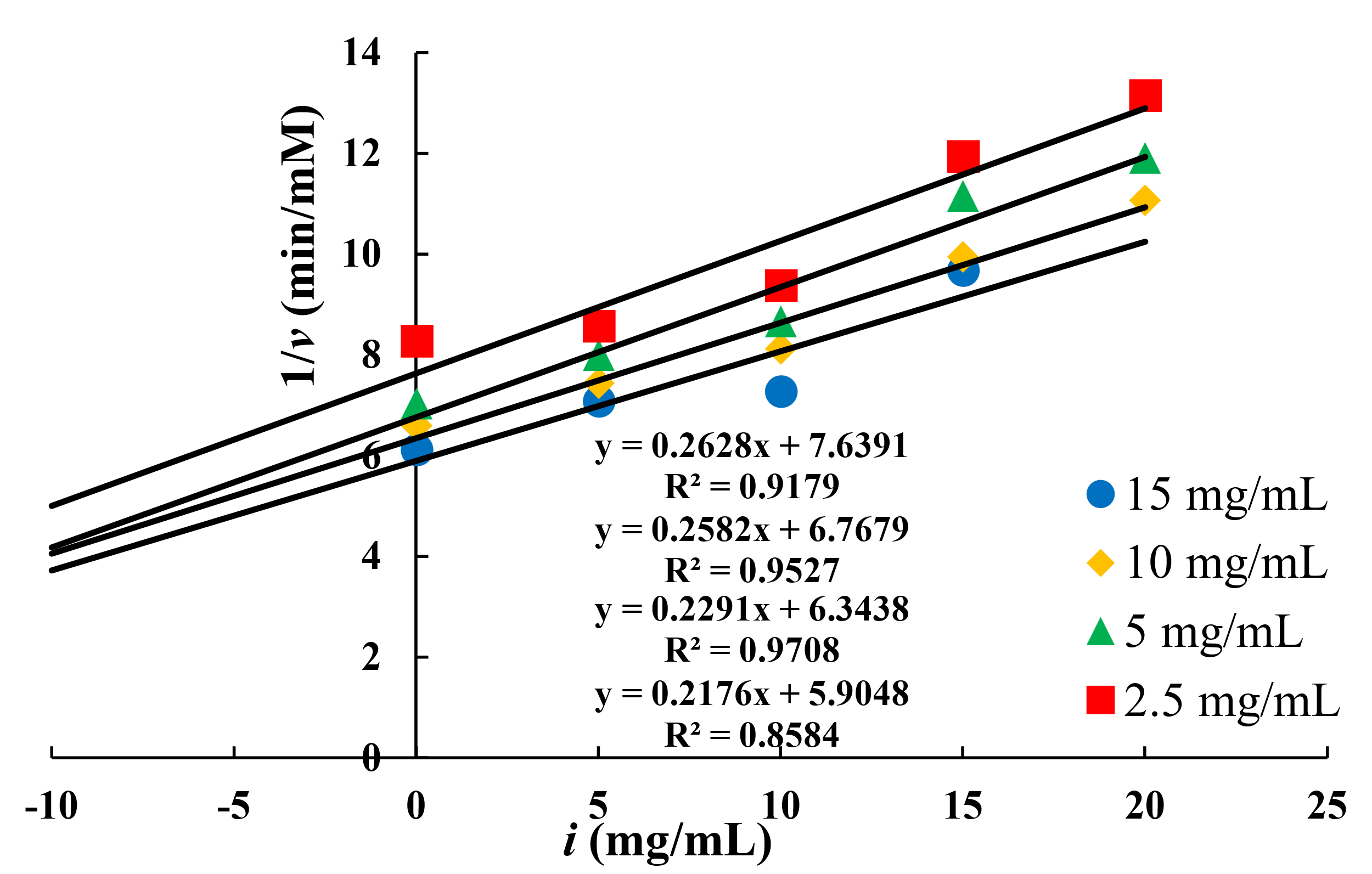

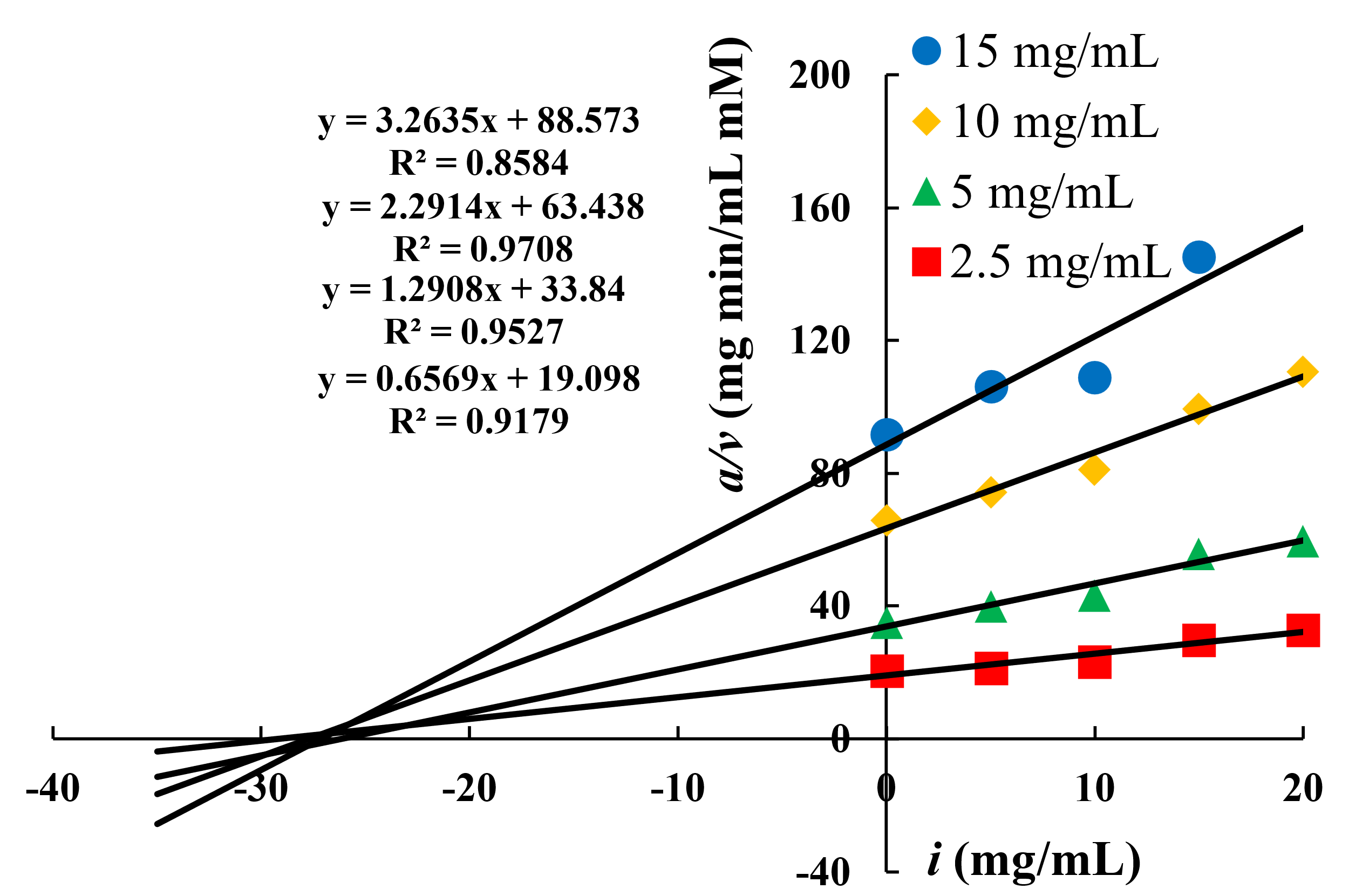


**Starch**

**Dixon (SA)**

**(f)**

**Lineweaver-Burk (5-CSA)**

Inhibitor concentration

**-*K*_iu_**

**Cornish-Bowden (5-CSA)**

Substrate

concentration

**-*K*_ic_**


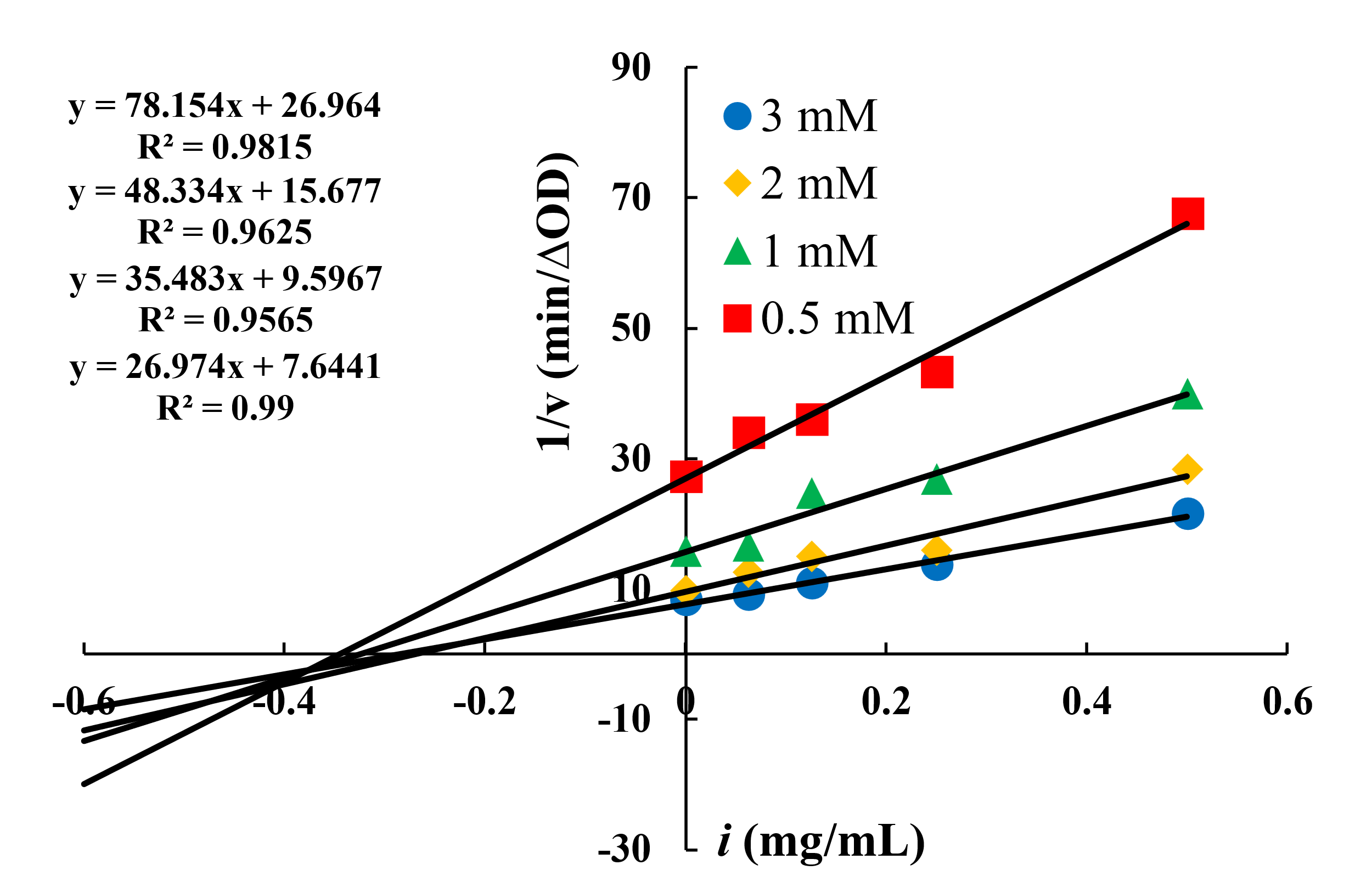

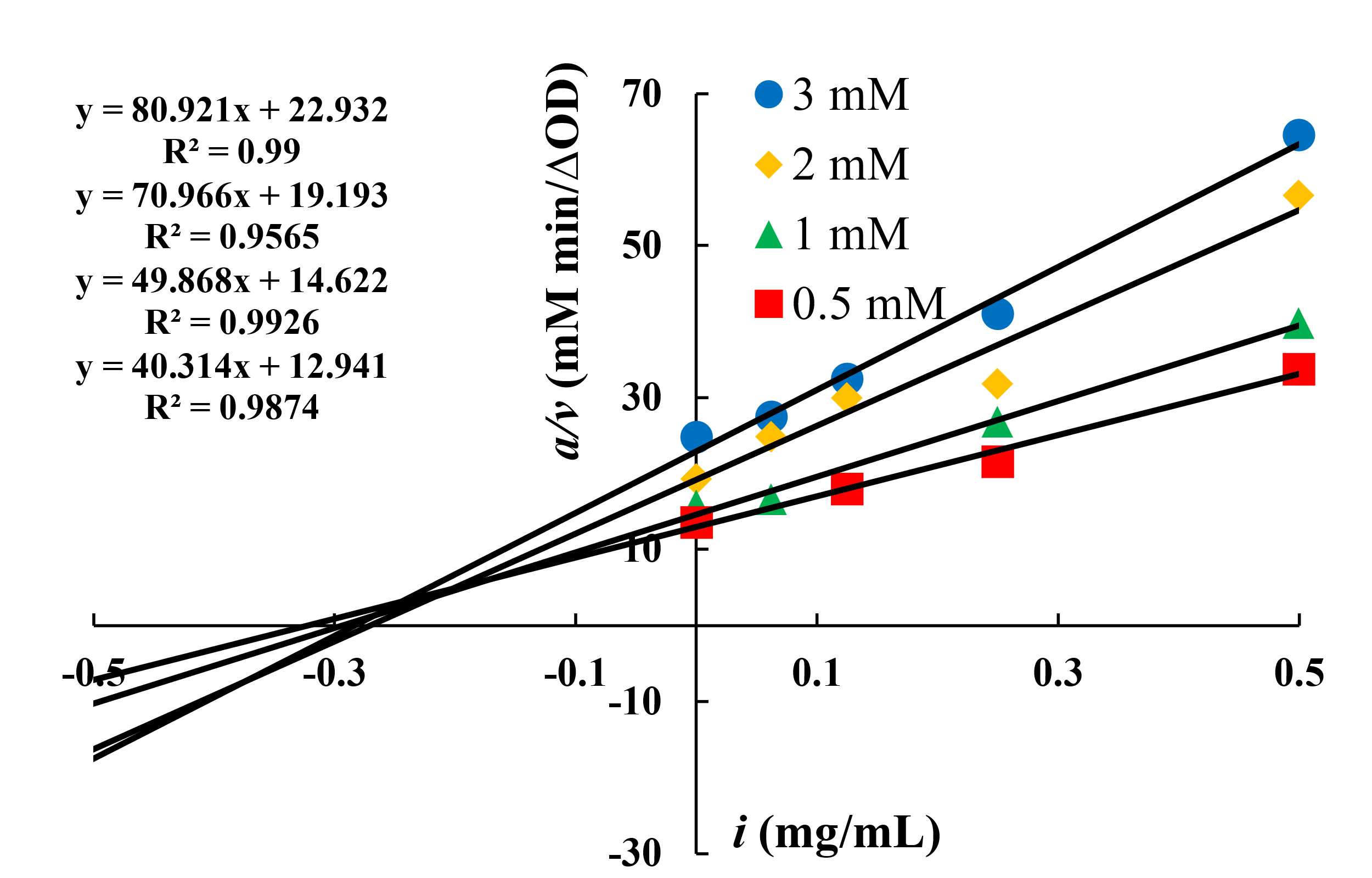

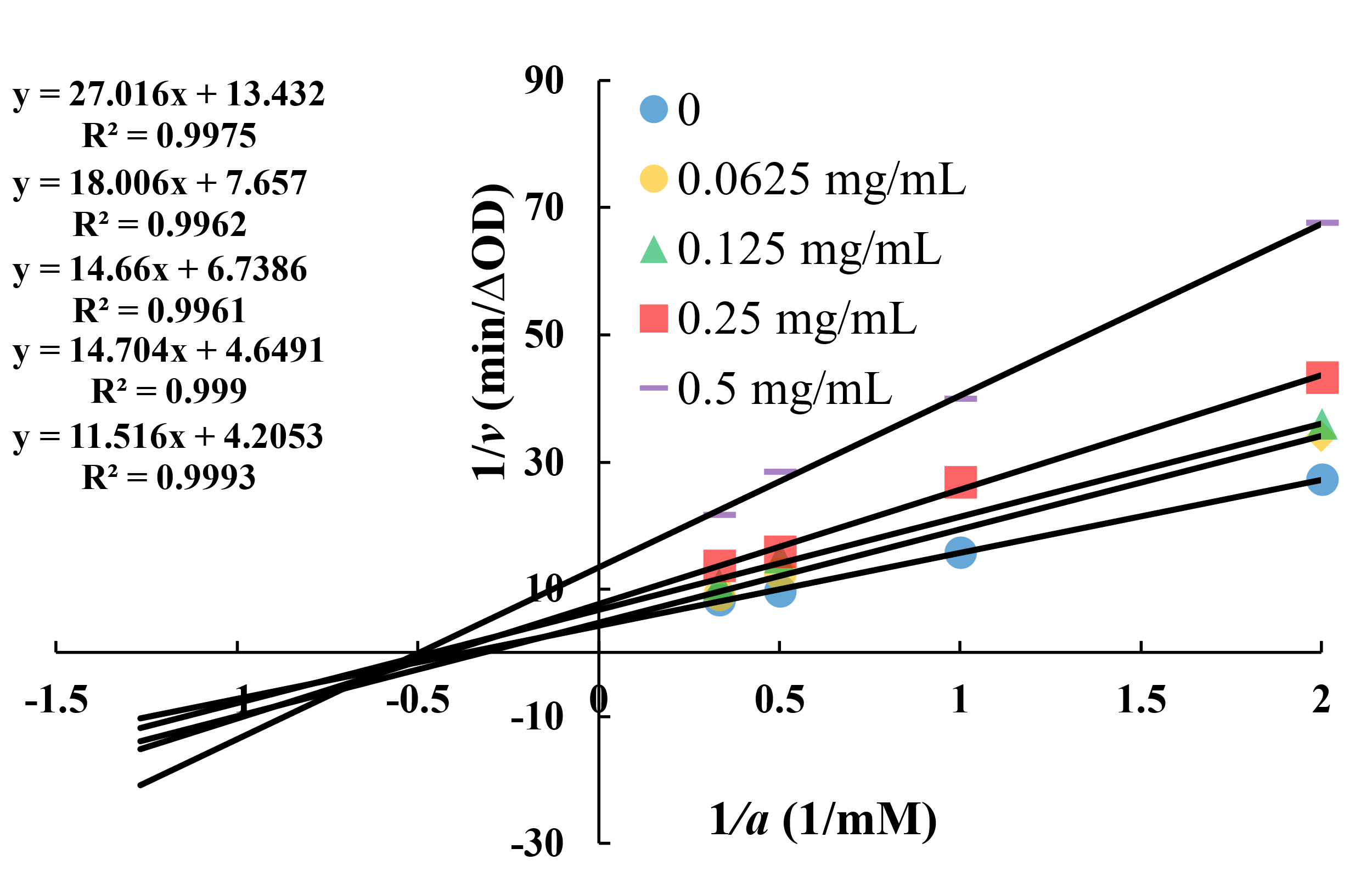


**GalG2CNP**

**Dixon (5-CSA)**

Substrate

concentration

**(c)**

**1/*V***

**Lineweaver-Burk (TA)**

Inhibitor concentration

**Cornish-Bowden (TA)**

Substrate

concentration


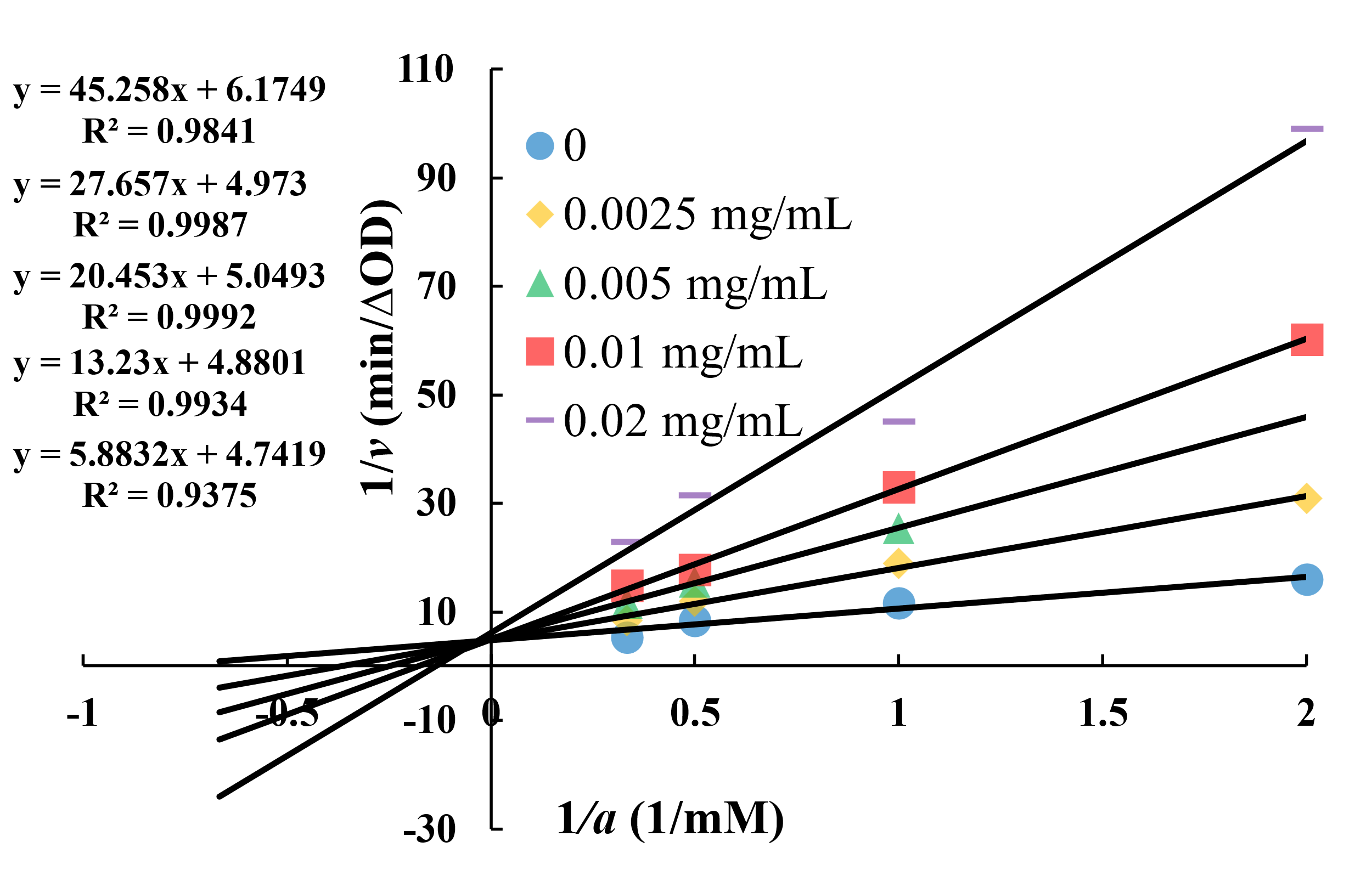

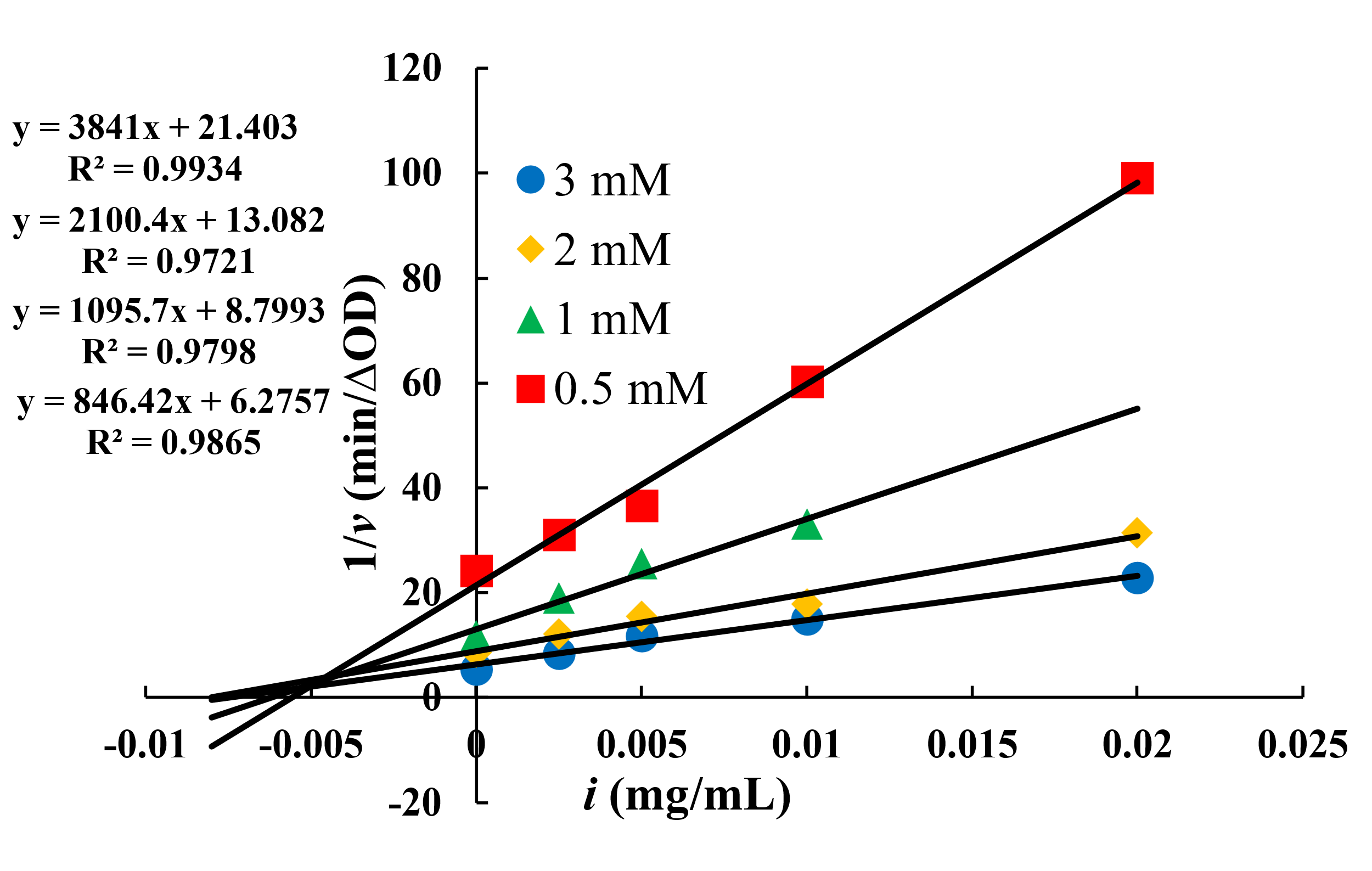

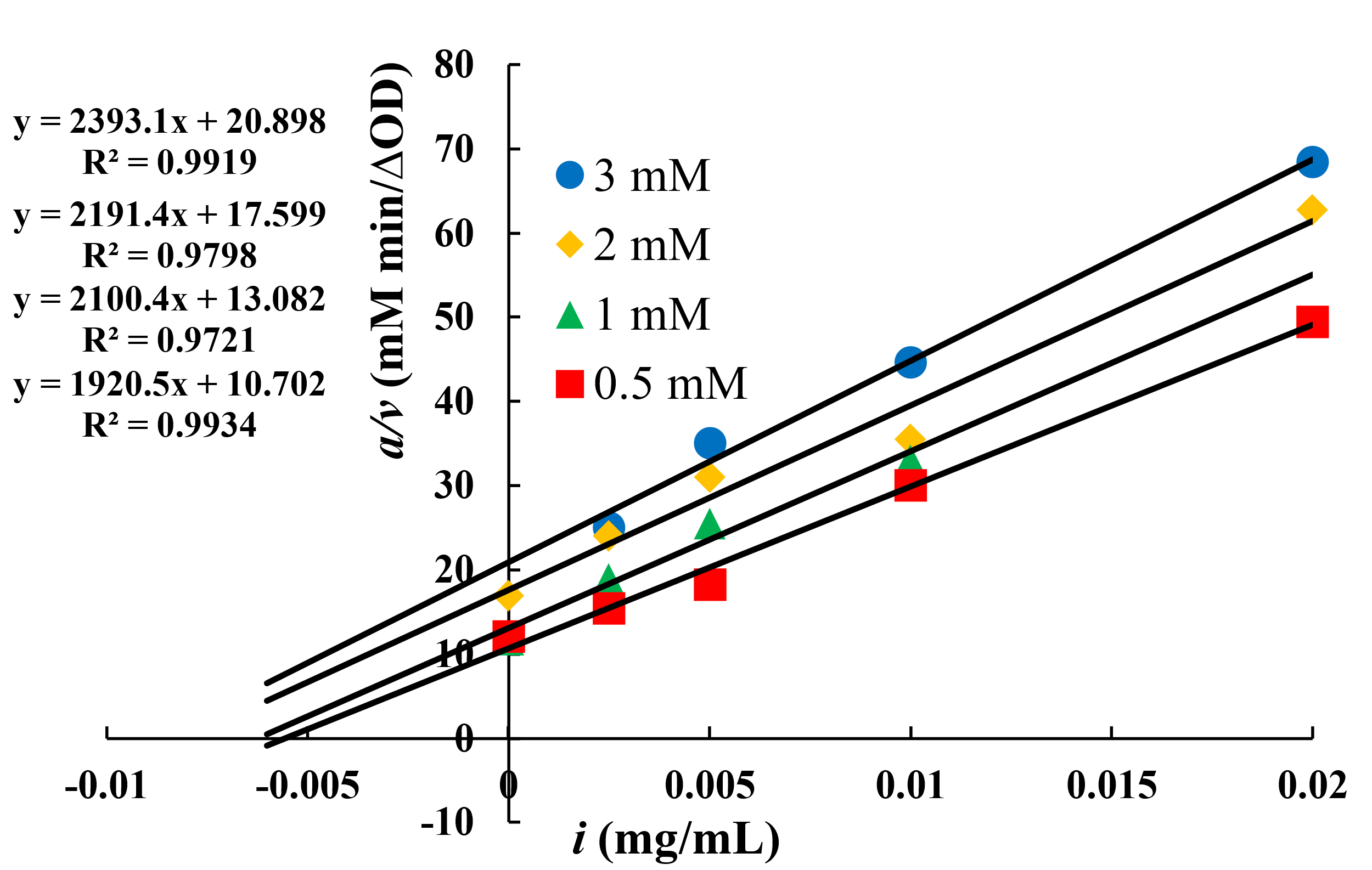


**GalG2CNP**

**Dixon (TA)**

(**-*K*_ic_,1/*V***)

Substrate

concentration

**(a)**

**Lineweaver-Burk (RA)**

Inhibitor concentration

**-*K*_iu_**

Substrate

concentration

**Cornish-Bowden (RA)**


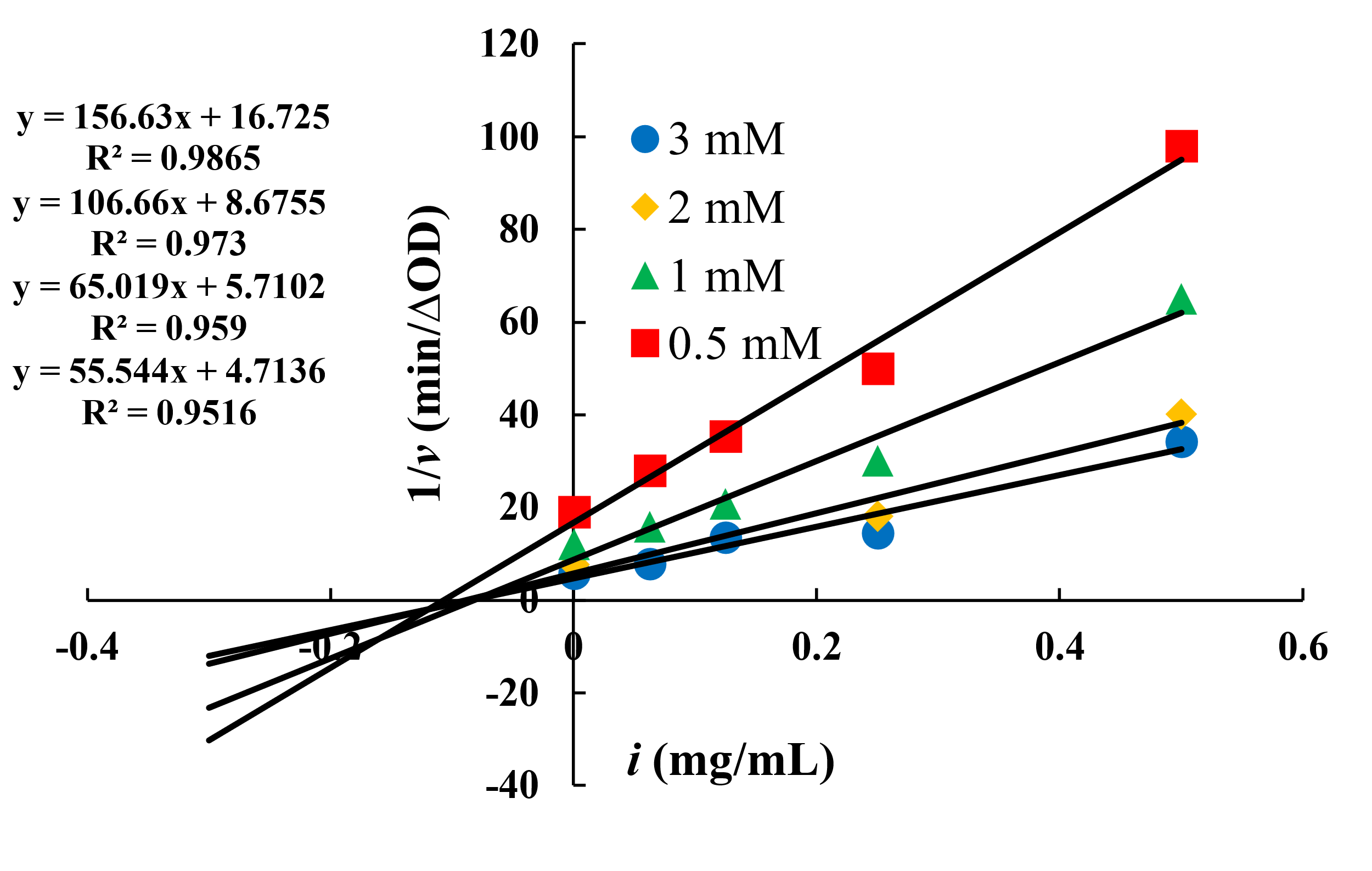

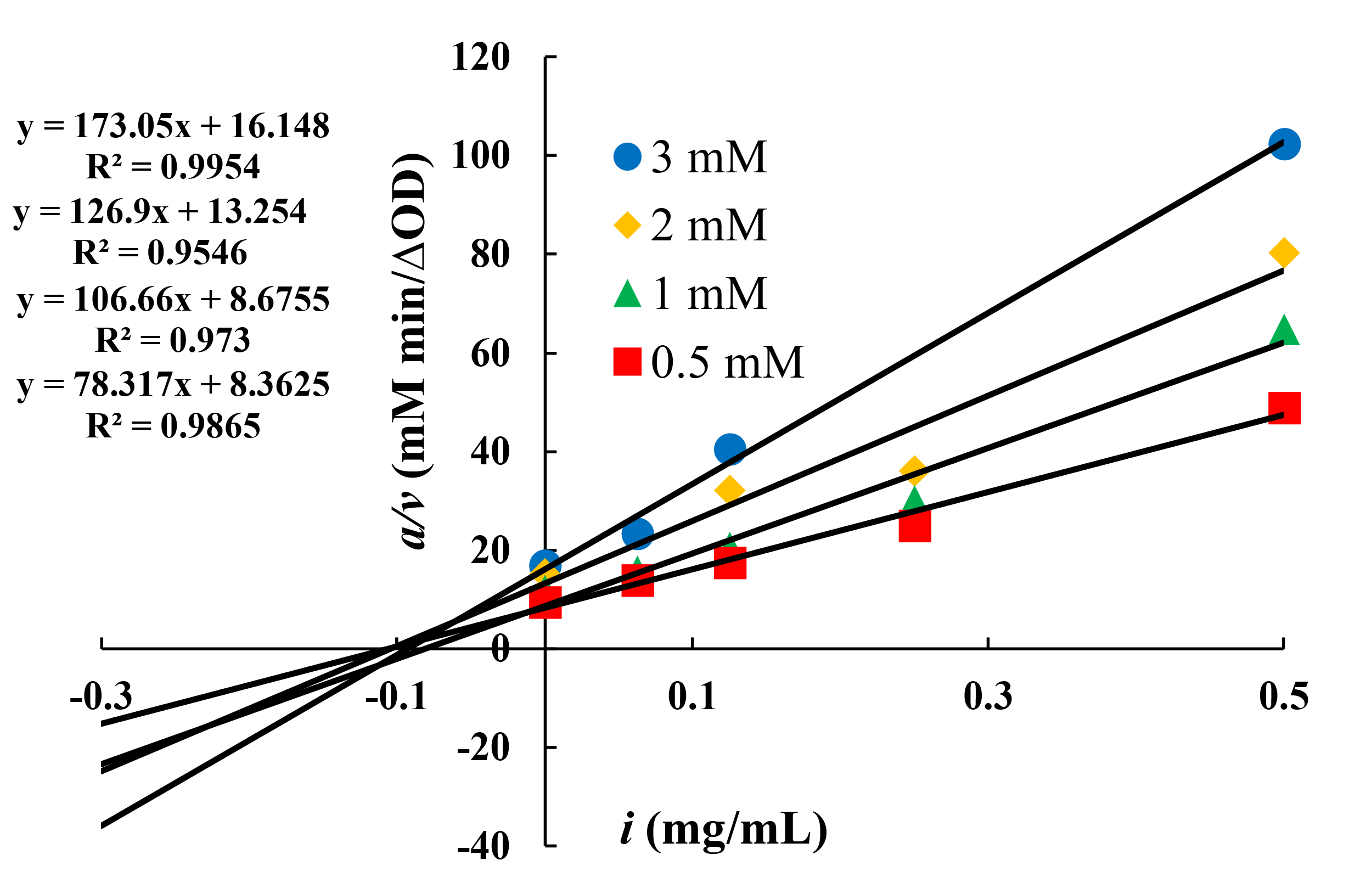

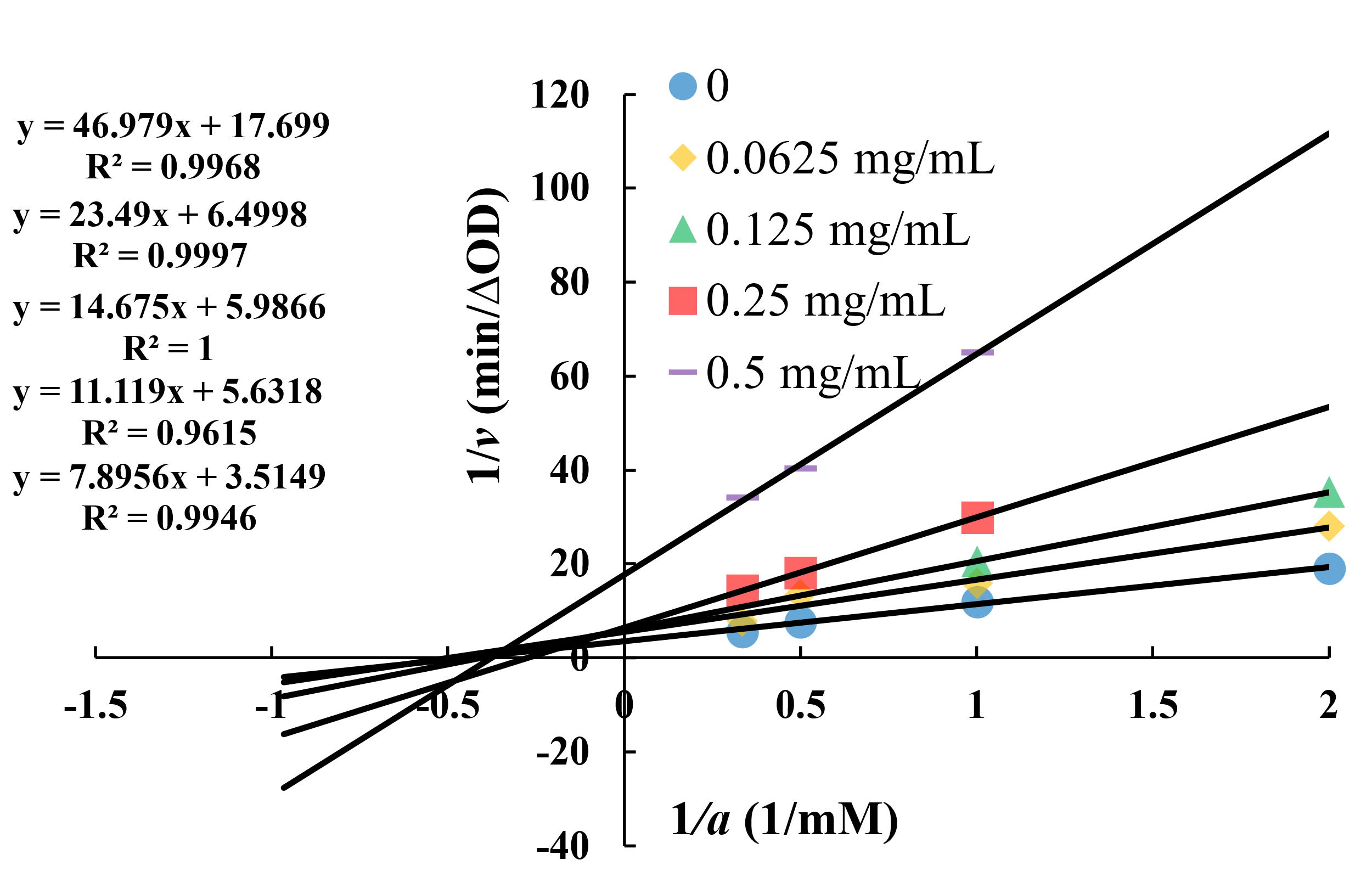


**GalG2CNP**

**Dixon (RA)**

Substrate

concentration

**-*K*_ic_**

**(b)**

**Starch**

**Dixon (TA)**

**Cornish-Bowden (TA)**

Substrate

concentration

Inhibitor concentration

**Lineweaver-Burk (TA)**

**1/*V***

(**-*K*_ic_,1/*V***)

Substrate

concentration

**(d)**


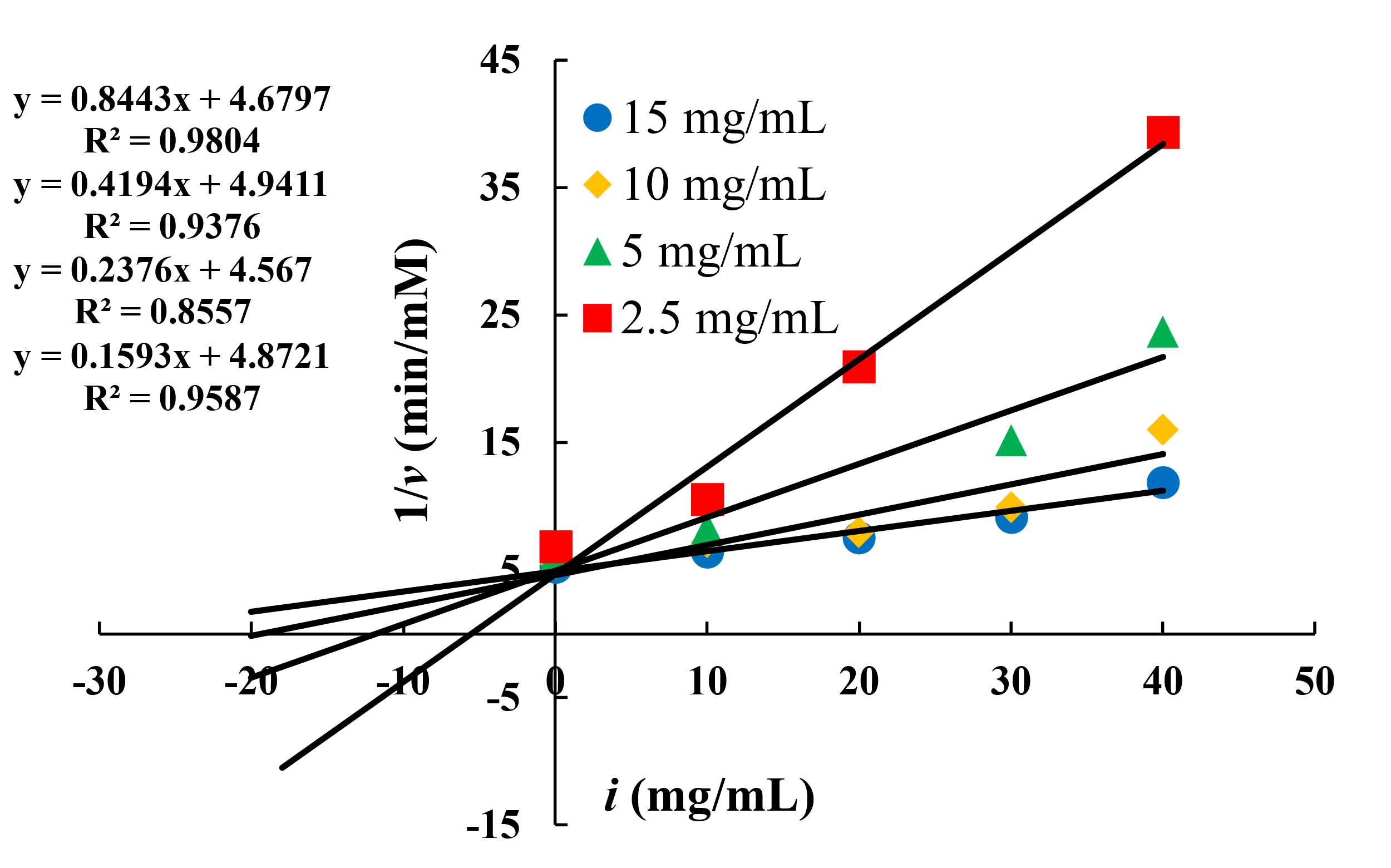

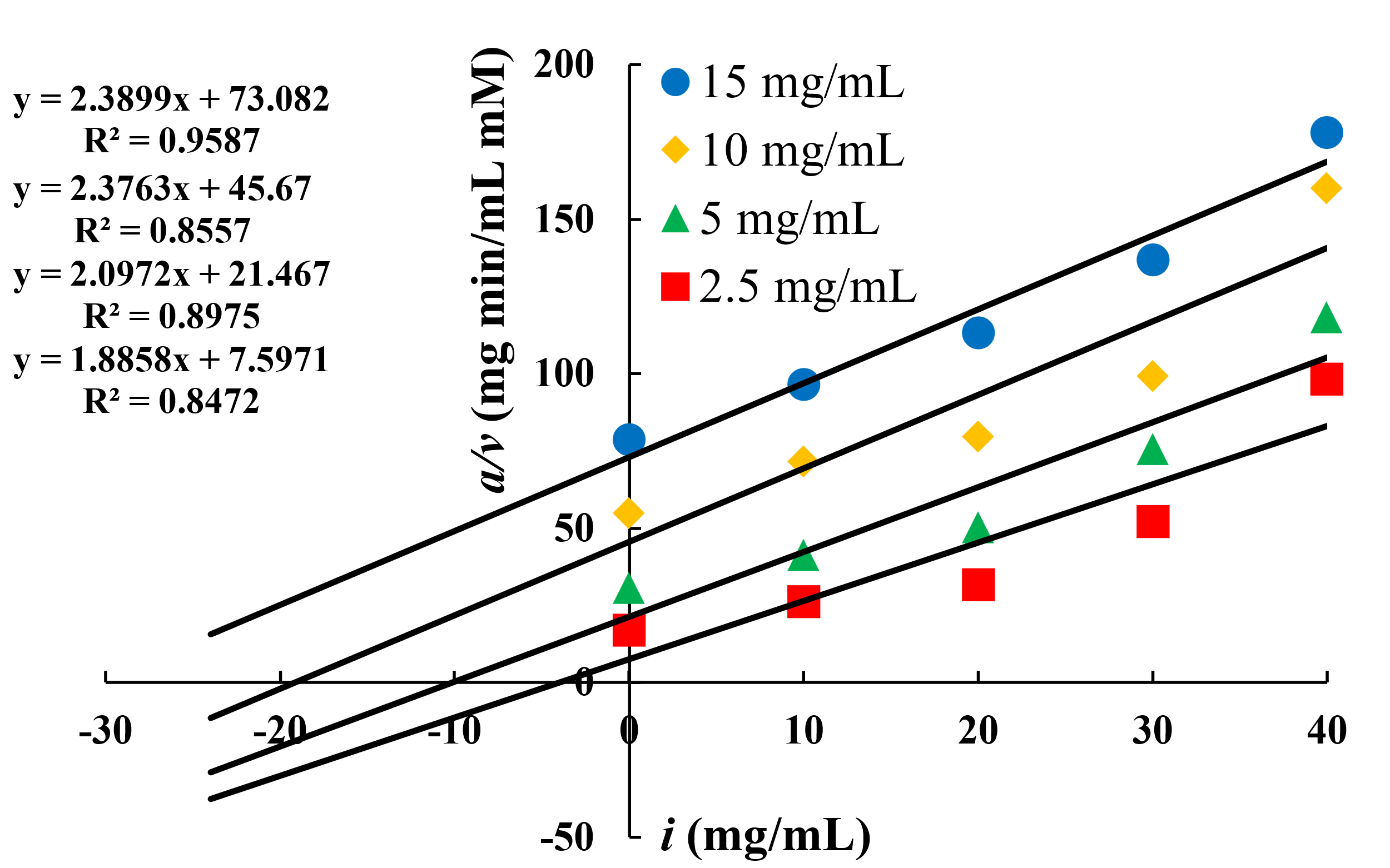

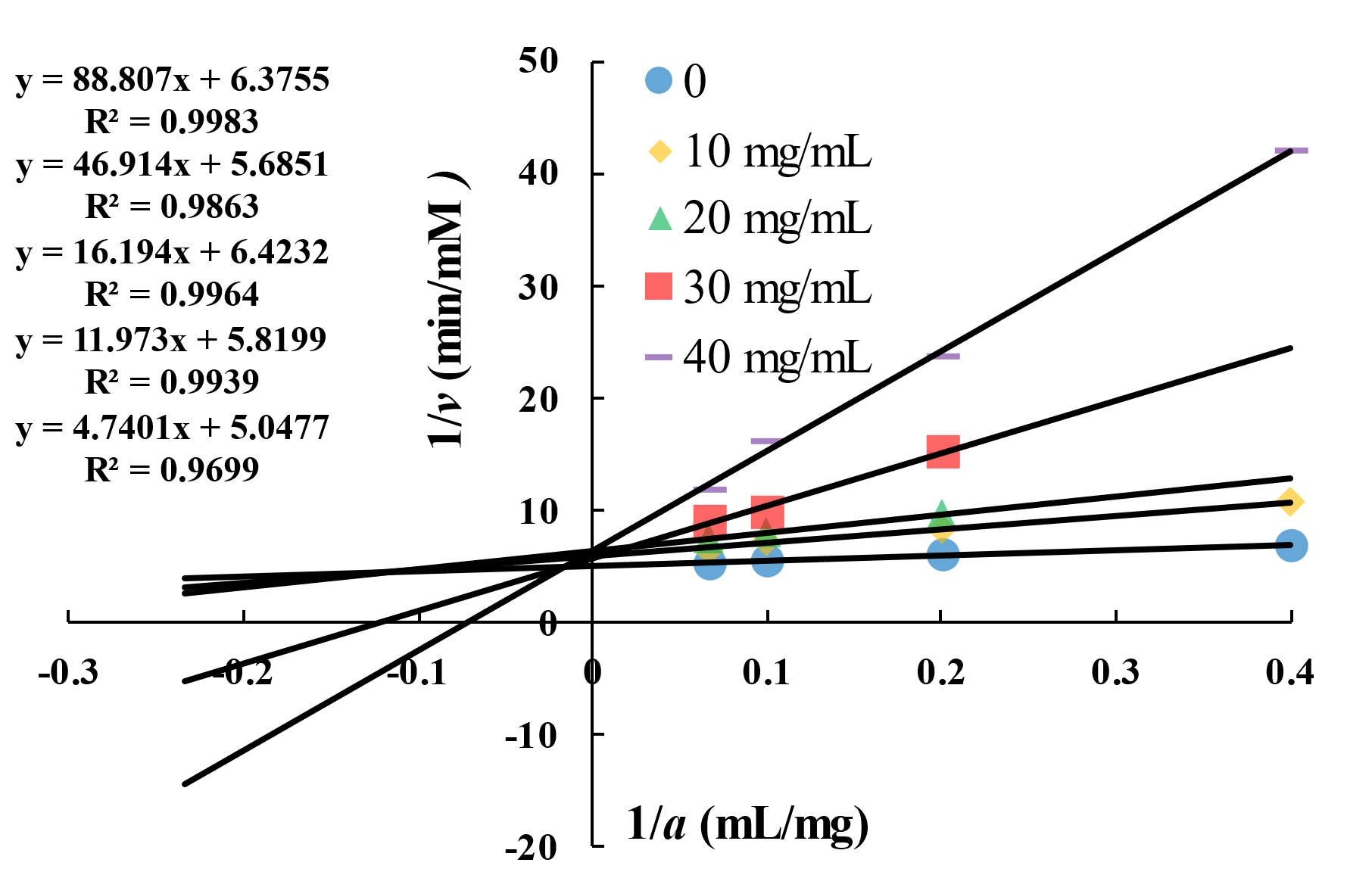


better linear curve due to plot fitting for each *i*

Inhibitor concentration

**Lineweaver-Burk (RA)**

**Cornish-Bowden (RA)**

**-*K*_iu_**

sharp increase

**-*K*_ic_**

**(e)**


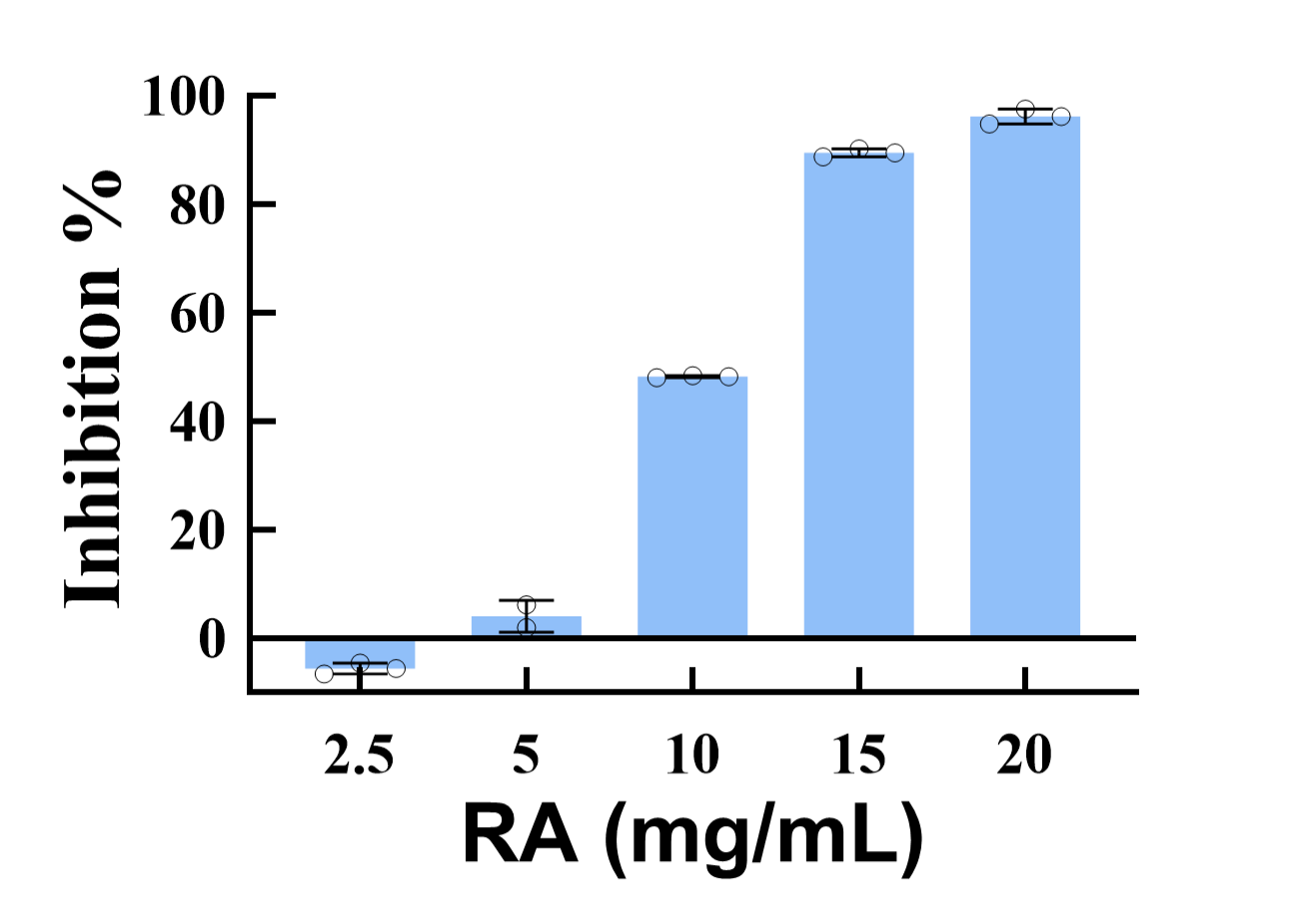

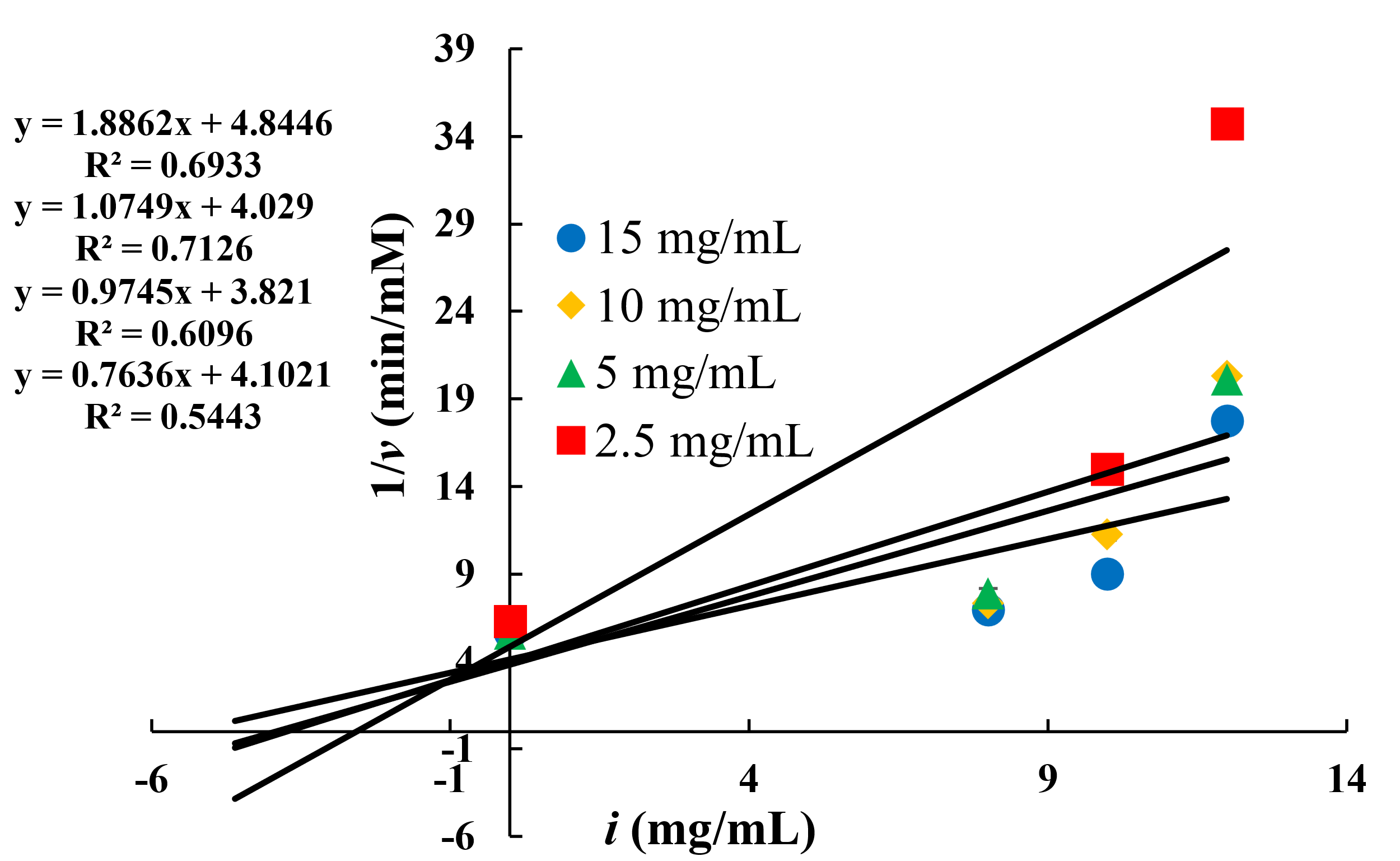

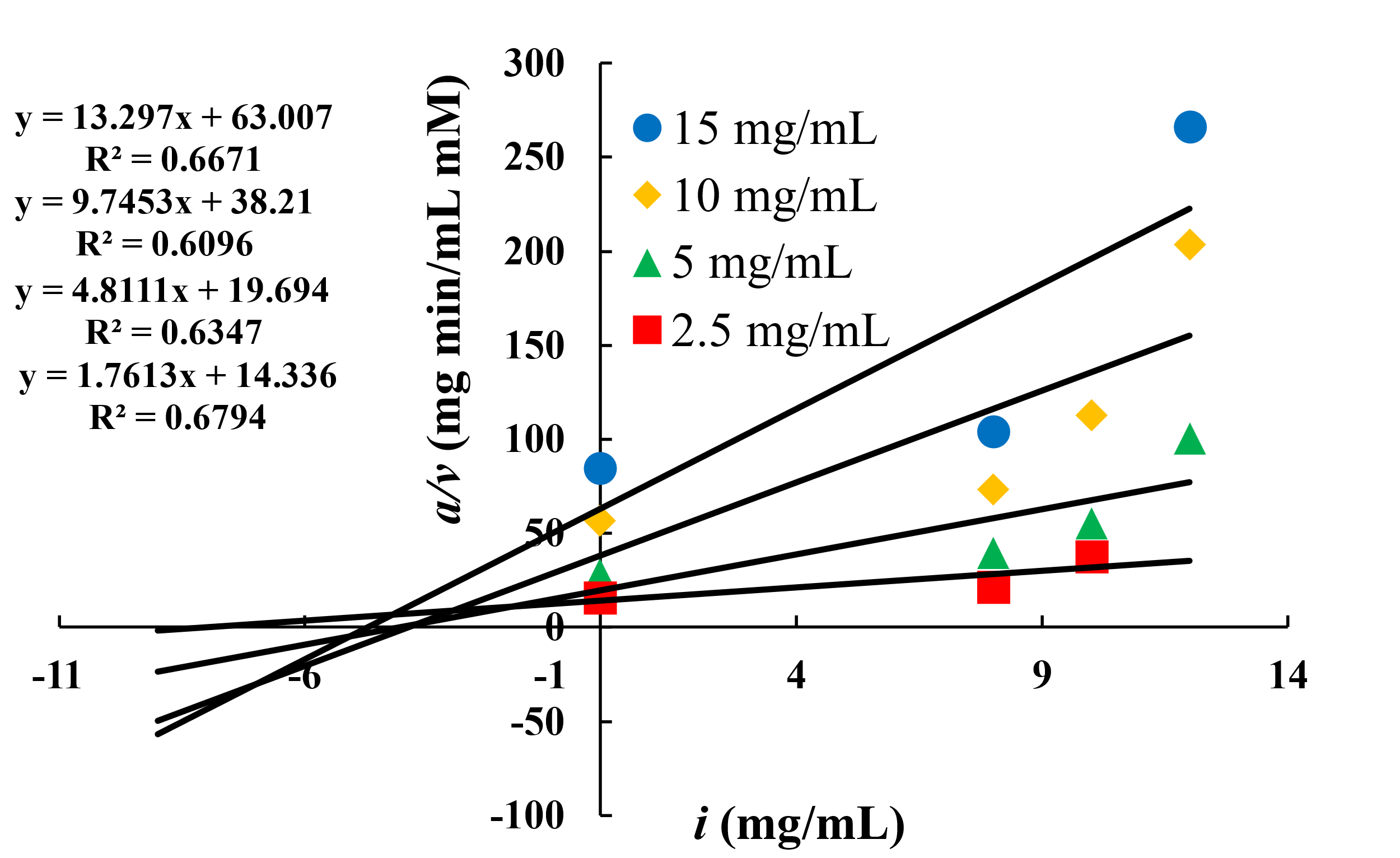

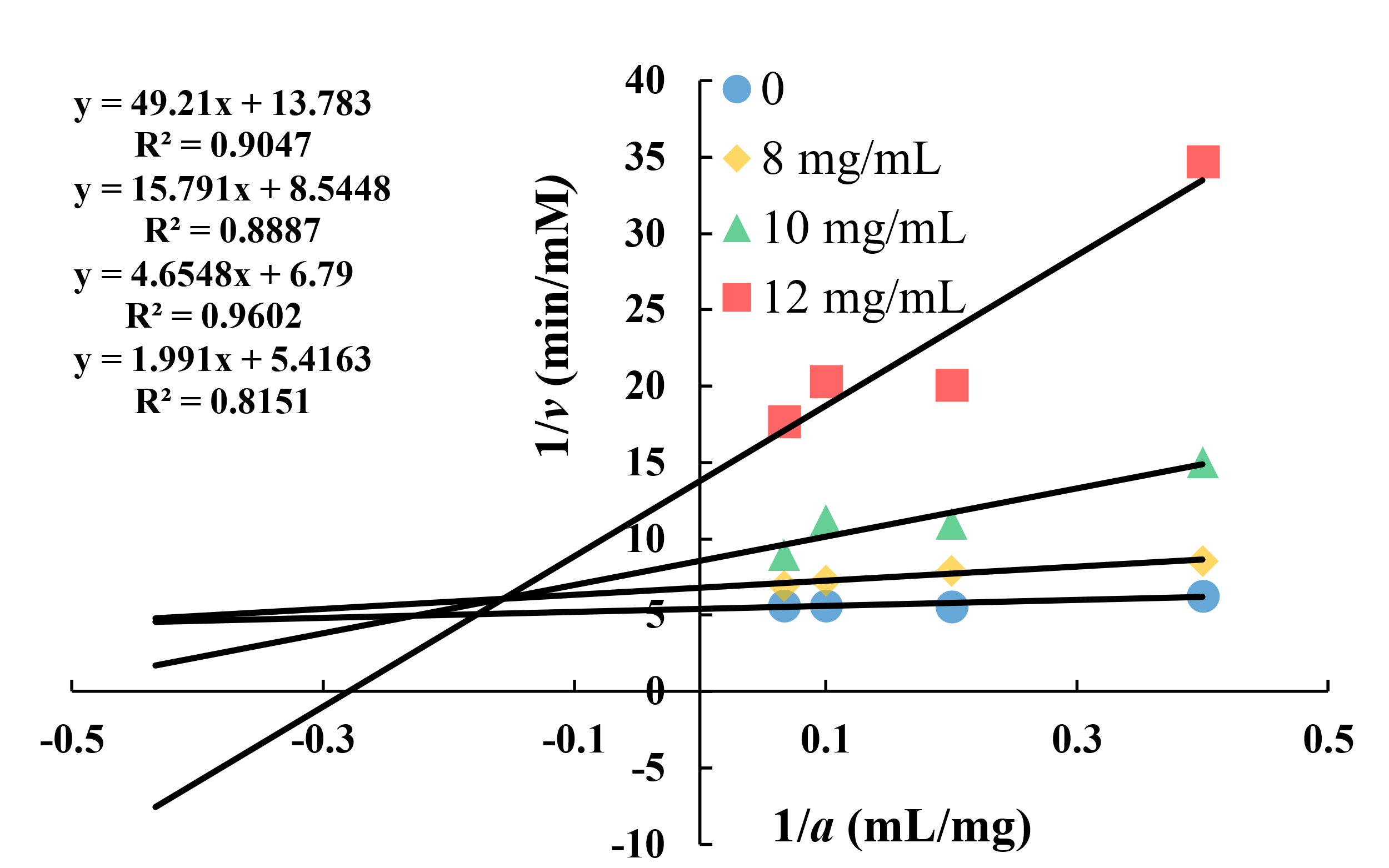


**Starch**

**Dixon (RA)**

Substrate

concentration

**Figure S5.** Inhibition kinetics of polyphenols in two digestion systems using the Dixon, Cornish-Bowden and Lineweaver–Burk equations. In detail, the kinetics analysis in GalG2CNP hydrolyzing system was applied for TA (**a**), RA (**b**) and 5-CSA (**c**), and that in NMS hydrolyzing system was utilized for TA (**d**), RA (**e**) and SA (**f**). The relatively poor linearity in the Dixon and Cornish-Bowden plots of RA in NMS hydrolyzing system resulted from the sharply increasing in enzyme inhibition due to the acidity at the high *i* ranges. However, the Lineweaver-Burk plot of RA was fitted well because the plot processes the correlation between 1/*v* and 1/*a* for each *i* individually. Besides, the competitive inhibition constant (*K*_ic_, mg/mL) and the uncompetitive inhibition constant (*K*_iu_, mg/mL) were obtained from the absolute value of abscissa of intersection in Dixon and Cornish-Bowden plots, respectively. The reciprocal of *V* (1/*V*) was obtained from the intersection of the Lineweaver-Burk plot, and the value of *K*_m_/*V* was obtained from the slope of this plot.


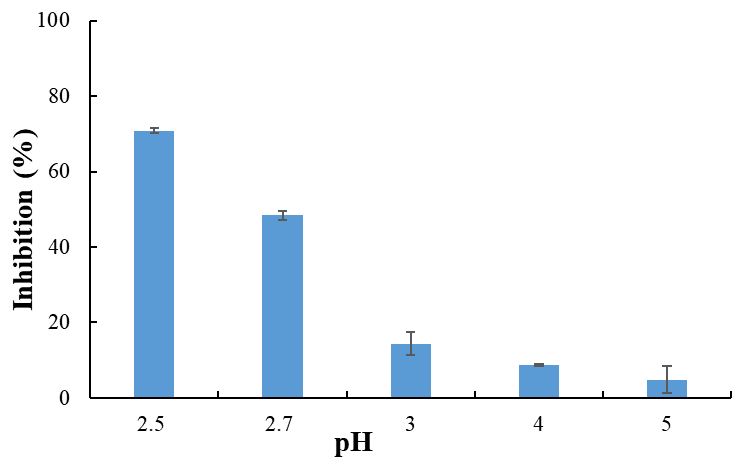


**Figure S6.** By performing the same initial velocity measurement in starch digestion system, the inhibition effects were obtained for a series of PBS-citric acid solutions with different pH values (ranging from 2.5-5.0), which were prepared using 20 mg/mL citric acid (pH 2.15).


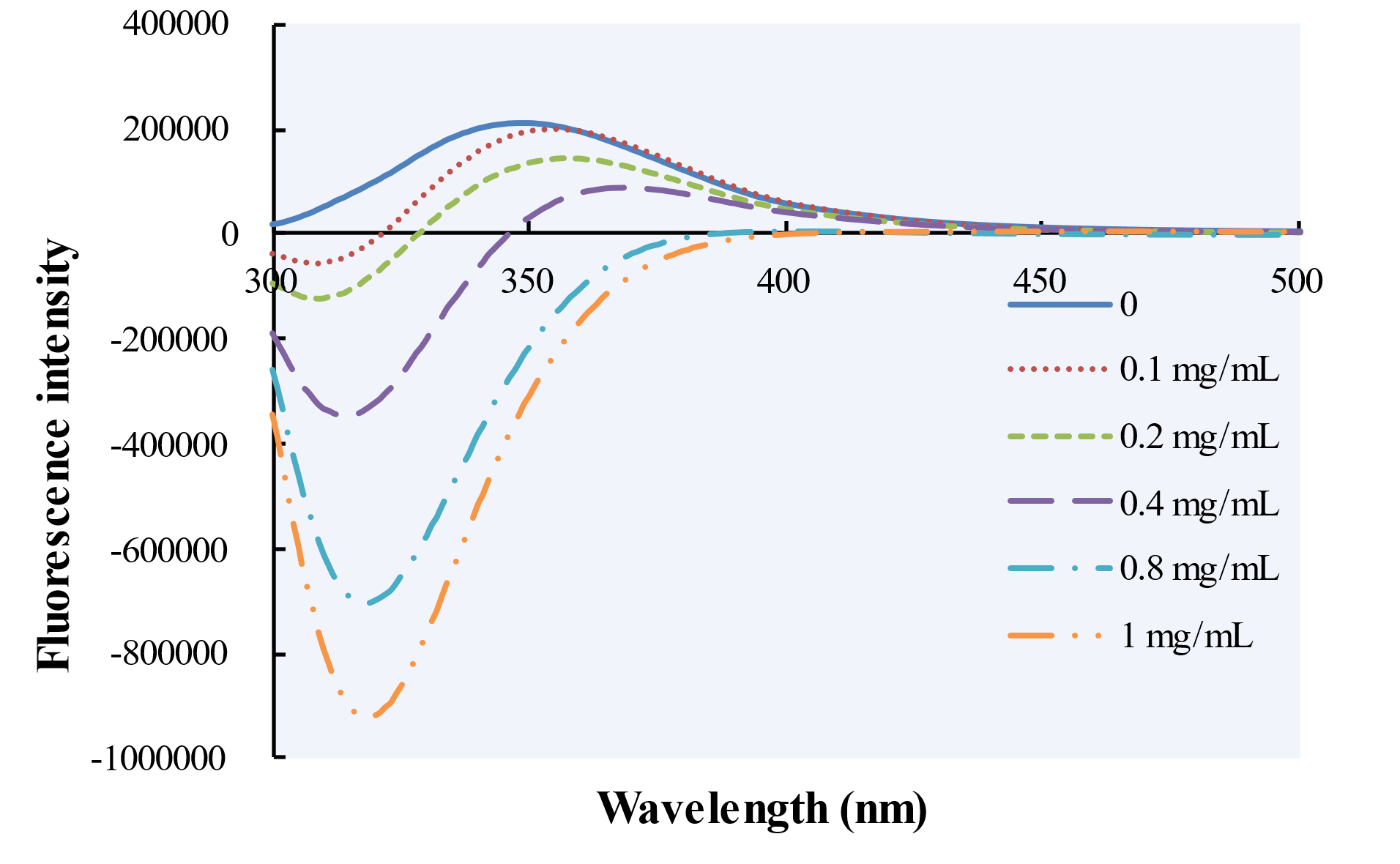

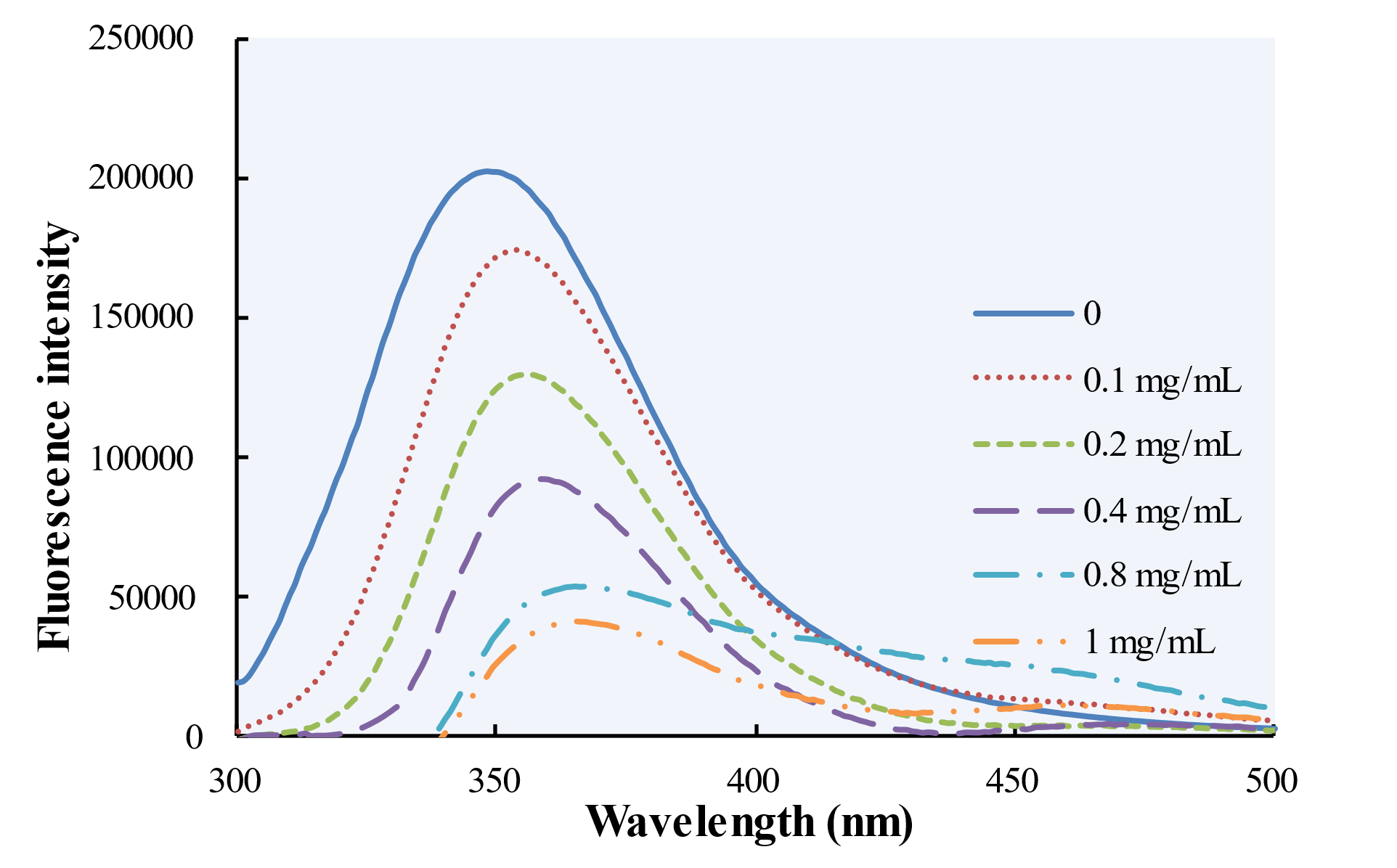

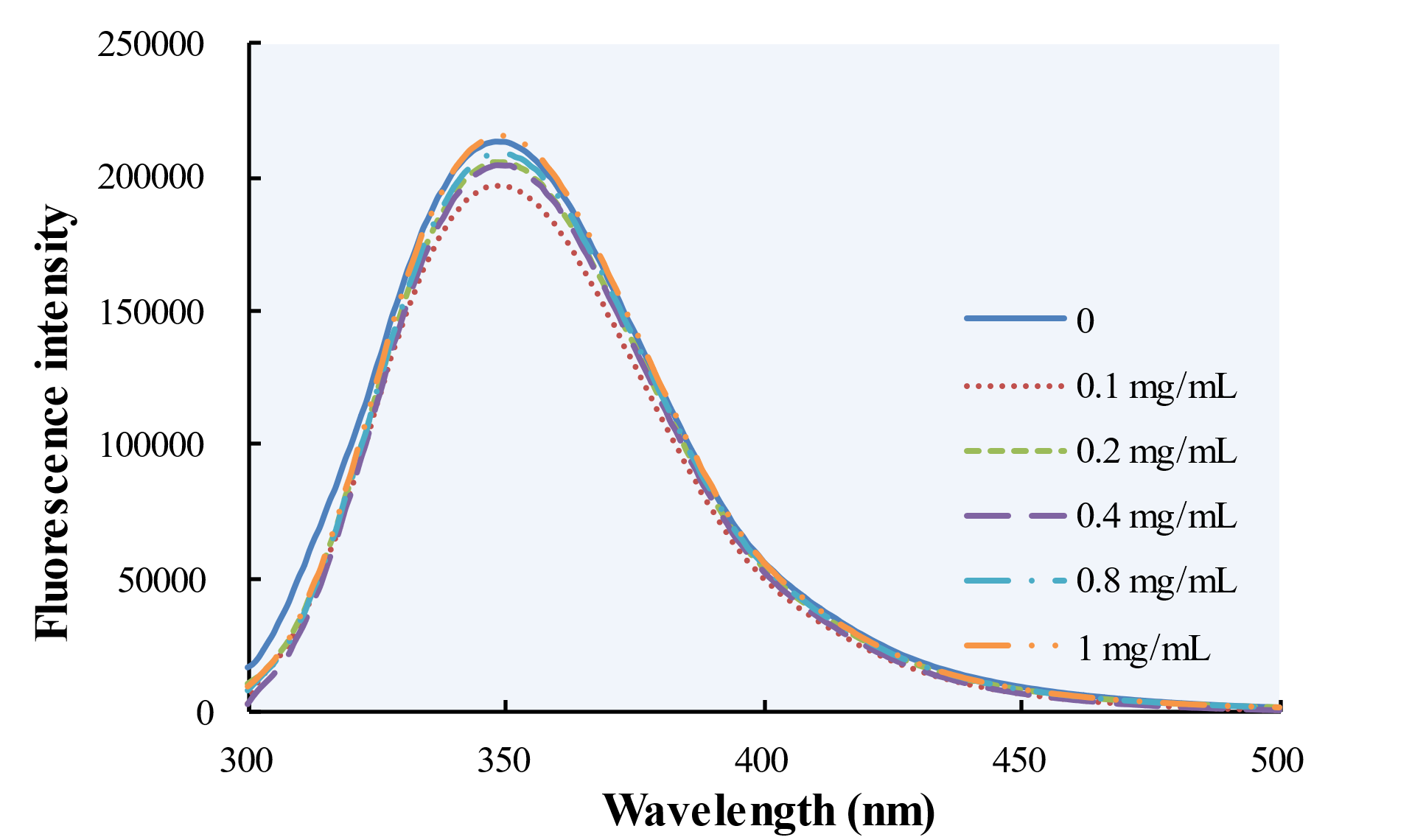

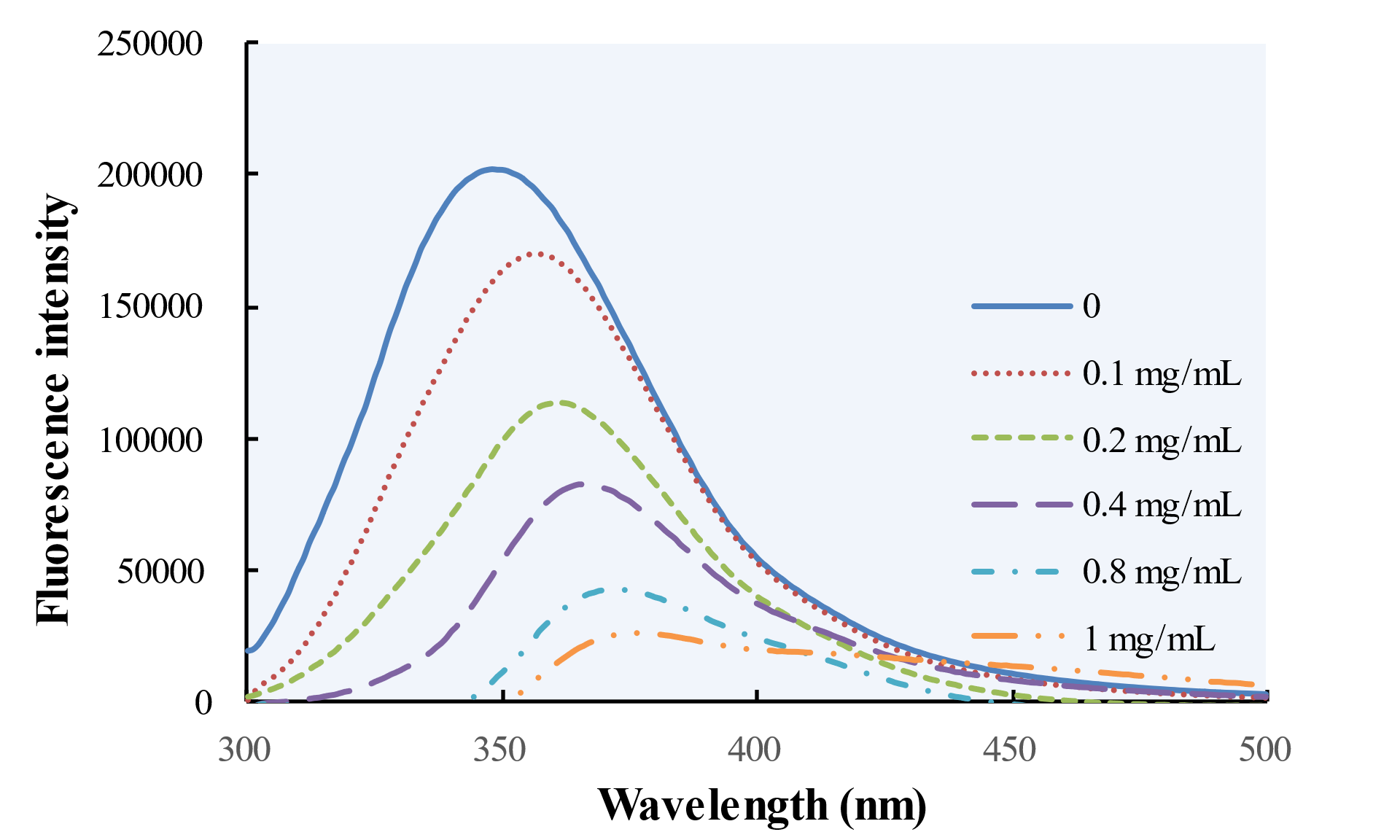

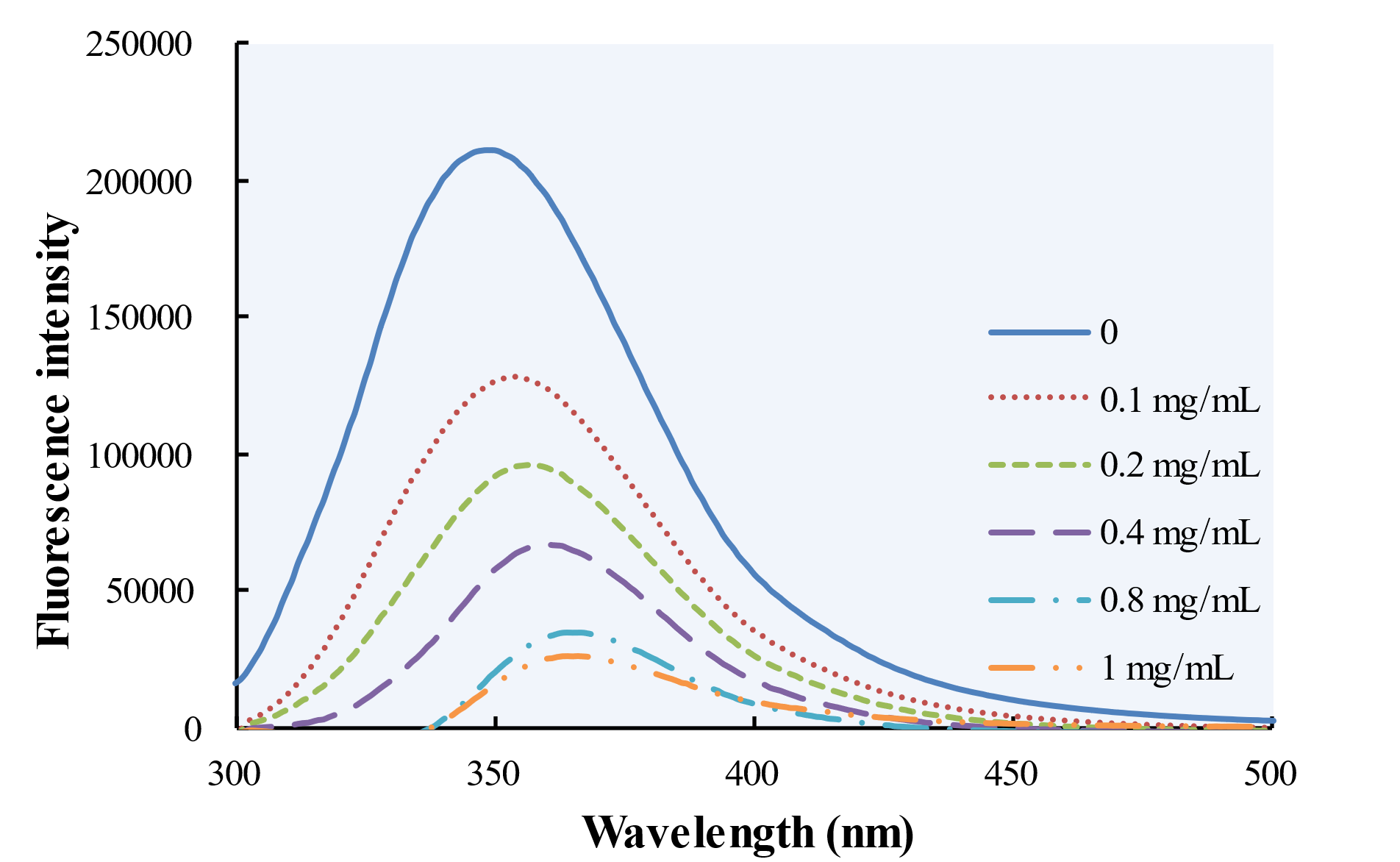


**α-amylase +TA**

**TA**

**concentration**

**(a)**


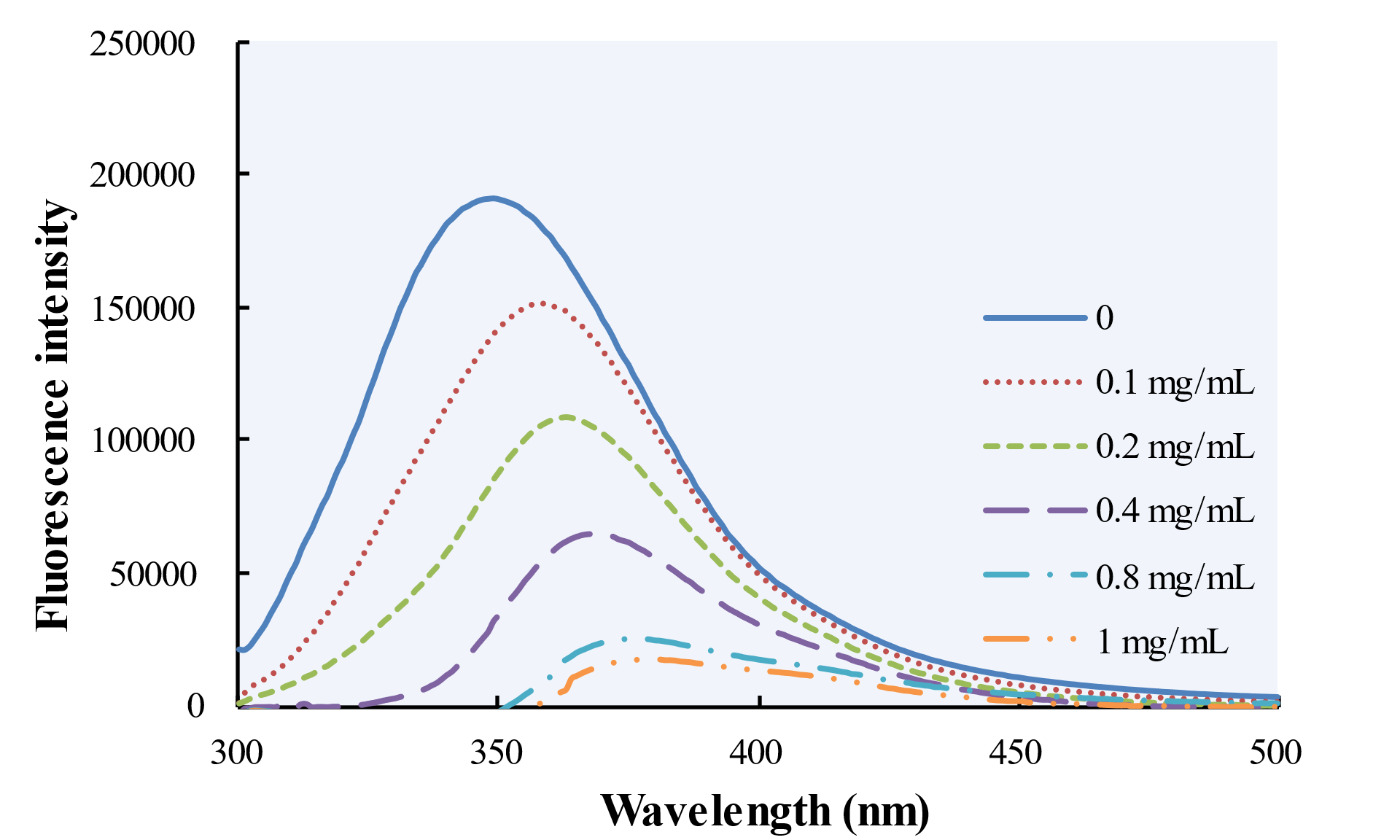


**α-amylase +5-CSA**

**5-CSA**

**concentration**

**(c)**

**α-amylase +SA**

**SA**

**concentration**

**(d)**

**α-amylase +D**

**D concentration**

**(e)**

**α-amylase +CA**

**CA**

**concentration**

**(f)**

**α-amylase +RA**

**RA**

**concentration**

**(b)**


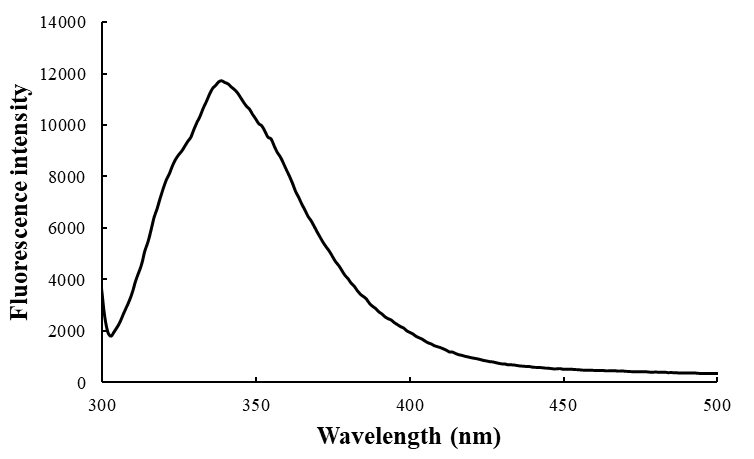

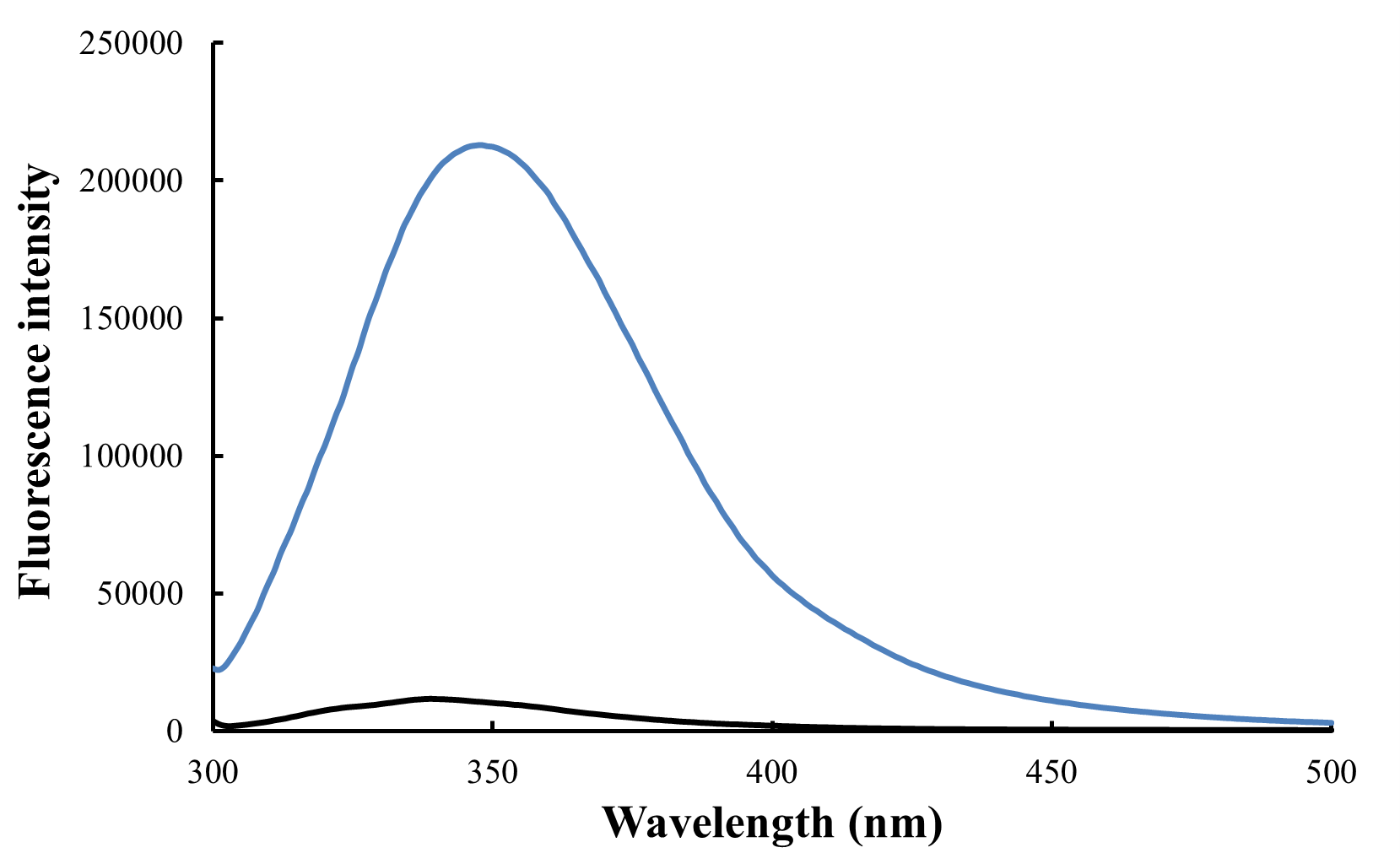


**α-amylase**

**10% DMSO**

**10% DMSO**

**(g)**

**Figure S7.** The fluorescent spectra of α-amylase in the absence and presence of TA (**a**), RA (**b**), 5-CSA (**c**), SA (**d**), CA (**e**), D (**f**), respectively. (**g**) The fluorescence spectra of α-amylase in the presence of 10% DMSO and that of 10% DMSO itself.


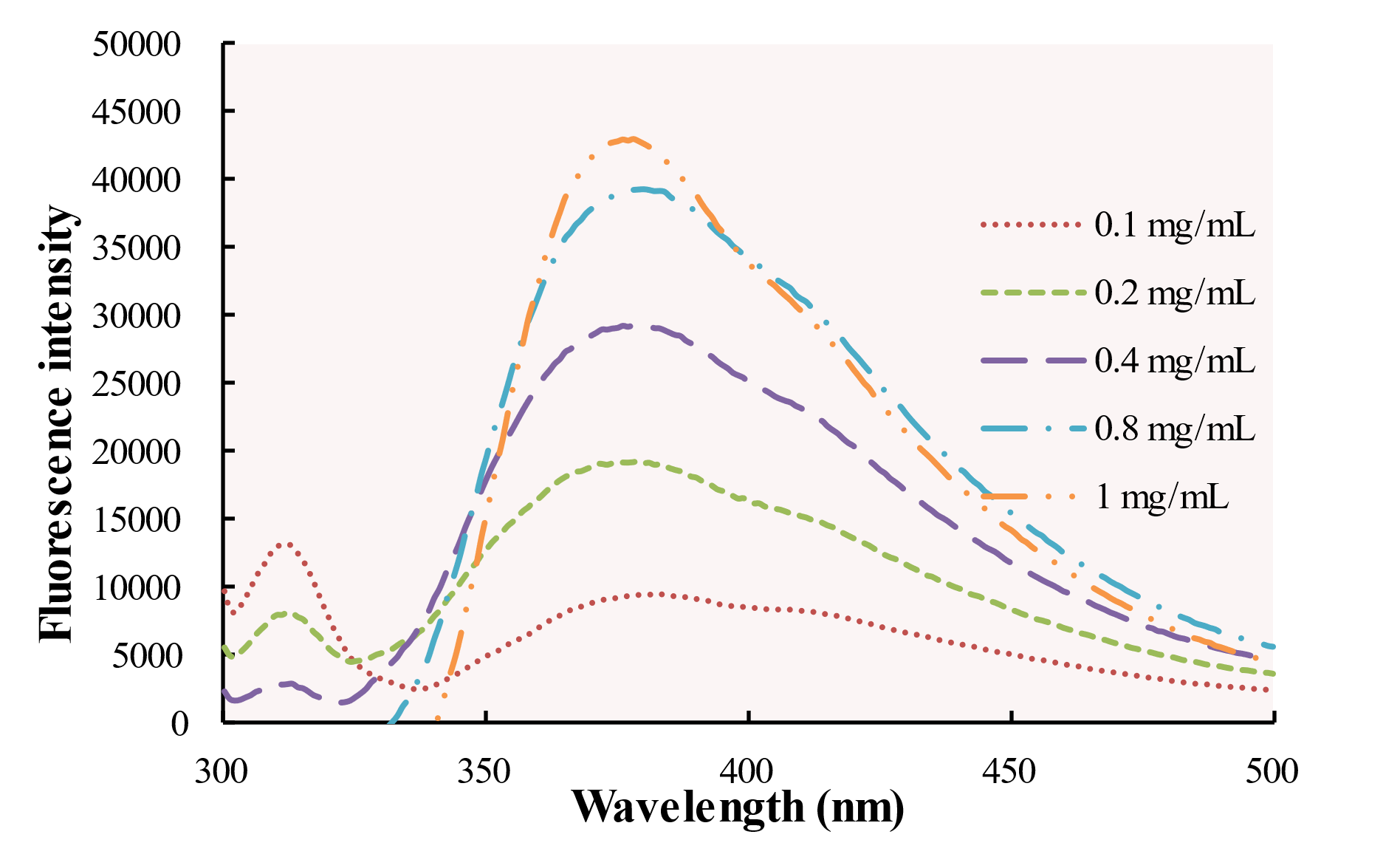


**(a)**

**TA**

**concentration**


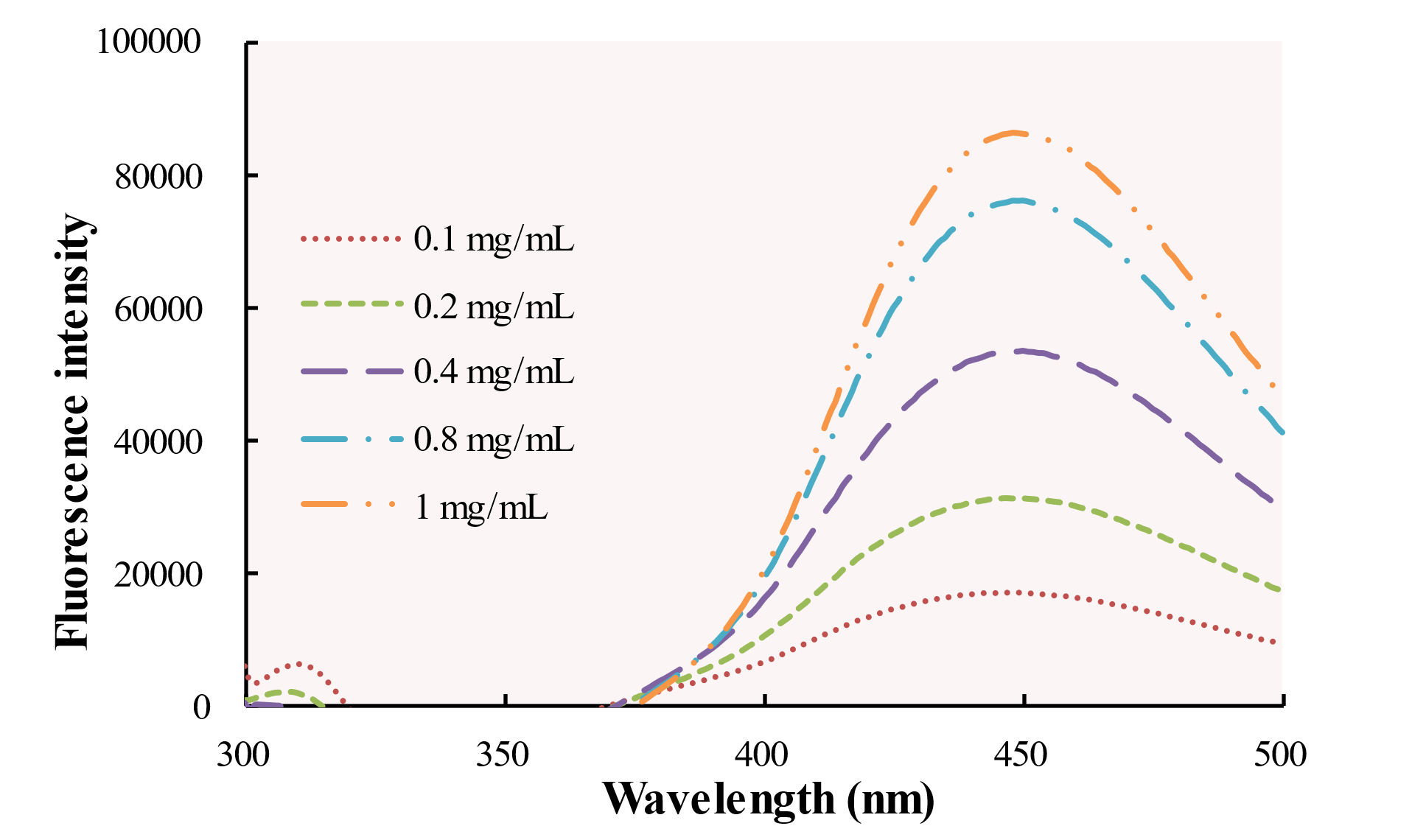


**(b)**

**RA**

**concentration**


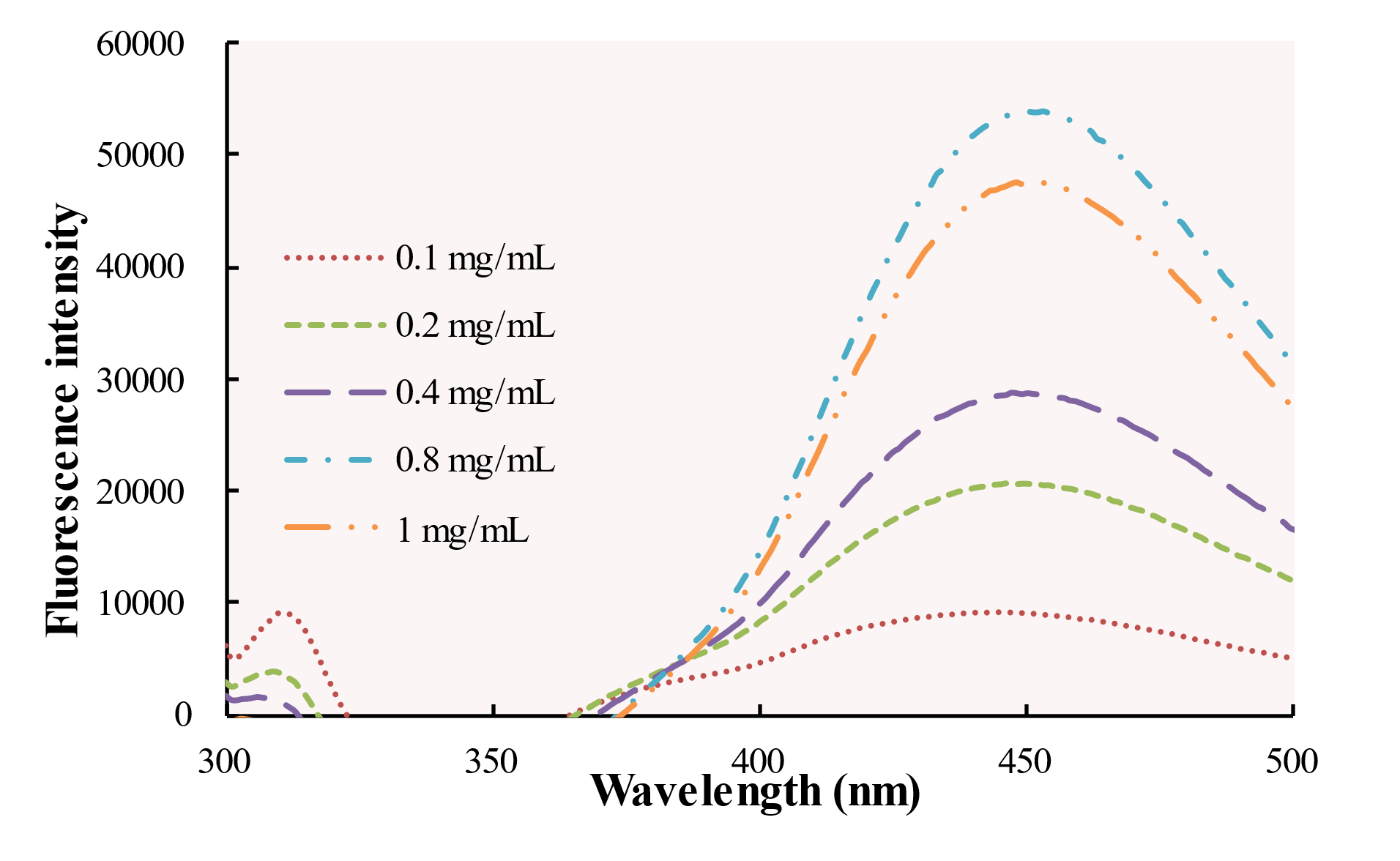


**(c)**

**5-CSA**

**concentration**


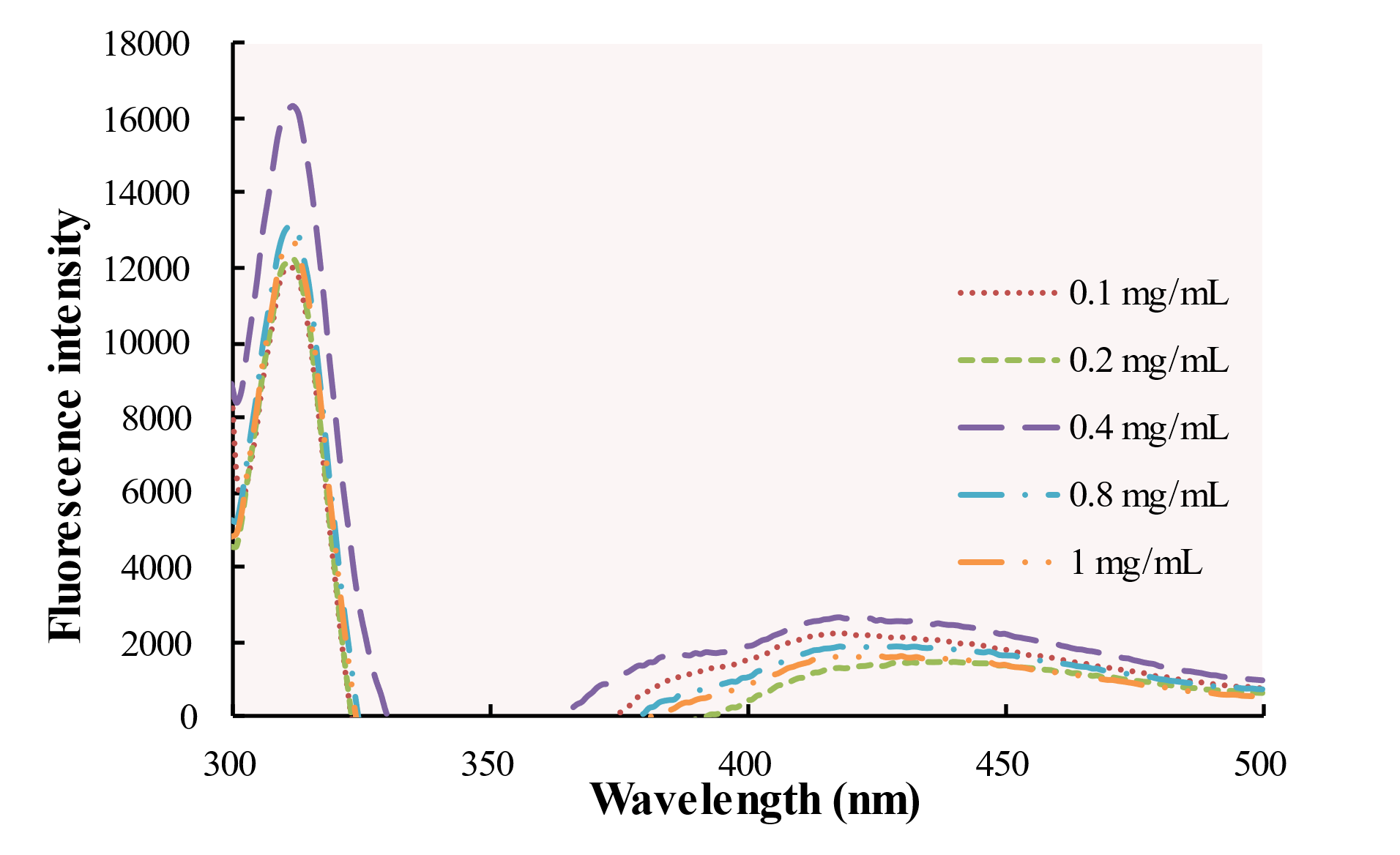


**(d)**

**SA**

**concentration**


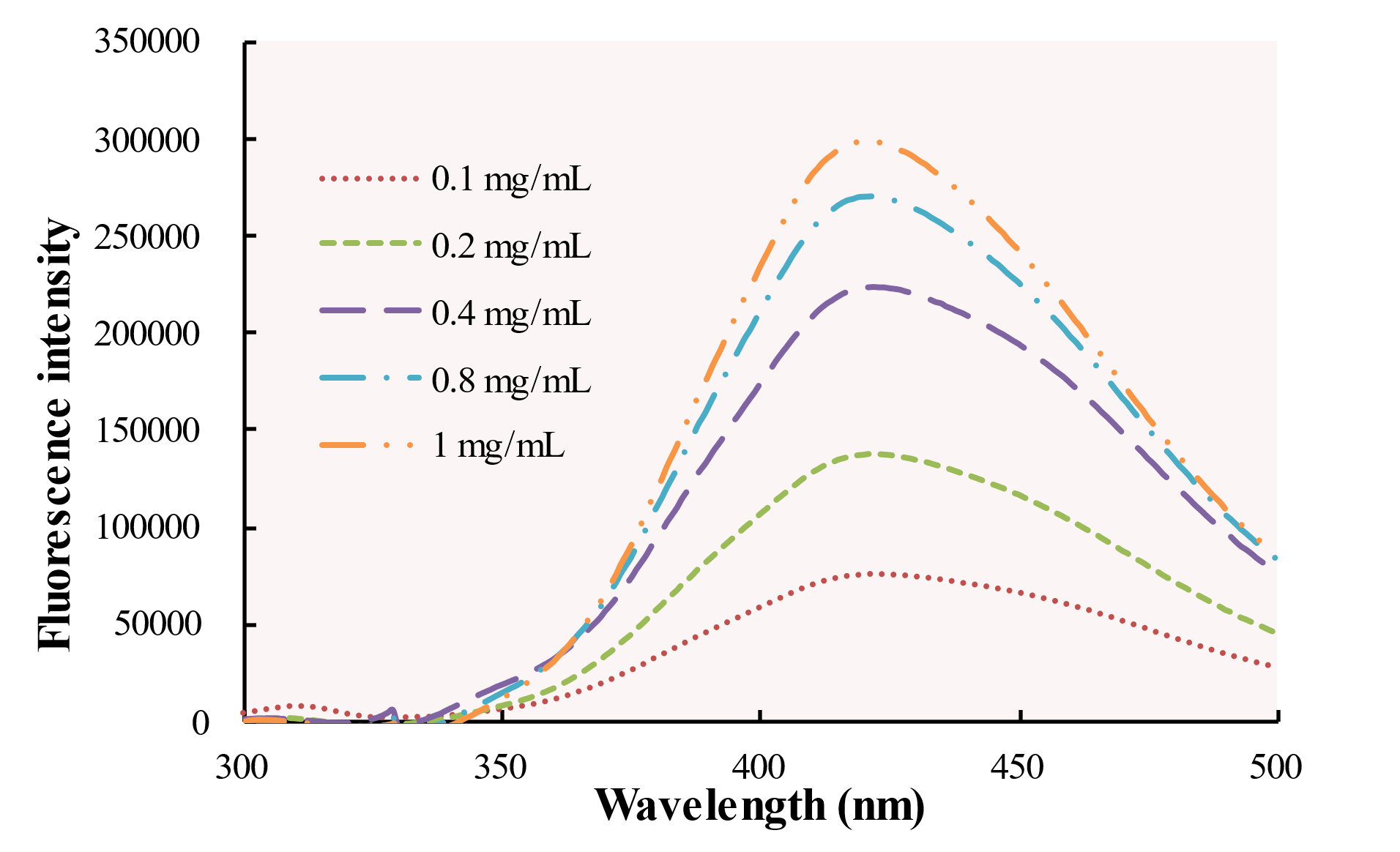


**(e)**

**CA**

**concentration**


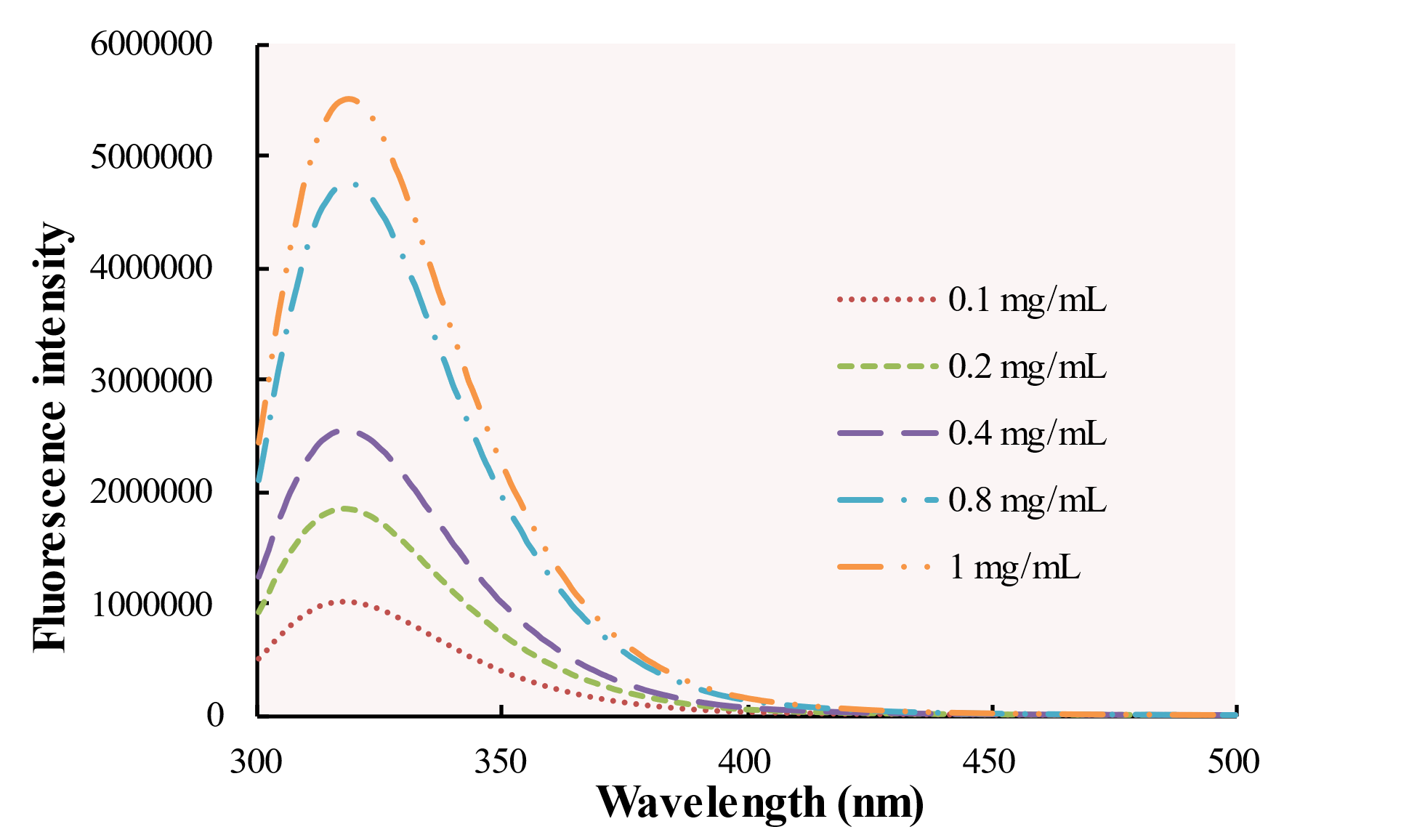


**(f)**

**D**

**concentration**

**Figure S8.** The fluorescent spectra of polyphenols with series of concentrations including TA (**a**), RA (**b**), 5-CSA (**c**), SA (**d**), CA (**e**) and D (**f**), respectively.


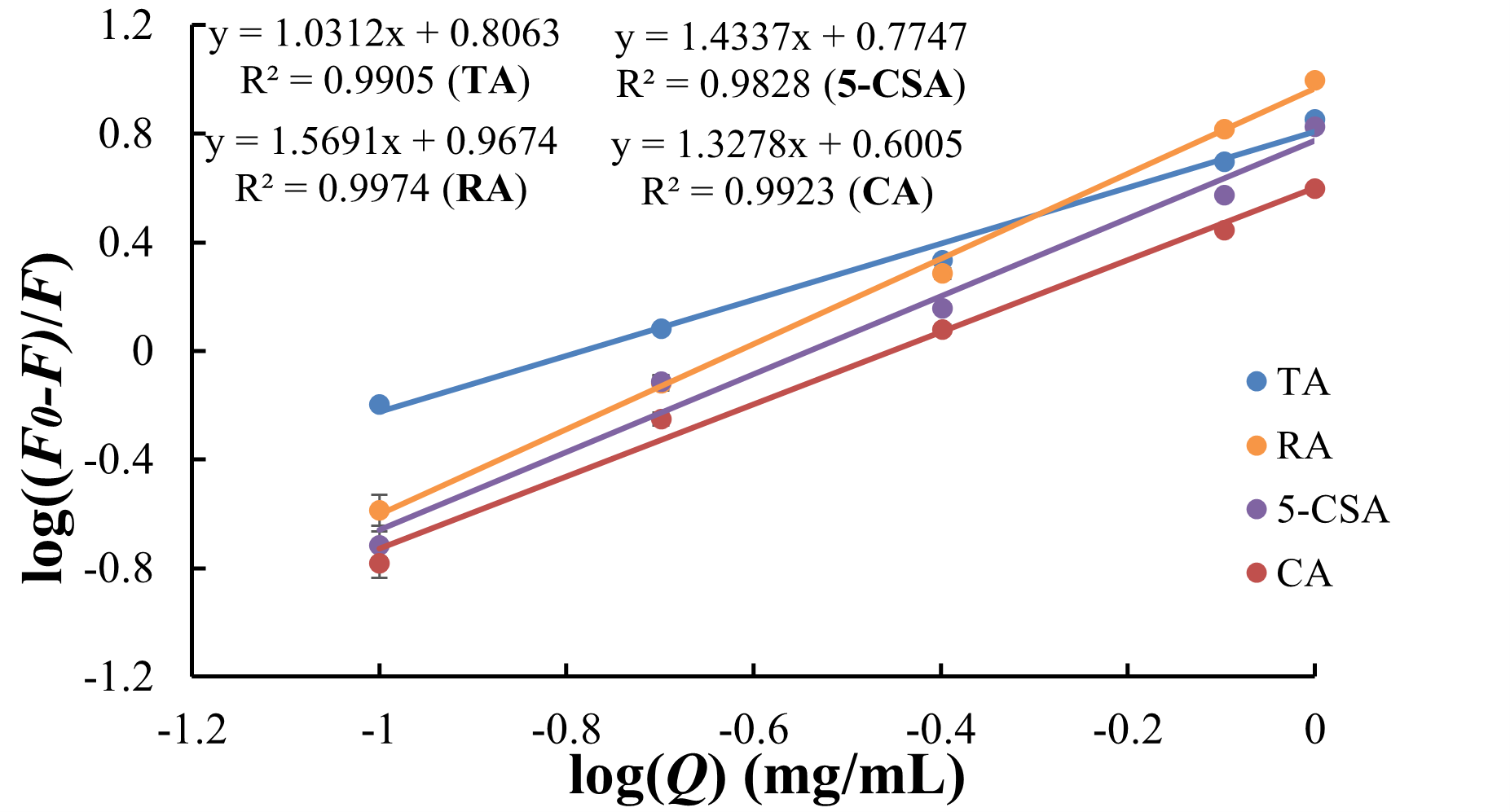

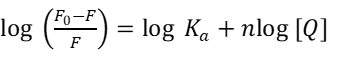


**Figure S9.** The double-log modified Stern-Volmer plot applied to analyze the quenching effects of polyphenols, from which the apparent binding constant *K*_a_ (M^−1^) and the number of binding sites *n* were obtained.


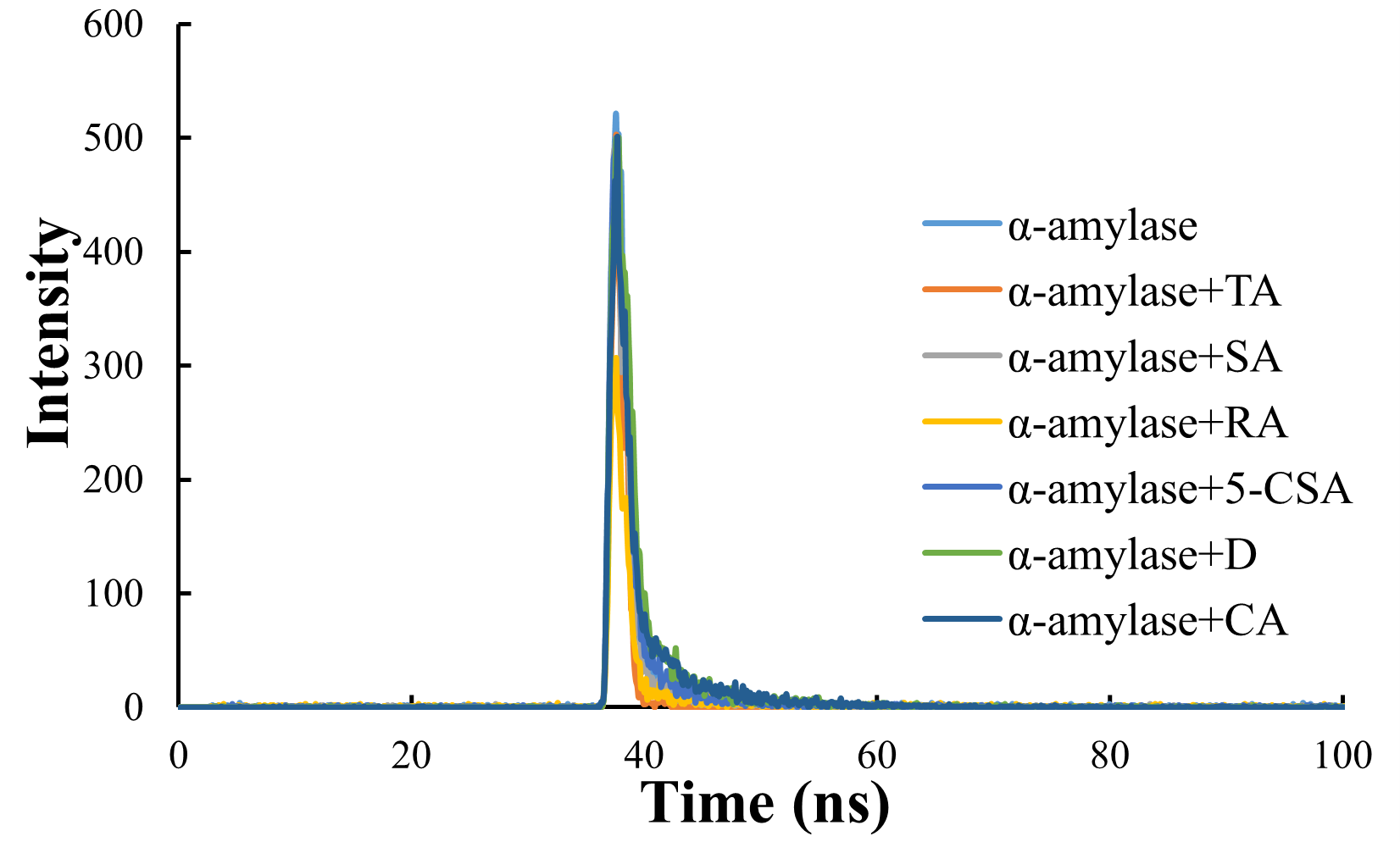


**Figure S10.** Fluorescence decay traces of α-amylase in the absence and presence of polyphenols.


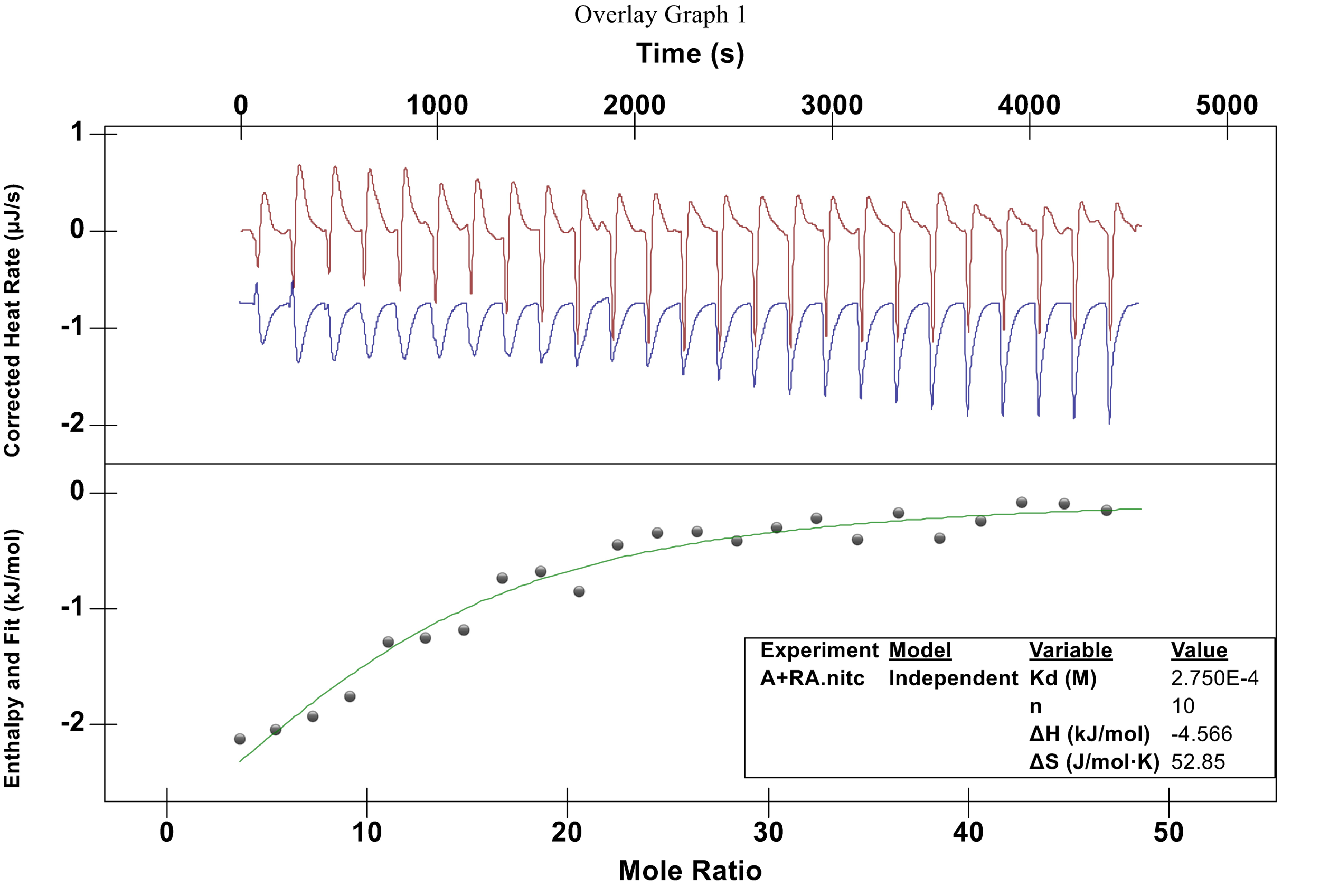


$Q_{i}=\frac{10[M](-4.566)V_{0}}{2}\left\{ 1+\frac{\left[ P \right]}{10\left[ M \right]}+\frac{2.750\times{10}^{-4}}{10\left[ M \right]}-\sqrt{\left( 1+\frac{\left[ P \right]}{10\left[ M \right]}+\frac{2.750\times{10}^{-4}}{10\left[ M \right]} \right)^{2}-4\frac{\left[ P \right]}{10\left[ M \right]}} \right\}$

**Titration of RA to α-amylase**

**Titration of RA to PBS**

**(a)**


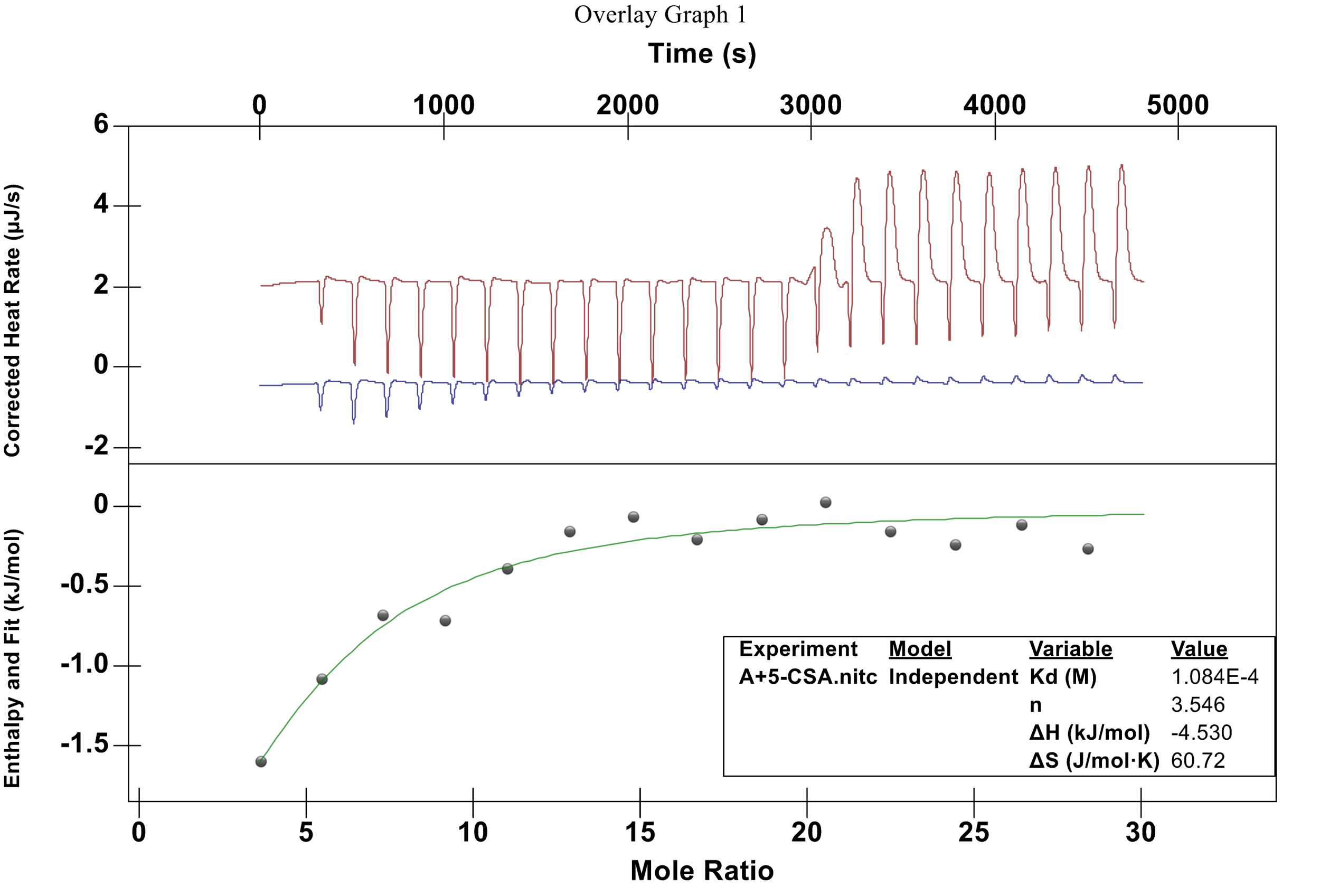


$Q_{i}=\frac{3.546[M](-4.53)V_{0}}{2}\left\{ 1+\frac{\left[ P \right]}{3.546\left[ M \right]}+\frac{1.084\times{10}^{-4}}{3.546\left[ M \right]}-\sqrt{\left( 1+\frac{\left[ P \right]}{3.546\left[ M \right]}+\frac{1.084\times{10}^{-4}}{3.546\left[ M \right]} \right)^{2}-4\frac{\left[ P \right]}{3.546\left[ M \right]}} \right\}$

**Titration of 5-CSA to PBS**

**Titration of 5-CSA to α-amylase**


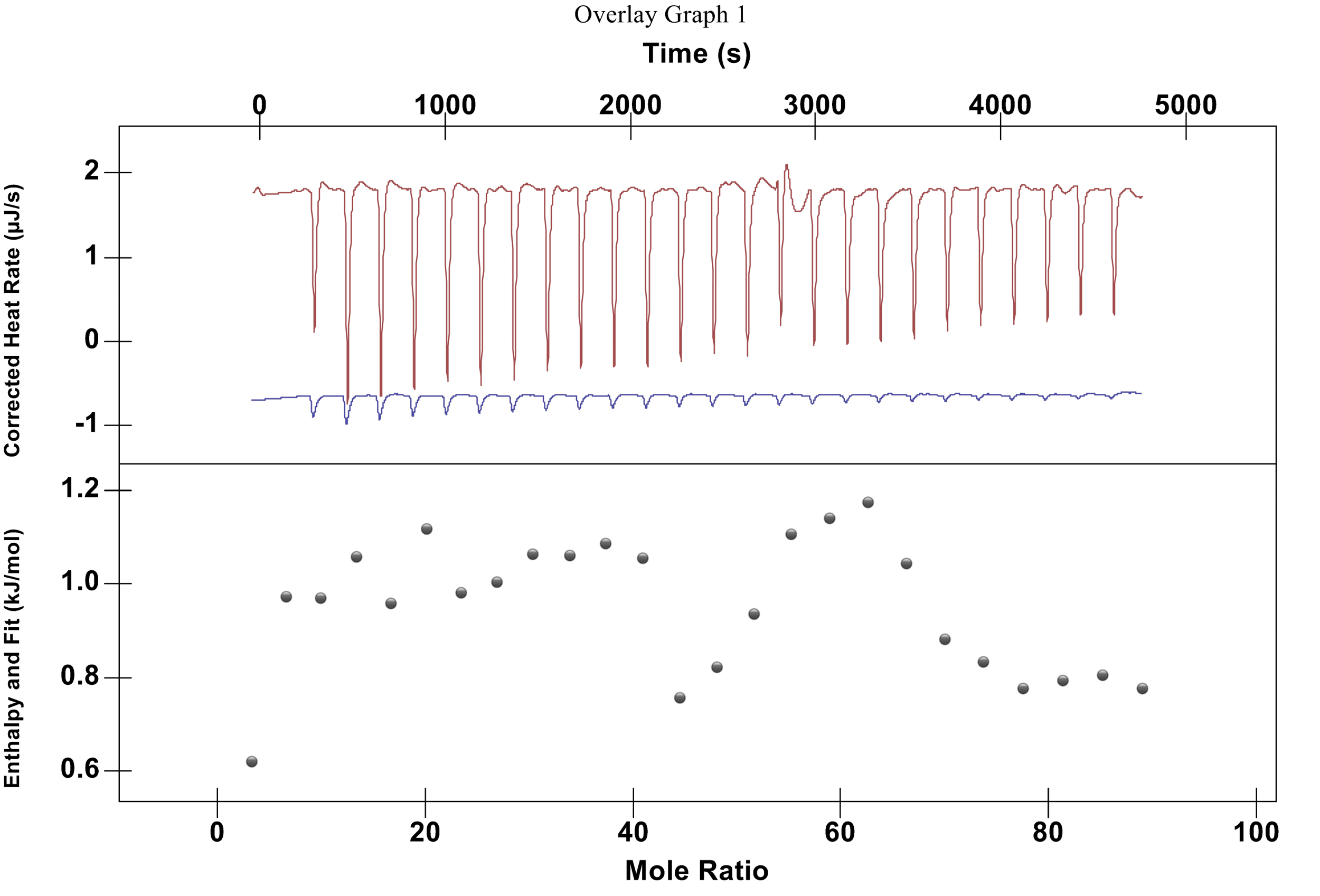


**(d)**

**Titration of D to PBS**

**Titration of D to α-amylase**

**Almost no energy changes or cannot be fitted**


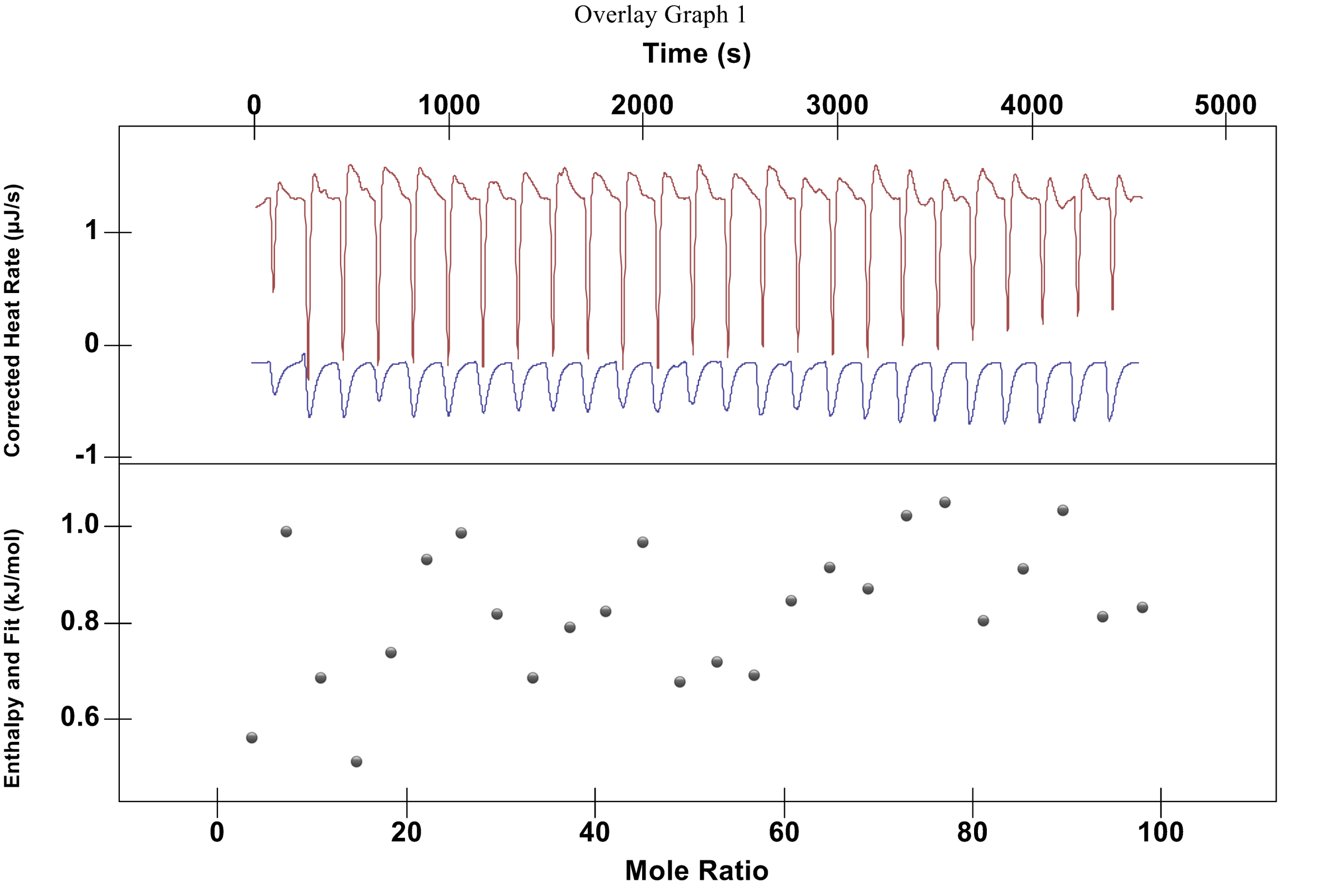


**(c)**

**Titration of CA to PBS**

**Titration of CA to α-amylase**

**Almost no energy changes or cannot be fitted**

**(b)**


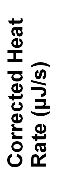

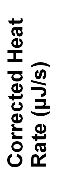

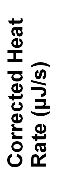

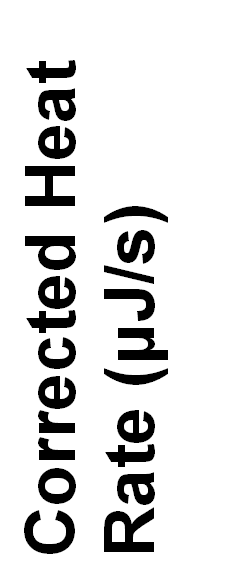

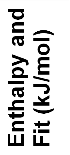

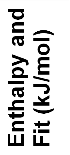

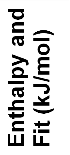

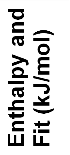


**Figure S11.** The profiles of the thermodynamic information on ITC titration of RA (**a**), 5-CSA (**b**), CA (**c**) and D (**d**) to α-amylase, respectively. The correlations between the enthalpy changes and molar ratios of polyphenols (RA and 5-CSA) to α-amylase were fitted using the independent (single-site) binding model. The data of CA and D were not fitted due to almost no energy changes in the titration process.

**(b)**


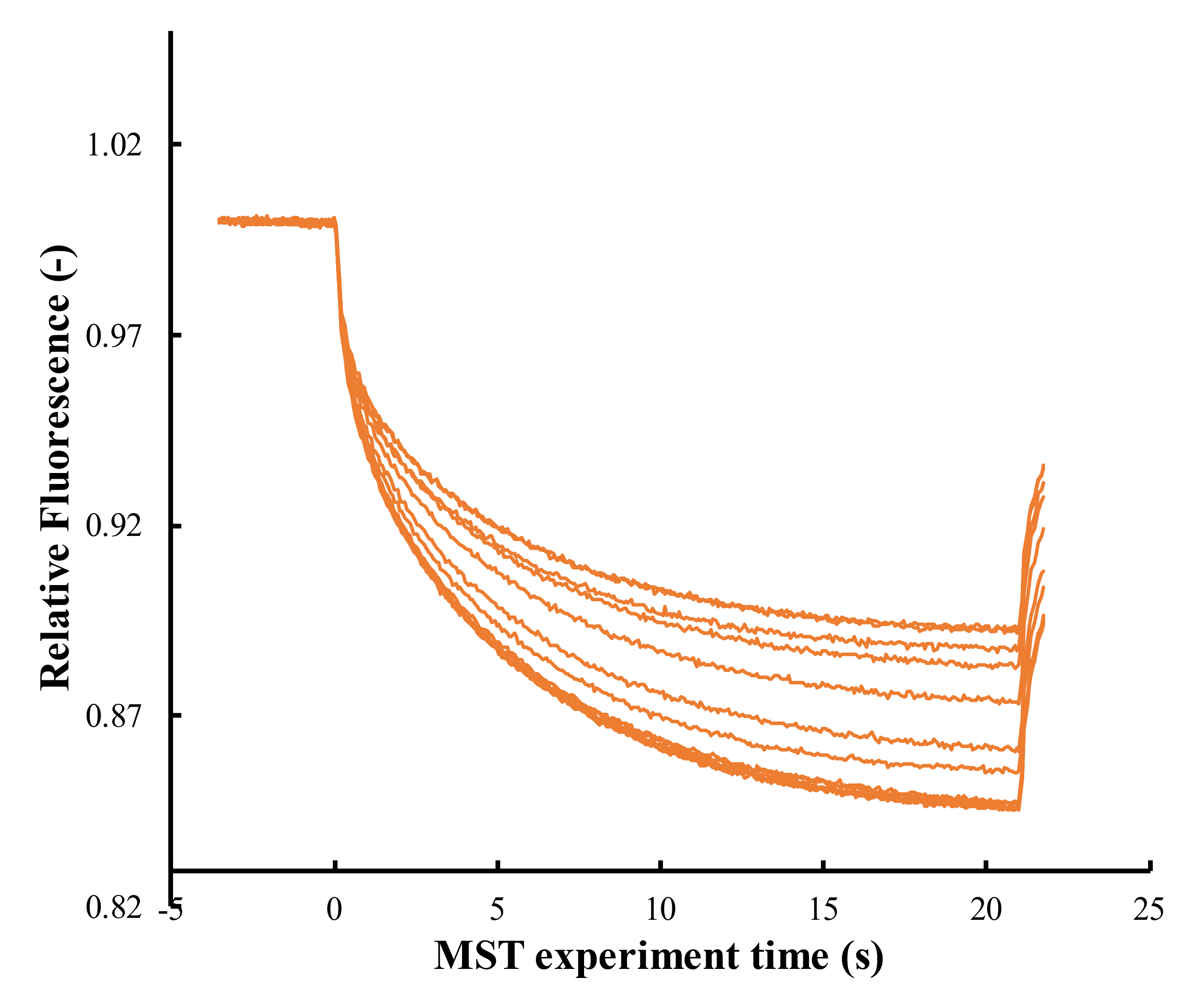


**RA + α-amylase**

**(c)**


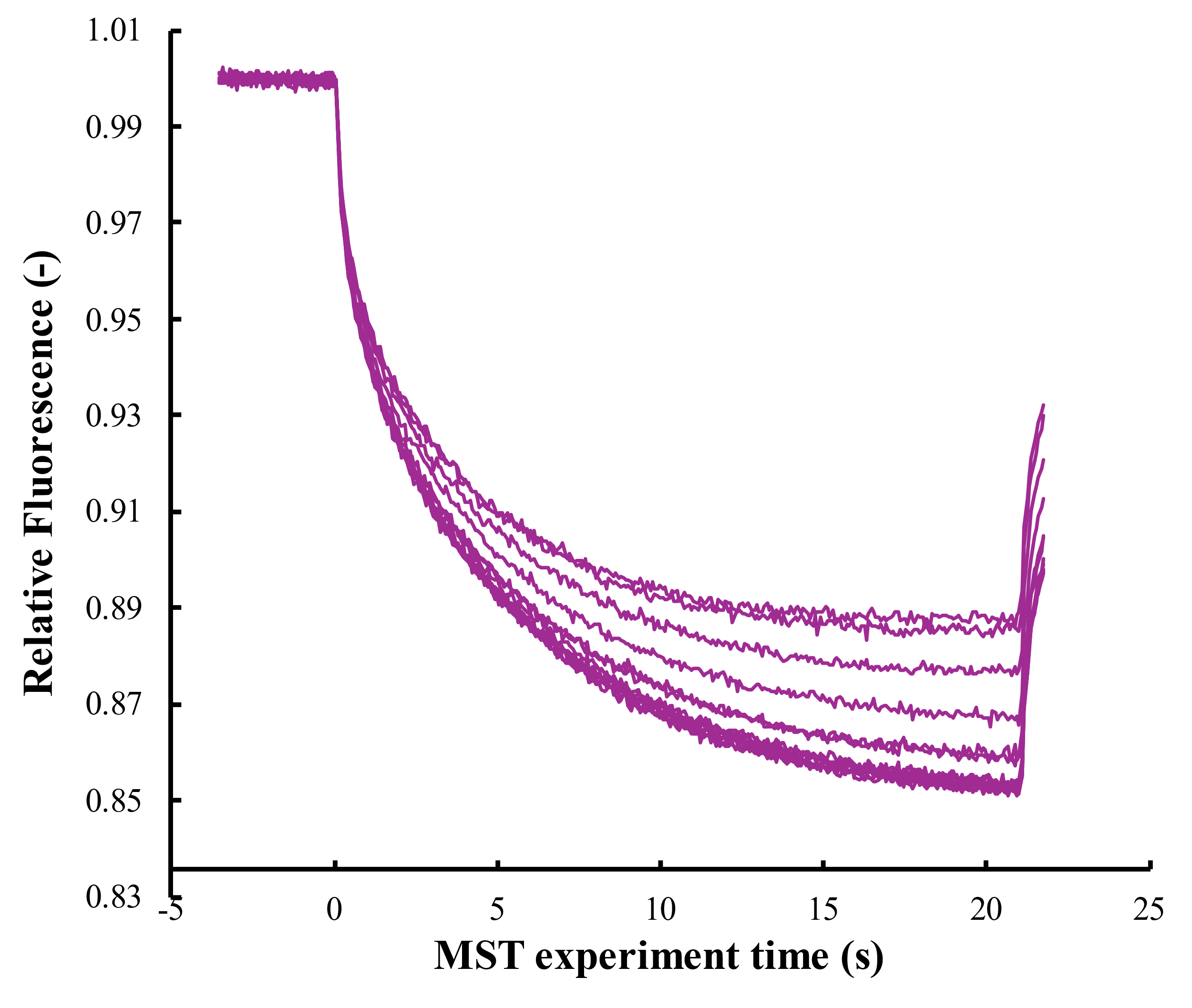


**5-CSA + α-amylase**


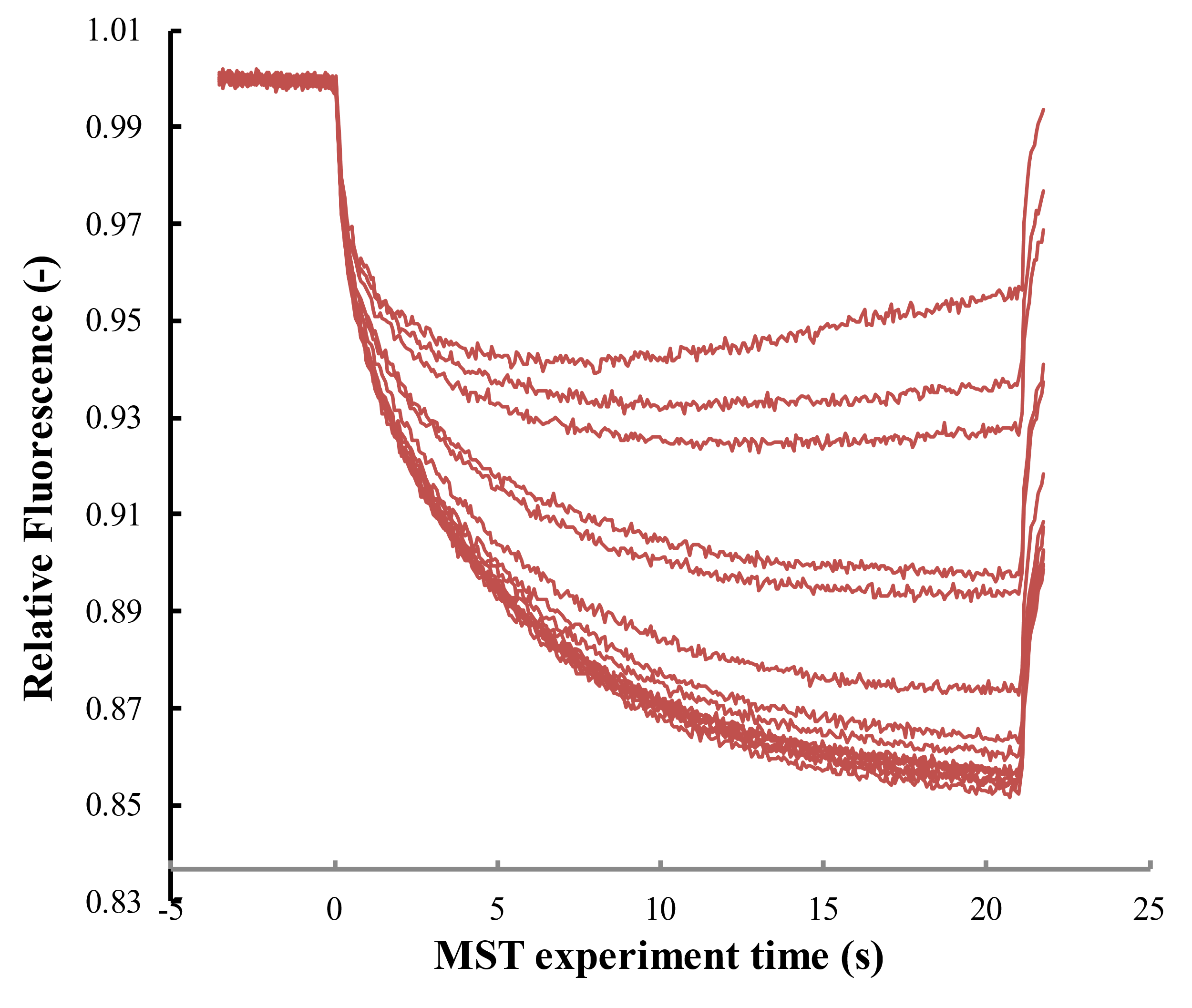


**CA + α-amylase**

**(d)**


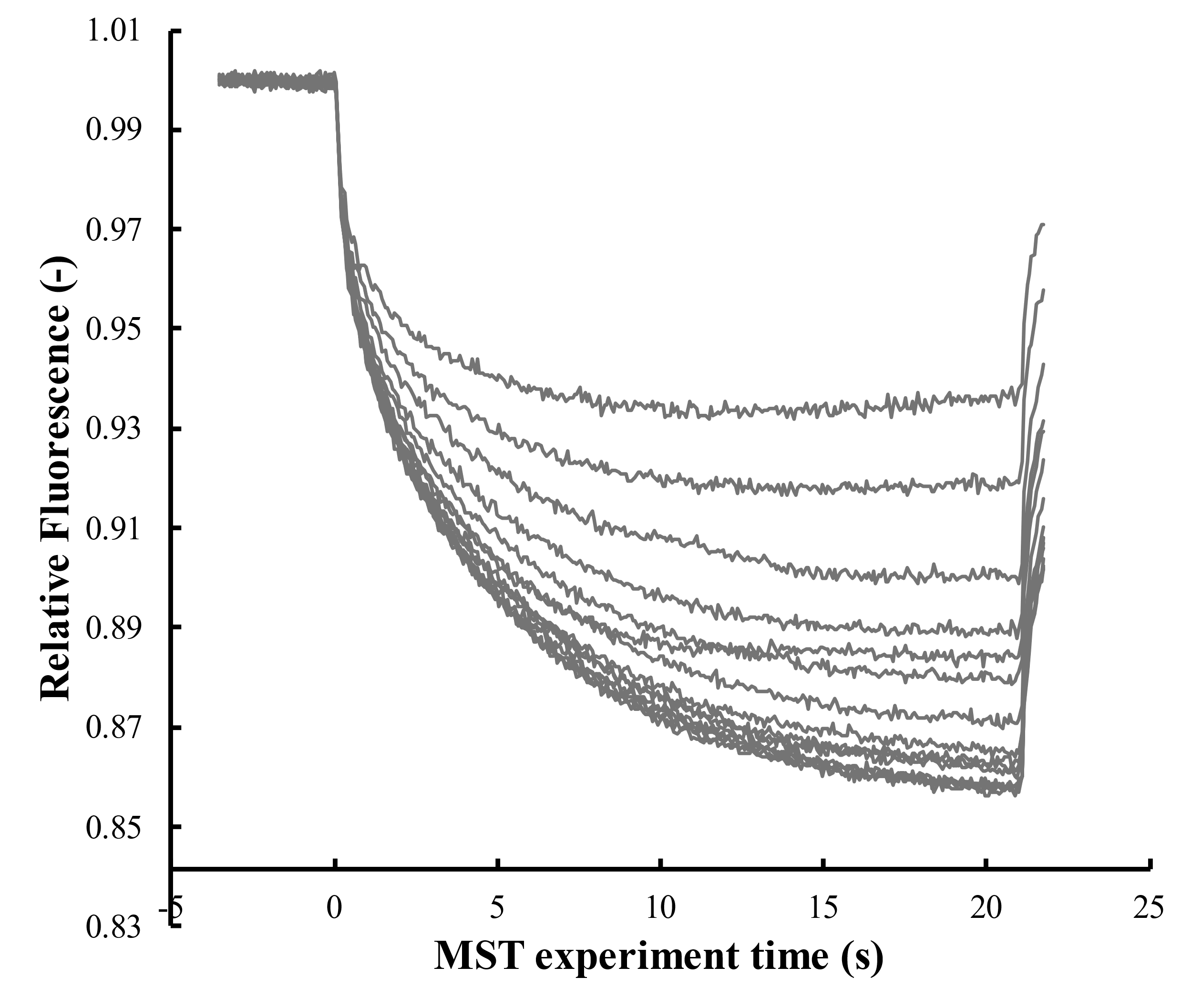


**D + α-amylase**

**(a)**

**Figure S12.** The MST time trace of labelled enzyme fluorescence in the presence of RA (**a**), 5-CSA (**b**), CA (**c**) and D (**d**) with different polyphenol concentrations, respectively.


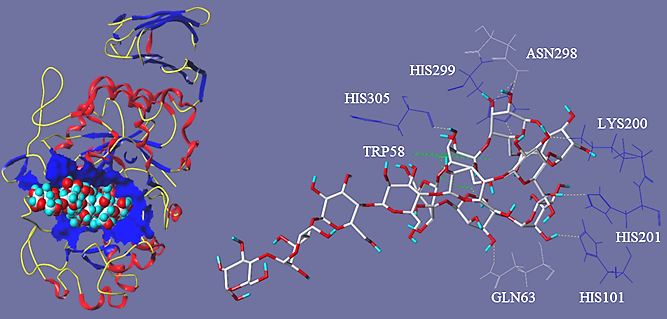


Simplified amylose

C_72_H_122_O_61_


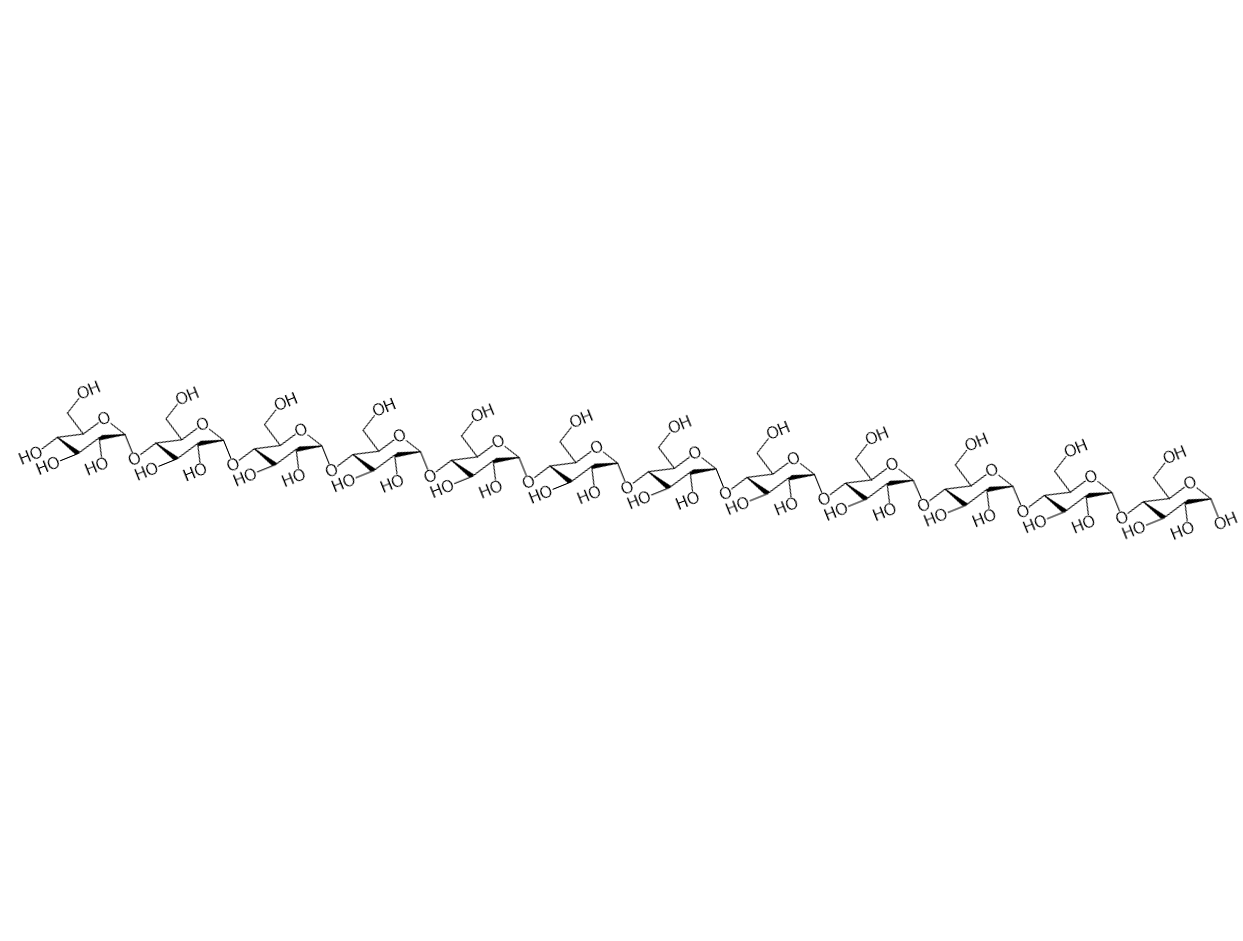


α-1,4 bonds

**(b)**

304

**Gly**

**His**

**Gly**

**Ala**

**Gly**

**Gly**

309

Flexible loop


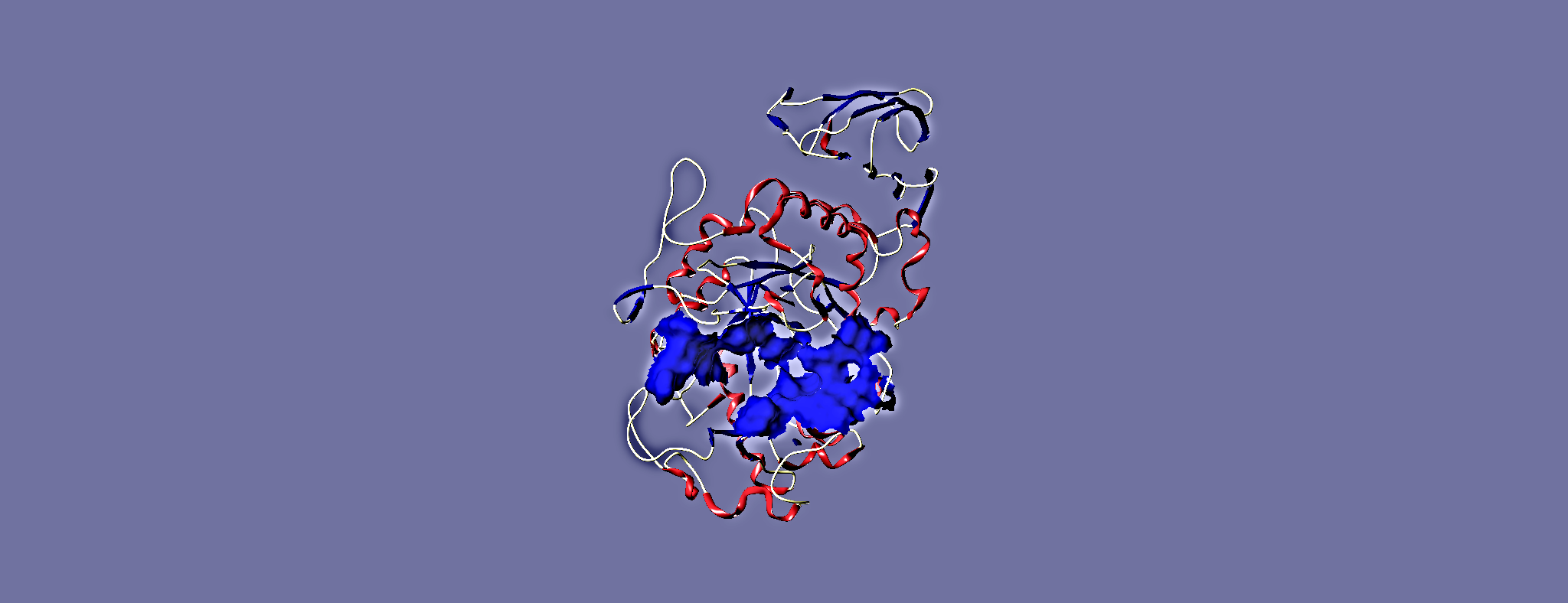


**Active site selected for polyphenols exhibiting typical and partially competitive inhibition characteristic**


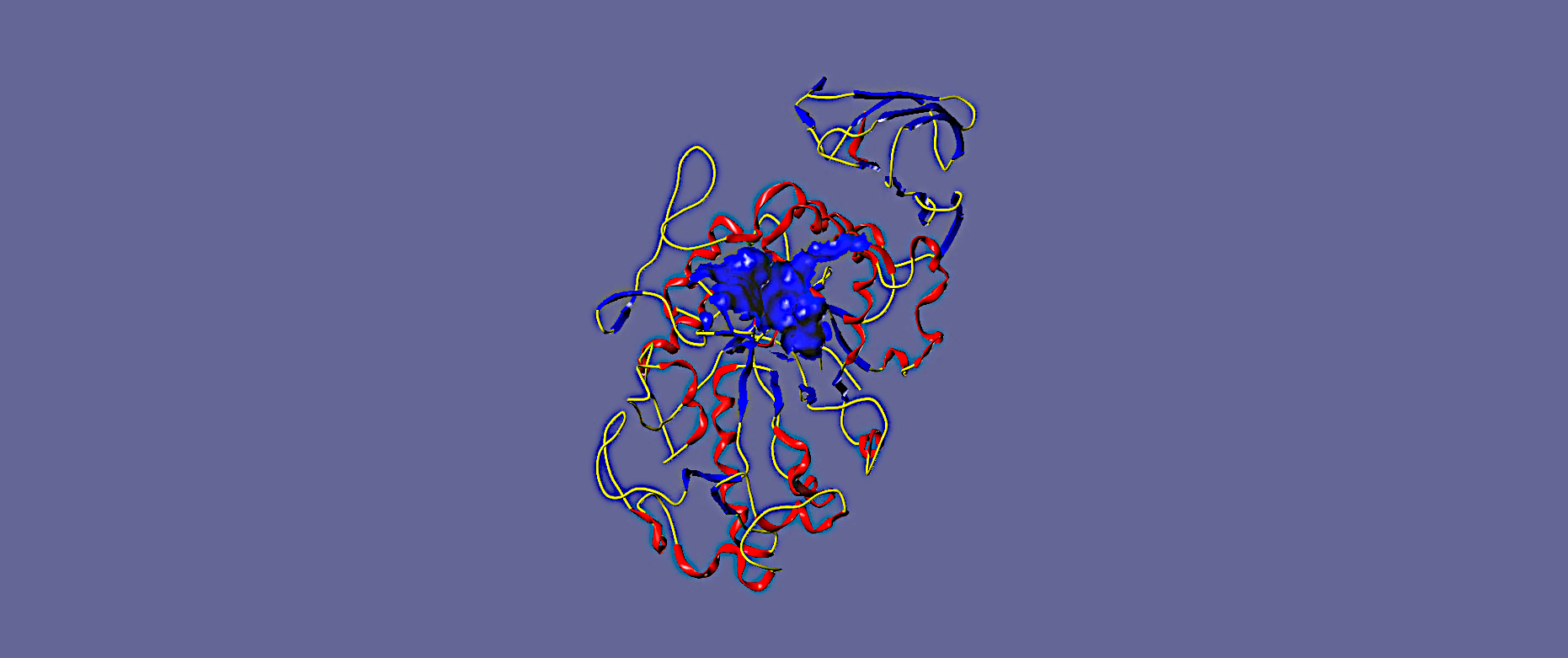


**Non-active site containing Gly^304^-Gly^309^ loop selected for polyphenols exhibiting typical and partially uncompetitive inhibition characteristic**

**(a)**

304

309


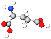

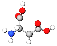

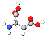


**Gly**

**His**

**Gly**

**Ala**

**Gly**

**Gly**

**Glu^233^**

**Asp^197^**

**Asp^300^**

**Figure S13.** (**a**) The selected docking site including the active pocket of α-amylase and a non-active site containing the flexible loop (Gly^304^-Gly^309^) for simulating the interactions between α-amylase and polyphenols. (**b**) The docking results of simplified amylose with α-amylase, which formed the interactions with the flexible Gly^304^-Gly^309^ loop.

**Table S1**. The detail constants for characterization of the inhibitory effects of polyphenols against α-amylase.

| Polyphenols | IC_50_ (mg/mL) | *K_ic_* (mg/mL) | | 1/*K_ic_* (mL/mg) | | *K_iu_* (mg/mL) | | 1/*K_iu_* (mL/mg) | |
| --- | --- | --- | --- | --- | --- | --- | --- | --- | --- |
|  | GalG2CNP | GalG2CNP | NMS | GalG2CNP | NMS | GalG2CNP | NMS | GalG2CNP | NMS |
| TA | 8.64×10^-3^ ± 5.82×10^-4^ | 5.67×10^-3^ ± 1.97×10^-3^ | 4.11×10^-3^ ± 2.01×10^-3^ | 176.37 ± 43.31 | 243.31± 79.18 | - | - | - | - |
| SA | NA | ND | - | ND | - | ND | 0.33 ± 0.023 | ND | 3.03 ± 0.23 |
| RA | 0.13 ± 0.001 | 0.097 ± 0.043 | 0.015 ± 0.006 | 10.31 ± 2.42 | 66.67 ± 18.12 | 0.074 ± 0.036 | 0.043 ± 0.013 | 13.51 ± 2.69 | 23.26 ± 10.84 |
| 5-CSA | 0.18 ± 0.016 | 0.37 ± 0.081 | ND | 2.70 ± 0.79 | ND | 0.23 ± 0.082 | ND | 4.37 ± 2.14 | ND |
| D | NA | ND | ND | ND | ND | ND | ND | ND | ND |
| CA | 0.71 ± 0.021*^a^*^)^ | ND | ND | ND | ND | ND | ND | ND | ND |

Note: ^‘^NA^’^ means the constant was not available because the data could not be fitted. ^‘^ND^’^ means not detected due to the very weak inhibition. ‘-’ means the present inhibition type was not shown in inhibition kinetics. *^a^*^)^ The practical IC_50_ value of CA was thought to be smaller than the calculated one here taking its unsatisfactory solubility into account.

**Table S2.** The pH values of the used polyphenols at 20 mg/mL and their p*K*a values collected from the ChemicalBook website.

| Polyphenols | pH (20 mg/mL) | p*K*a |
| --- | --- | --- |
| RA | 2.48 | 2.78 |
| SA | 3.07 | 5.19 |
| 5-CSA | 3.75 | 4.27 |
| CA | 4.33 | 4.58 |
| TA | 4.40 | 6.00 |

**Table S3.** The detail constants for characterization of the binding interactions between α-amylase and polyphenols.

| Polyphenols | *K*_FQ_ (M^-1^) | *τ^app^* (ns) ^a)^ | *k*_q_×10^11^ (M^-1^s^-1^) | *n* | *K_a_* (M^-1^) | *K*_d_^itc^ (M) | *K*_itc_ (M^-1^) | *K*_d_^mst^ (M) | *K*_mst_ (M^-1^) |
| --- | --- | --- | --- | --- | --- | --- | --- | --- | --- |
| TA | 12042.28 ± 48.65 | 1.12 ± 3.18×10^-4^ | 116.69 ± 0.47 | 1.03 ± 0.003 | 10947.39 ± 56.64 | 1.86×10^-5^ ± 7.40×10^-6^ | 6.03×10^4^ ± 2.11×10^4^ | 9.82×10^-5^ ± 2.28×10^-5^ | 1.07×10^4^ ± 2.41×10^3^ |
| SA | NA | 1.05 ± 2.94×10^-4^ | NA | NA | NA | 3.50×10^-4^ ± 6.76×10^-5^ | 2.93×10^3^ ± 4.71×10^2^ | 0.27 ± 0.04 | 3.79 ± 0.66 |
| RA | 844.67 ± 16.06 | 1.06 ± 3.32×10^-4^ | 8.18 ± 0.15 | 1.53 ± 0.039 | 3418.06 ± 75.53 | 2.75×10^-4^ ± 9.09×10^-5^ | 3.94×10^3^ ± 1.12×10^3^ | 3.06×10^-3^ ± 8.19×10^-4^ | 351.99 ± 98.94 |
| 5-CSA | 660.59 ± 13.33 | 1.13 ± 1.19×10^-3^ | 6.40 ± 0.12 | 1.39 ± 0.035 | 2029.09 ± 27.32 | 1.08×10^-4^ ± 3.80×10^-5^ | 1.01×10^4^ ± 3.07×10^3^ | 5.37×10^-3^ ± 1.08×10^-3^ | 194.28 ± 40.22 |
| D | NA | 1.38 ± 2.68×10^-4^ | NA | NA | NA | NA | NA | 0.025 ± 0.005 | 41.15 ± 9.54 |
| CA | 651.34 ± 19.41 | 1.17 ± 1.56×10^-3^ | 6.31 ± 0.18 | 1.28 ± 0.040 | 728.19 ± 10.13 | NA | NA | 6.91×10^-3^ ± 7.79×10^-4^ | 146.41 ± 16.24 |

Note: ^‘^NA^’^ means the constant was not available because the data could not be fitted. ^a)^ The *τ*_0_ value of α-amylase in the absence of polyphenols was 1.03 ± 8.31×10^-4^.
